# An accessible insight into genetic findings for transplantation recipients with suspected genetic kidney disease

**DOI:** 10.1038/s41525-021-00219-3

**Published:** 2021-07-02

**Authors:** Zhigang Wang, Hongen Xu, Tianchao Xiang, Danhua Liu, Fei Xu, Lixiang Zhao, Yonghua Feng, Linan Xu, Jialu Liu, Ye Fang, Huanfei Liu, Ruijun Li, Xinxin Hu, Jingyuan Guan, Longshan Liu, Guiwen Feng, Qian Shen, Hong Xu, Dmitrij Frishman, Wenxue Tang, Jiancheng Guo, Jia Rao, Wenjun Shang

**Affiliations:** 1 Department of Kidney Transplantation, First Affiliated Hospital of Zhengzhou University, Zhengzhou, Henan China; 2 Precision Medicine Center of Zhengzhou University, Academy of Medical Sciences, Zhengzhou University, Zhengzhou, Henan China; 3 Department of Nephrology, Children’s Hospital of Fudan University, Shanghai, China; 4 Shanghai Key Lab of Birth Defect, Children’s Hospital of Fudan University, Shanghai, China; 5 The Second Affiliated Hospital of Zhengzhou University, Zhengzhou, Henan China; 6 Organ Transplant Center, The First Affiliated Hospital of Sun Yat-sen University, Guangzhou, China; 7 Department of Bioinformatics, Technische Universität München, Freising, Germany; 8 Henan Institute of Medical and Pharmaceutical Sciences, Zhengzhou University, Zhengzhou, Henan China; 9 State Key Laboratory of Medical Neurobiology, Institutes of Brain Science and School of Basic Medical Science, Fudan University, Shanghai, China

**Keywords:** End-stage renal disease, Genetics research

## Abstract

Determining the etiology of end-stage renal disease (ESRD) constitutes a great challenge in the context of renal transplantation. Evidence is lacking on the genetic findings for adult renal transplant recipients through exome sequencing (ES). Adult patients on kidney transplant waitlist were recruited from 2017 to 2019. Trio-ES was conducted for the families who had multiple affected individuals with nephropathy or clinical suspicion of a genetic kidney disease owing to early onset or extrarenal features. Pathogenic variants were confirmed in 62 from 115 families post sequencing for 421 individuals including 195 health family members as potential living donors. Seventeen distinct genetic disorders were identified confirming the *priori* diagnosis in 33 (28.7%) families, modified or reclassified the clinical diagnosis in 27 (23.5%) families, and established a diagnosis in two families with ESRD of unknown etiology. In 14.8% of the families, we detected promising variants of uncertain significance in candidate genes associated with renal development or renal disease. Furthermore, we reported the secondary findings of oncogenes in 4.4% of the patients and known single-nucleotide polymorphisms associated with pharmacokinetics in our cohort to predict the drug levels of tacrolimus and mycophenolate. The diagnostic utility of the genetic findings has provided new clinical insight in most families that help with preplanned renal transplantation.

## Introduction

Chronic kidney disease (CKD) affects over 850 million individuals worldwide. Recent predictions by the Institute of Health Metrics and Evaluations indicated that by 2040 CKD will be the fifth leading cause of life years lost on a global scale^1,2^. A positive family history is reported by around 30% of patients with CKD, and familial clustering is a common phenomenon in patients with end-stage renal disease (ESRD)^3–5^. Approximately 15% of patients with ESRD do not have a primary renal disease diagnosis and are therefore labeled as CKD with unknown origin^6^. Making a correct diagnosis in these patients may have therapeutic implications. Knowledge of the underlying kidney disease is crucial for ESRD management in the context of transplantation, as the primary etiology may affect graft survival in terms of recurrence and or rejection^7–10^. Moreover, a genetic diagnosis may be of pivotal importance for family counseling and in the setting of kidney transplantation, particularly when living related donation is involved^9,11^.

In recent years, we have gained a better understanding of the genetic landscape of CKD in children and young adults through next-generation sequencing. Approximately 500 monogenic causes of CKD have been identified^12^. It has been shown that a monogenic disease-causing variant can be identified in 10–36% of adults with CKD^7,12,13^. A few studies reported the initial experience on genetic diagnostic panel for kidney transplantation cohort^8,14^. However, genetic testing is currently not performed on a regular basis in patients with ESRD waiting for kidney transplantation. In this study, we aimed to unravel the genetic diagnosis for patients with familial ESRD on the kidney transplantation waitlist. We hypothesize that genetic causes in adults are underrecognized, particularly in patents with a positive family history or patient cohorts with unknown etiology. Improving the diagnosis of primary disease in patients with ESRD can therefore have implications for adequate clinical decisions for kidney transplantation.

## Results

### Clinical characteristics

A total of 115 families with 226 affected individuals (male: female 1.4:1) were recruited from 576 family cohorts with records in the kidney transplantation registry since 2017–2019. All the information of the 576 families came from the 64 dialysis centers in 18 provinces in China. The demographic data and the geographic distribution of cases are shown in Supplementary Fig. 1. Consanguinity was observed in one family.

Among the 115 families in this cohort, 104 families had multiple affected individuals checked for the records of urinalysis, renal function, and imaging studies of the kidneys. There were three families with extrarenal features and a negative family history of renal disease. There were eight patients who had early-onset kidney disease without any extrarenal features or family history of renal disease. Among the 226 affected individuals, 75.7% (171/226) had proteinuria including 11.5% (26/226) with nephrotic proteinuria at disease onset. Abnormal image findings such as renal size or echogenicity were reported by renal ultrasound in 42.9% (97/226) of the cases. Renal biopsy was performed in 30.1% (68/226) of the affected individuals. Hearing loss was recorded in 16.8% (38/226) and vision deficiency was recorded in 4.4% (10/226). The median age at diagnosis of renal disease was 22.0 years (interquartile range [IQR] 12.8–32.0 years). The median age at genetic test was 31.0 years (IQR 22.5–48.0 years). For these families, at least one of the family members was on the transplant waitlist. At the time of transplant registration, 125 (55.3%) probands developed into ESRD, four affected family members developed into CKD stage 4, six individuals developed into CKD stage 3, and six individuals developed into CKD stage 2. The median age at first renal replacement therapy was 28.2 years (range, 7.0–79.4 years). Overall, 11.9% (27/226) of the patients in this cohort underwent renal transplantation.

Subgroups were defined as a *priori* clinical diagnosis and details are presented in the Methods section. The probands from 103 families had a primary clinical diagnosis including steroid-resistant nephrotic syndrome (SRNS, 42/115), glomerulonephritis (GN, 49/115), tubulointerstitial kidney disease (TIKD, 4/115), and congenital anomalies of the kidney and urinary tract (CAKUT, 8/115). In 12 out of the 115 families, the cause of ESRD was unknown (ESRDu). A total of 55.7% (64/115) of the families were diagnosed based on pathological findings.

### Genetic diagnosis established by exome sequencing

**Fig. 1:**
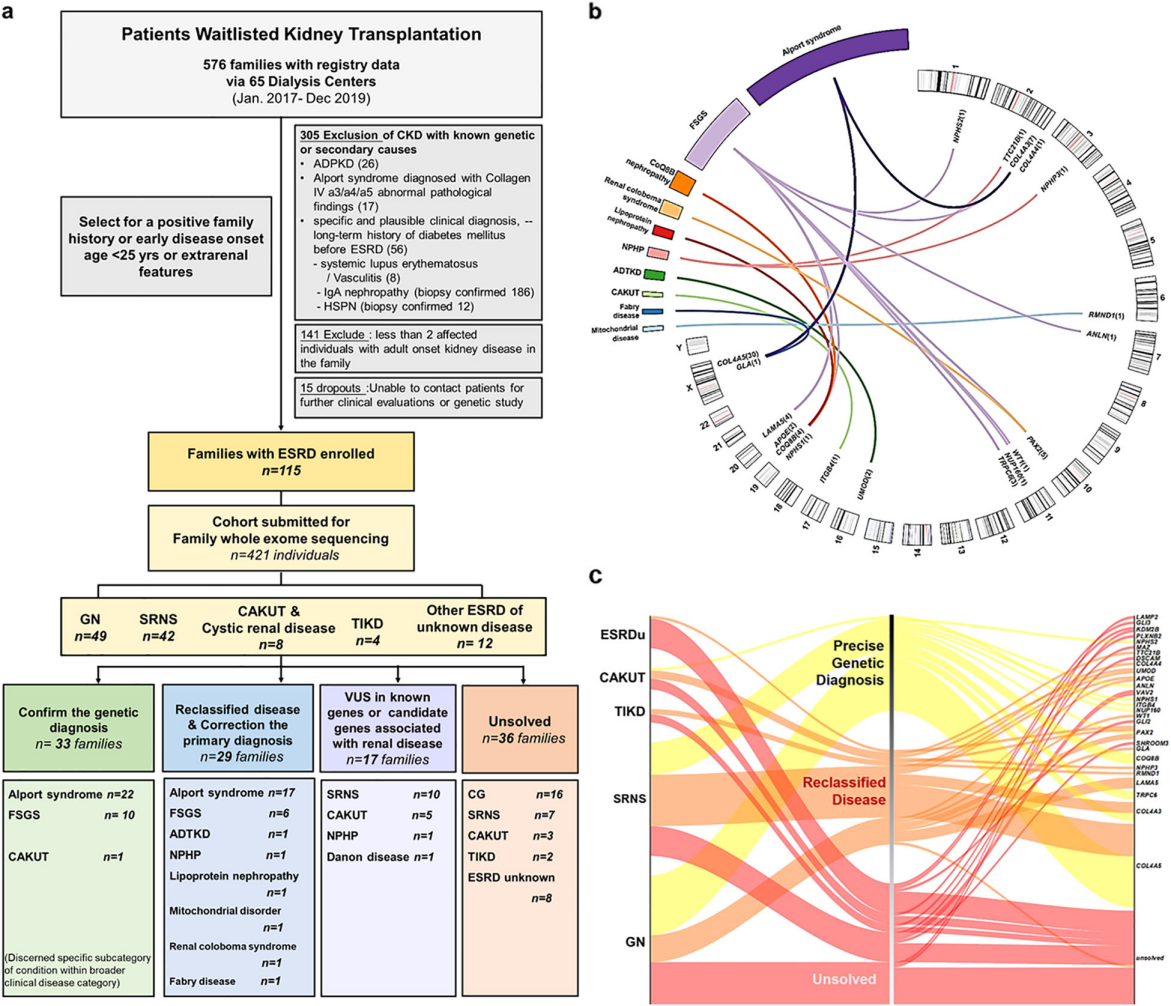
Genetic findings post exome sequencing (ES) study in families on the waitlist for renal transplantation. **a** Study design and stratification. Through evaluation of the registry information in the waiting list for transplantation, patients with a positive family history or patients with clinical suspicion of a genetic kidney disease owing to childhood early onset or extrarenal features were enrolled into the study. All the recruited families (*n* = 115) with ESRD were divided into five subgroups according to the prior clinical diagnosis of renal disease. ES was performed in 421 individuals from the 115 families (226 patients with CKD). Family-ES identified a specific underlying cause within the broader category of clinical suspected disease in 33 families, modified or reclassified the clinical diagnosis in 27 families, and established a diagnosis in two families referred with ESRD of unknown origin. In 17 families, we detective promising variants of uncertain significance (VUS) in candidate genes associated with renal development. **b** Circos-style plot of genetic diagnosis in 62 families of ESRD. Ten categories of kidney diseases are indicated outside the widest arc of the circle, chromosome numbers are labeled outside the smaller arc, and gene symbols with patient numbers (patients with pathogenic or likely pathogenic variants) are listed inside. Links are colored by ten categories. **c** From clinical diagnosis to genetic diagnosis for renal disease based on ES study in 115 families. Left and middle: division of the priori clinical diagnosis and change in final diagnosis. Middle and right: division of change in final diagnosis and genetic findings (pathogenic, likely pathogenic variants, and VUS). The width of the lines in the Sankey plot is proportional to the relative quantity of families within each group. ADTKD autosomal dominant tubulointerstitial kidney disease, CAKUT congenital anomalies of the kidney and urinary tract, ESRD end-stage renal disease, GN glomerulonephritis, HSPN Henoch–Schonlein purpura nephritis, SRNS steroid-resistant nephrotic syndrome, TIKD tubulointerstitial kidney disease, NPHP nephronophthisis.

We performed exome sequencing (ES) in 421 individuals from the 115 families enrolled in this study. Besides 115 probands for genetic diagnosis, 111 affected individuals with CKD as family members received genetic detection, and 195 health family members underwent genetic screening for potential living kidney donors. A molecular genetic diagnosis was identified in 53.9% (62/115) of familes with suspected genetic kidney disease on the transplant waitlist. Of these families, 13 (11.3%) had an autosomal recessive (AR) disease, 18 (15.7%) had an autosomal dominant (AD) disease, and 31 (27.0%) had an X-linked dominant (XLD) disease. Seventeen different monogenic causes of kidney disease were confirmed in the families with a primary clinical diagnosis of GN (28), SRNS (27), CAKUT (3), TIKD (2), and ESRDu (2). ES confirmed a specific underlying cause within the broader category of clinical suspected disease in 33 (28.7%) families, modified the diagnosis in 24 (20.9%) families, reclassified the primary disease in three families, and establish a diagnosis in two families with ESRDu. (Fig. 1, Table 1, and Supplementary Table 2)

**Table 1:** Information on a priori clinical diagnosis and post genetic diagnosis that reclassifies the primary diagnosis in 29 families.

Family ID	A *priori* clinical Dx	Age at initial Dx, gender of probands	Initial clinical features	Renal ultrasound	Renal pathological findings	Extrarenal manifestations	Age at developing into ESRD	Genotype (inheritance)	c.Change^a^; p.Change^b^; zygosity, segregation (p,m,s)	gnomAD^c^ (all; EA)	HGMD^d^; ACMG^e^ category	Post ES Dx
fam10024901	ESRDu	22 yrs., M	Non-nephrotic proteinuria, hematuria	Hyperechogenicity of bilateral kidney	N.D.	None	54 yrs.	***PAX2*** (AD)	NM_003990.4: c.239C>A; p.Pro80Gln (het, *de novo*)	None	N, LP (PM1, PM2, PM5, PP3)	FSGS
100059	GN	100059_21: 35 yrs., F; 100059_22: 23 yrs., F; 100059_23: 34 yrs., F	Nephrotic proteinuria	Diffuse lesions of both kidneys, reduced size of both kidneys	N.D.	Both hearing lost	100059_21: 37 yrs.; 100059_22: 41 yrs.; 100059_23: 40 yrs.	***PAX2*** (AD)	NM_003990.4:c.148C>T:p.Arg50Trp (het;m,wt;Sibling_22,het; Sibling_23,het)	None	N, LP (PM1, PM2, PP2, PP3)	FSGS
100062	GN	10 yrs., F	100062_21: nephrotic proteinuria	Diffuse lesions of both kidneys, reduced size of both kidneys	N.D.	None	13 yrs.	***PAX2*** (AD)	NM_003990.4:c.221_226dupAGACCG;p.Glu74_Thr75dup (het;p,wt;m,wt)	None	DM, LP (PM1, PM2, PM4, PP3)	FSGS
100122	TIKD	1000122-21: 27 yrs., M; 10022_11, 37 yrs., M; 10022_11s (paternal sister): 35 yrs., F	No abnormal findings of uranalysis	No abnormal	1000122_21: tubulointerstitis; 10022_11s (paternal sister): sclerosing glomerulonephritis	None	1000122_21: 29 yrs.; 10022_11, 54 yrs.; 10022_11s (paternal sister): 51 yrs.	***PAX2*** (AD)	NM_003990.4:c.1127A>C;p.Gln376Pro (het;p,het;m,wt; paternal sister, het)	0.0001 (48/0/280814); 0.0023 (47/0/19896)	N, LP (PM1, PP1, PP2, PP3, PP4)	Renal coloboma syndrome
100109	GN	13 yrs., F	Non-nephrotic proteinuria, hypercholesterolemia	Reduced size of both kidneys	Tubulointerstitial inflammation with tubular cystic dilatation	None	19 yrs.	***TTC21B*** (AR)	NM_024753.5:c.3664C>T;p.Arg1222Trp(het;p,het;m,wt); c.256A>C:p.Asn86His (het;p,wt;m,het)	None, none; 0.0001 (29/0/282644), 0.0014 (29/0/19952)	N, LP (PM2, PP1, PP3, PP4, PP5); N, LP (PM2, PM3, PP1, PP3, PP4, PP5)	FSGS
100123	GN	100123_21: 25 yrs., F; 100123_31: 6 yrs., M	100123-21, nephrotic proteinuria, hematuria; 100123-31 (proband’s son), non-nephrotic proteinuria		100123_21: lipid deposition in renal tubular and glomerular	None	100123_21: 27 yrs.; 100123_31: CKD 1	***APOE*** (AD)	NM_000041.4:c.127C>T;p.Arg43Cys (het; proband’s son, het)	0.0000 (2/0/250366);0.0000 (1/0/18364)	DM, LP (PM2, PP1, PP2, PP3, PP4)	Lipoprotein nephropathy
100127	CAKUT	100127_21: 41 yrs., F; 100127_22:56 yrs., M; 100127_23: 41 yrs., F	No abnormal findings of urinalysis	100127_21: reduced size of both kidneys, hyperechogenicity of bilateral kidney	N.D.	None	100127_21: 52 yrs.; 100127_22: 60 yrs.; 100127_23: 54 yrs.	***UMOD*** (AD)	NM_001008389.3:c.203A>T;p.Glu68Val(het;m,wt;s22,het;s23,het;s24,wt;s25,wt)	None	N, LP (PM1, PM2, PP2, PP3)	UMOD- ADTKD-
100150	SRNS	100150_21: 32 yrs., M; 100150_13 (paternal uncle): 21 yrs., M	100150_21: nephrotic proteinuria; 100150_13 (paternal uncle): nephrotic proteinuria	100150_21: hyperechogenicity of bilateral kidney with normal size	100150_21: FSGS	100150_13: vision loss	100150_21:38 yrs.; 100150_13(paternal uncle): 53 yrs.	***UMOD*** (AD)	NM_001008389.3: c.1196A>G;p.His399Arg (het; p,wt;m,het)	None	N, LP (PM1, PM2, PP2, PP3)	FSGS
100041	GN	100041_21: 14 yrs., M; 100041_22: 7 yrs., M	Non-nephrotic proteinuria, hematuria, hyperuricemia	Diffuse lesions of both kidneys, reduced size of both kidneys	100041_21:N.D.; 100042_22: chronic tubulointerstitial inflammation	100041_21: hearing lost	100041_21: 16 yrs.; 100041_22: CKD4	***RMND1*** (AR)	NM_017909.4:c.859A>T, p.Ile287Phe (HOM;p,het; m,het;sibling,HOM)	None	N, LP (PM2, PM3, PP1, PP3, PP4)	Mitochondrial disorder
100042	ESRDu	100042_21: 21 yrs., F; 100042_22: 24 yrs., F;	Non-nephrotic proteinuria, hematuria	Diffuse lesions of both kidneys, reduced size of both kidneys	N.D.	None	100042_21:29 yrs.; 100042_22: 32 yrs.	***NPHP3*** (AR)	NM_153240.5:c.3757C>G;p.Leu1253Val (HOM;p,het;m,het;sibling_22, HOM; sibling_23,het)	0.0000 (7/0/251268); 0.0001 (3/0/18394)	DM, LP (PM1, PM2, PP1, PP3, PP4)	NPHP
100054	GN	100054_21: 10 yrs., M; 100054_22: 7 yrs., M	Non-nephrotic proteinuria, hematuria	Diffuse lesions of both kidneys, reduced size of both kidneys	Chronic glomerulopathy with typical Gb3 deposition	Both hearing lost	100054_21: 48 yrs., M;100054_22:41 yrs.	***GLA*** (XL)	NM_000169.2:c.878C>T:p.Pro293Leu (hemi;p,wt;m,het; sibling_22,hemi; sibling_23,wt)	None	DM, LP (PM1, PM2, PP2, PP3, PP4)	Fabry disease
10073	GN	25 yrs., M	Nephrotic proteinuria	Reduced size of both kidneysno abnormal	N.D.	None	27 yrs.	***WT1*** (AD)	NM_024426.6: c.1534C>T,p.Gln512X (het; p,wt)	None	N, LP (PVS1, PM2, PP3)	FSGS
100004	SRNS	14 yrs., M	Nephrotic proteinuria	No abnormal	FSGS	hearing loss	18 yrs.	***COL4A5*** (XL)	NM_000495.4:c.4944G>A:p.W1648X (Hemi; p,wt;m,het;sibling,hemi)	None	N, P (PVS1, PM1, PM2, PP3)	Alport syndrome
100074	SRNS	6 yrs., M	Nephrotic proteinuria	No abnormal	FSGS	hearing loss	17 yrs.	***COL4A5*** (XL)	NM_000495.4:c.2288G>A;p.G763E (hemi;p,wt)	None	DM, LP (PM1, PM2, PP2, PP3)	Alport syndrome
100092	SRNS	24 yrs., M	Nephrotic proteinuria	No abnormal	N.D.	hearing loss	37 yrs.	***COL4A5*** (XL)	NM_000495.4:c.439-1G>A (hemi;p,wt;m,het)	None	DM, P (PVS1, PM2, PP3)	Alport syndrome
100098	SRNS	4 yrs., M	Nephrotic proteinuria	No abnormal	N.D.	None	20 yrs.	***COL4A5*** (XL)	NM_000495.4:c.600_603dupGGGA;p.Phe202Glyfs*2 (hemi;t;p,het;m,wt)	None	N, P (PVS1, PM1, PM2, PP3)	Alport syndrome
100112	SRNS	13 yrs., M	Nephrotic proteinuria	No abnormal	N.D.	None	27 yrs.	***COL4A5*** (XL)	NM_000495.4:c.584G>A;p.Gly195Asp (hemi;p,wt;m,het)	None	Y, P (PM1, PM2, PM5, PP2, PP3)	Alport syndrome
100113	SRNS	100113_21: 7 yrs., M; 100113_22: 2 yrs., M	Nephrotic proteinuria	No abnormal	N.D.	None	100113_21, 17 yrs., M; 100113_22, CKD 1	***COL4A5*** (XL)	NM_000495.4:c.2237G>A;p.Gly746Glu (hemi;p,wt;m,het)	None	DM, LP (PM1, PM2, PP2, PP3)	Alport syndrome
100115	SRNS	32 yrs., M	Nephrotic proteinuria	No abnormal	N.D.	Hearing loss, vision loss	38 yrs.	***COL4A5*** (XL)	NM_000495.4:c.3659G>A;p.Gly1220Asp (het;p,wt;m,het)	None	DM, LP (PM1, PM2, PM5, PP2, PP3)	Alport syndrome
100128	SRNS	4 yrs., M	Nephrotic proteinuria	No abnormal	N.D.	Hearing loss, vision loss	28 yrs.	***COL4A5*** (XL)	NM_000495.4:c.2597G>A;p.Gly866Glu (hemi;p,wt;m,het)	None	DM, LP (PM1, PM2, PP2, PP3)	Alport syndrome
100130	SRNS	22 yrs., M	Nephrotic proteinuria	No abnormal	N.D.	Hearing loss	42 yrs.	***COL4A5*** (XL)	NM_000495.4:c.4960_4961insAAAA;p.Val1654Glufs*8 (hemi;p,wt;m,het)	None	N, P (PVS1, PM1, PM2)	Alport syndrome
100152	SRNS	27 yrs., M	Nephrotic proteinuria	No abnormal	N.D.	None	28 yrs.	***COL4A5*** (XL)	NM_000495.4:c.1871G>T;p.Gly624Val (hemi;p,wt;m,het)	None	N, LP (PM1, PM2, PM5, PP2, PP3)	Alport syndrome
100243	SRNS	3 yrs., M	Nephrotic proteinuria	No abnormal	N.D.	None	20 yrs.	***COL4A5*** (XL)	NM_000495.4:c.670G>A;p.Gly224Arg (hemi;p,hemi;m,wt)	None	N, LP (PM1, PM2, PP1, PP3)	Alport syndrome
100244	SRNS	3 yrs., M	Nephrotic proteinuria	No abnormal	N.D.	None	15 yrs.	***COL4A5*** (XL)	NM_000495.4:c.2395+3A>G (hemi;p,wt;m,het)	None	DM, P (PVS1, PM2, PP1, PP4)	Alport syndrome
100245	SRNS	23 yrs., M	Nephrotic proteinuria	No abnormal	N.D.	None	26 yrs.	***COL4A5*** *(XL)*	NM_000495.4:c.687+1G>A (hemi;m,het)	None	DM, LP (PVS1, PM2, PP3)	Alport syndrome
100235	SRNS	27 yrs., M	Nephrotic proteinuria	No abnormal	N.D.	Vision loss	37 yrs.	***COL4A3*** (AR)	NM_000091.4:c.2990G>A;p.Gly997Glu (HOM;p,het;m,het)	None	DM, LP (PM1, PM2, PM3, PP1, PP3)	Alport syndrome
100240	SRNS	19 yrs., F	Nephrotic proteinuria	No abnormal	N.D.	Vision loss	22 yrs.	***COL4A3*** (AR)	NM_000091.4:c.1855G>A;p.Gly619Arg (het;p,wt;m,het);c.4793T>G:p.Leu1598Arg(het;p,het;m,wt)	None	DM, LP (PM1, PM2, PP2, PP3) DM, LP (PM1, PP1, PP3, PP4, PP5)	Alport syndrome
100131	SRNS	22 yrs., M	Nephrotic proteinuria	No abnormal	N.D.	Hearing loss	24 yrs.	***COL4A3*** (AR)	NM_000091.4:c.1865G>A;p.Gly622Glu (het;p,het;m,wt; sibling,het); c.3575G>A:p.Gly1192Glu (het;p,wt;m,het;sibling,het)	None	N, LP (PM1, PM2, PP2, PP3) DM, LP (PM1, PM2, PP2, PP3	Alport syndrome
100133	SRNS	24 yrs., M	Nephrotic proteinuria	No abnormal	N.D.	None	29 yrs.	***COL4A4*** (AR)	NM_000092.4:c.2726G>A;p.Gly909Glu (het;p,het;m,wt); c.1459+5G>A (het;p,wt;m,het)	None	DM, LP (PM1, PM2, PP2, PP3); N, P (PVS1, PM2, PP1, PP3)	Alport syndrome

*AD* autosomal dominant, *AR* autosomal recessive, *c. change* nucleotide change, *CAKUT* congenital anomalies of the kidney and urinary tract, *CKD* chronic kidney disease, *com* compound, *del* deletion, *DM* disease mutation, *Dx* diagnosis, *ESRD* end-stage renal disease, *ESRDu* ESRD of unknown etiology, *F* female, *fs* frameshift mutation, *FSGS* focal segmental glomerulosclerosis, *GN* glomerulosclerosis, *hemi* hemizygous, *het* heterozygous, *hom* homozygous, *M* male, *m* maternal, *N.D*. not done, *NPHP* nephronophthisis, *p. change* amino acid change, *P*. pathogenic, *SRNS* steroid-resistant nephrotic syndrome, *TIKD* tubulointerstitial kidney disease, *VUS* variants of uncertain significance, *WES* whole exome sequencing, *wt* wild type, *XL* X-linked, *yrs.* years.

^a^Impact of variant on cDNA level.

^b^Impact of variant on the amino acid or protein level.

^c^gnomAD, variant frequencies listed for homozygous/hemizygous (if applicable)/heterozygous/total alleles (http://gnomad.broadinstitute.org/). All, all population, EA, eastern Asian.

^d^HGMD, Human Gene Mutation Database (https://portal.biobaseinternational.com/hgmd). If the exact variant has been reported previously on HGMD^®^ Professional 2020.2 for the reported phenotype and classified as a disease-causing pathogenic mutation, the variant is denoted as “DM.” The variant is denoted as “LD” if the variant is likely a disease-causing pathogenic mutation, but either the author indicated some doubt or subsequent evidence calls the deleterious nature of the variant into question. If the gene, but not the exact variant, has been reported for the corresponding phenotype, then “N” is indicated in this column.

^e^ACMG, American College of Medical Genetics and Genomics Standards and Guidelines Classification as pathogenic, likely pathogenic or VUS (Richards Genet Med 17(5):405, 2015).

The genetic study confirmed the molecular diagnosis for patients’ underdiagnosis kidney disease. First, genetic diagnosis was confirmed for the 22 families whose clinical suspicion of Alport syndrome was lack of the pathological evidence of collagen IV deficiency. Pathogenic variants in *COL4A5* (19, XLD) and *COL4A3* (AR,2; AD 1) were identified respectively in the 22/49 families from GN group without performing the renal histological study of collagen or glomerular basement membrane (GBM). Second, pathogenic variants of known genes were identified for FSGS in 12 families from GN/SRNS group apart from Alport syndrome. The genetic causes of FSGS were shown including *COQ8B* (AR, 3), *TRPC6* (AD, 2), *PAX2* (AD,3), *NPHS2* (AR,1), *NUP160* (AR,1), *WT1* (AD, 1) and *UMOD* (AD, 1). In four families, we detected pathogenic variants in *UMOD* and *PAX2* as the phenocopy genes for FSGS separately. The affected individuals from these families were primary diagnosed of SRNS without abnormal findings of ultrasound. Three of the 91 families referred with GN or FSGS were confirmed the genetic diagnosis of *PAX2*-associated FSGS post multidisciplinary board discussion. All the patients from the three families presented with proteinuria during adolescence. They did not have extrarenal nor syndromic features, renal dysplasia, or histological features of CAKUT. The *PAX2* variant co-segregated across all affected individuals available to us in two families, whereas one *de novo* variant was found in the third family.

Furthermore, the genetic findings modified the final diagnosis (Table 1). Out of the SRNS group without any abnormal findings of in GBM, pathogenic variants in *COL4A5* (XLD, 13), *COL4A3* (AR, 3), or *COL4A4* (AR, 1) were identified in the 17/42 families. We genetically diagnosed the rare diseases including lipoprotein nephropathy with *APOE* variant (AD, 2), nephronophthisis (NPHP, AR, 2), Fabry disease with *GLA* variant (XLR,1), renal coloboma syndrome (AD, 2), and mitochondrial disease (1). After an multidisciplinary team (MDT) discussion, genetic diagnosis was confirmed in two families with lipoprotein nephropathy, one family with Fabry disease, one with mitochondrial disease, one with NPHP, and one with ADTKD. Pathogenic variants in *APOE*-Kyoto(p.Arg25Cys) and *APOE*-Chicago (p.Arg147Pro) were identified in two families. Subsequently, re-staining slides of the kidney biopsy confirmed the histochemical diagnosis of lipoprotein nephropathy. A pathogenic variant of *GLA* for Fabry disease was diagnosed in two affected individuals from one family who presented non-nephrotic proteinuria during adolescence and developed ESRD in their 50s. Rechecking the electron microscope images found the Myelin-like bodies as the typical pathologic features in Fabry disease. In another family with affected siblings, we found biallelic variants in *RMND1* (*trans*) that have been reported in rare cases of mitochondrial disease with renal defects^15^. The two siblings presented initially with hyperuricemia, non-nephrotic proteinuria, and progressed into renal dysfunction. Hearing impairment and tubulointerstitial nephritis shown by renal biopsy were reported in one of them. The older brother had a successful renal transplant at the age of 18. Reverse phenocoping allowed us to correct the diagnosis of primary disease in 5.2%(6/115) families through clinical reassessment and unexpected genetic findings in *PAX2*, *UMOD* and *TTC21B*. Among the eight families with CAKUT, we detected pathogenic variants in known disease-causing genes in three families. A heterogenic variant of isolated CAKUT genes (*ITGB4*, 1) was detected in one CAKUT family. The biallelic variants in NPHP genes (*TTC21B*, 1) were detected in the second CAKUT family. And a heterogenic variant of *UMOD* was confirmed in the third CAKUT family with the final diagnosis of hyperuricemic nephropathy. Out of the 12 families of ESRDu, we detected the biallelic variants of *NPHP3* in one family and the heterogeneous variant in *PAX2* in the second family confirming the diagnosis of renal coloboma syndrome.

### Variants of uncertain significance and secondary findings

In 5.2% of the families (6 of 115), we detected variants of uncertain significance (VUS) in a gene known to cause kidney disease including *LAMA5* (4), *NPHS1* (1), and *TTC21B* (1). The variants did not meet our criteria for definite confirmation of pathogenicity according to American College of Medical Genetics and Genomics (ACMG) guideline^16^. We also detected the VUS based on the candidate gene list associated with renal development in 9.6% of families (Supplementary Table 3). Phenotype–genotype correlation and co-segregation had been analyzed in candidate genes, including *DSCAM, MAZ, KDM2B, VAV2, GLI2, GLI3*, *PLXNB2, LAMP2*, and *SHROOM3*. Variants of *LAMP2* were identified in the male twins who presented with proteinuria and hypertension during adolescence. Both had mild cardiomyopathy, but reported no cardiac symptoms. Danon disease^17^ was suspected for this family.

Although our focus was the genetic diagnosis on primary disease, we also examine the genes recommended by the ACMG published guidelines for secondary findings/incidental findings (Supplementary Table 4). Ten variants of oncogenes were reported as potentially medically actionable and appropriate for return in 4.4% of patients. Further genetic counseling was performed for the two families evaluating and discussing the potential risks for individuals with *BRCA2* pathogenic variants. In addition, polymorphisms variants as genetic determinants for tacrolimus or mycophenolate pharmacokinetics were screened in the 226 patients by ES (Supplementary Table 5 and Supplementary Fig. 6). It provided genetic information to optimize the personalized immunosuppressant dosage in kidney transplantation.

## Discussion

In this study, we show the potential diagnostic role of ES in adult patients with familial kidney disease ready for renal transplantation. ES of parent–child trios provided a molecular genetic diagnosis for 53.9% families with suspected genetic kidney disease on the waitlist for transplantation.

Familial testing through ES has improved the diagnostic accuracy in patients with CKD, especially in patients with familial undetermined kidney disease. Patients with genetic disorders can develop ESRD and receive transplantation without a correct diagnosis of causal nephropathy, and that these disorders can cluster and reach a higher-than-expected prevalence in this setting. It has been shown to have a higher diagnostic yield in patients with a positive family history of CKD, patients with extrarenal manifestations, or patients with early-onset age^12,13,18^. Targeted ES in a broad CKD population identified diagnostic variants in 307 of 3315 (9.3%) adult patients including 64.7% of the patients with ESRD^12^. Genetic diagnosis was identified in 37% (50/153) of the adult patients with undetermined ESRD through a kidney-specific gene panel^9^. Here we reported a molecular genetic diagnosis was confirmed in 54% of the 115 families with suspected genetic kidney disease. The dissimilarities in diagnostic yield between these studies likely result from differences in the sample size, inclusion criteria, sequencing technique approach, and different selection of genes. It is crucial to identify the genetic diagnosis and to access the potential risks for recipients and donors for kidney transplantation. Therefore, we recruited the patients from the waitlist after accessing the need for genetic analysis for kidney disease suspected to have an inherited basis in the setting of multiple affected family members, extrarenal phenotype, or early-onset age. We excluded the AD polycystic kidney disease (ADPKD) patients with a clear family history in which modified PCR conditions were efficient for sequencing of the *PKD1* gene. The excluded patients with ADPKD accounted for 4.5% of our waitlist for transplantation.

The diagnostic utility of the genetic findings provided new clinical insight in most families that helps to preplanned renal transplantation. A high index of suspicion is needed to detect the key phenotype that may be hidden in patient history suggesting the presence of genetic kidney disease. Reverse phenotyping following ES among patients clinically diagnosed based on nonspecific findings of renal histology or radiology could rescue wrong diagnosis to around 30% in kidney disease^19^. Here we corrected the diagnosis of the primary disease in 5% of the 115 families through reverse phenotyping. The missing diagnosis can have a serious impact on graft survival and in general on management transplant patients and on other affected family members. A timely diagnosis of certain rare disease, such as Fabry disease and mitochondrial disease identified in our study, is paramount for patient care. As is known enzyme replacement therapy is considered safe after kidney transplantation, and protective in terms of graft and patient survival, continuing even after the transplant to carry out a protective action on the extrarenal aspects of the disease.

It is necessary for appropriate donor selection for transplantation and surveillance of at-risk family members^9^. A significant proportion of ESRD patients who subsequently received transplantation have a presumptive diagnosis of nephropathy that turns out to be wrong. The increased risk of ESRD post donation in related living donors may reflect a missed genetic disease. Of the 17 monogenic disorders detected in our study, Alport syndrome due to *COL4A5*/*COL4A3* accounted for one third of all genetic diagnoses. These patients were underdiagnosed because of a lack of the pathological evidence of collagen IV deficiency or abnormal findings by electron microscopy. The genetic implications are different for the affected individuals and other family members with X-linked or AR Alport syndrome. All disorders arising from abnormalities of the collagen IV α345 molecule as forms of Alport syndrome can present with highly variable phenotypes. Selection of living related donors for patients with Alport syndrome requires careful consideration of the risk for CKD on the basis of the genotype^20^. In total, we confirmed the genetic diagnosis in 15.7% of families with AD inherited disease, and 27% with XLD inherited disease. Owing to incomplete penetrance in AD inherited disease, not all individuals with pathogenic variants will be symptomatic. AD causes of FSGS often manifest later in life and can be associated with variable expressivity. Additional clinical evaluation was carried out for the potential donors with pathogenic variants considering the frequency of secondary genetic findings in the general population^5^.The family members who carried a variant in one of those genes should be preclude from living kidney donation, given their risks for developing disease. Hence, genetic screening should be offered for all at-risk living donors.

A genetic diagnosis may also validate a risk estimation of post-transplant recurrence. Genetic FSGS, except for *NPHS1*-nepropathy, is considered to have a low risk of recurrence in the post-transplant setting. We reported the modification of diagnosis in 30% families, including 14 families from primary diagnosis of FSGS to Alport syndrome. We also demonstrated pathogenic variants in the phenocopy genes such as *UMOD*^21^*, TTC21B*^22^, or *PAX2*^23^ associated FSGS. The genetic subtype of FSGS established collectively accounted for 10% (12/115) of the genetic causes in our cohort. Furthermore, although allograft survival is improved after living donation when compared with deceased donation, there may be a lot of hesitance in pursuing living donor transplantation in patients with FSGS because of recurrent disease. There is a high risk of post-transplant disease reoccurrence in the idiopathic group that is postulated to be caused by circulating factors other than monogenetic cause. Among the 39.6% (36/91) of the families referred of GN or SRNS in our study, pathogenic variants were absent that may be an alarm for high risk of post-transplant recurrence.

The disclosure of the VUS of candidate disease-causative genes may help to identify much of the missing pathogenicity for renal disease. VUS should generally only be considered for reporting where there is a high level of supporting evidence^24^. In this study we reported the VUS of known causative genes for kidney disease in 5.2% of the families and VUS in candidate genes associated with renal development in 9.6% of the families. Solving the problem of VUS interpretation relies on further functional study on the candidate genes. As deficient mouse models of these candidate genes (such as *GLI2*^25^, *GLI3*^26^, *PLXNB2*^27^, and *SHROOM3*^28^) have shown phenotypes of renal disorders, the promising VUS could provide much information for research teams.

We reported, for the first time, the secondary findings following ES for ESRD patients. The germline variants of oncogenes in 4.4% of the 115 patients preparing for transplant should be interpreted more carefully. It was reported that 4.8% (7/145) of ES screening referrals for a variety of rare genetic diseases had secondary findings related to cancer^29^. Kidney transplant recipients are at least two times more likely to develop or die of cancer than the general population^18,30^. Transplant candidates and potential donors should be screened for cancer as part of the assessment process^30^. Secondary findings of cancer predisposing genes may provide more evidence to tailor cancer screening in transplant candidates. In addition, the disclosure of secondary findings on pharmacogenetics is of great significance for transplantation^31^. Attempts have been made to create dosing models that based on these genetic factors as well as clinical factors to predict the dosage of tacrolimus or mycophenolate^32^. Genetic findings on pharmacogenetics through ES may contribute to the preplanning for transplantation.

The limitations of our study included a modest cohort size of relative ethnic homogeneity. There may be a selection of patients with stable situations enrolled in the transplantation center. According to the geographic distribution of the 64 dialysis centers in 18 provinces, we suspected that our findings are relevant to the other parts of China. Most families (90.4%) of our study had multiple effects, resulting in three-quarters of them with a genetic diagnosis of AD or XL disease. Some of the families who have pathogenic variants of incomplete penetrance could had been missed in our study. Such as complement genes contribute to atypical hemolytic uremic syndrome (aHUS) were absent in our study. aHUS requires perioperative and lifelong complement blockage to prevent post-transplant recurrence. The high diagnostic rate on ES clearly highlights the potential medical-economic savings to patients and insurance companies paying for testing. Further genetic screening should be conducted for all the ESRD cases without secondary causes before transplant. Second, deep intronic variants, variants within variable number tandem repeats, copy number variations, and missing sequence omitted to exome capture kit (i.e., *GREB1L, PKD1*) would have been missed by our approach. Our study probably underestimates the overall burden of genetic disorders among patients with ESRD. Genome sequencing is an increasingly important comprehensive method with which to investigate the genetic causes of inherited renal disease. Furthermore, the inability to fully interpret all the variants limits the use of sequencing data for both the patients and their family members. Improved methods in which variants are interpreted in concert with clinical diagnoses may address this deficiency.

Overall, our study demonstrates that ES reveals the underlying genetic causes in a significant proportion of adult patients with suspected genetic kidney disease ready for transplantation. The genetic findings allow for improving the diagnosis of primary disease that helps to assess the risk of post-transplant recurrence in the affected individuals and to surveil all at-risk live donors. It may promote the strategy of optimizing transplantation management in the future. More genetic work and long-term follow-up in a larger cohort could fully evaluate the benefit of genetic analysis before renal transplantation.

## Methods

### Study design and participants

Renal transplant candidates referred to the Organ Transplant Center at the First Affiliated Hospital of Zhengzhou University were recruited perspectively between January 2017 and December 2019. The patients were referred for the evaluation and management of kidney disease and consented to a general genetic research program. Written informed consent offered the possibility to opt yes or no for disclosure of secondary findings, unrelated to the referral condition. Patients could choose if they wished to have their samples and/or data for future research, both anonymously or not. This study is compliant with the “Guidance of the Ministry of Science and Technology (MOST) for the Review and Approval of Human Genetic Resources,” which requires formal approval for the export of human genetic material or data from China. This study was approved and monitored by the Institutional Review Board of the First Affiliated Hospital of Zhengzhou University (No. 2017-KY-106).

Our recruitment process is summarized in Fig. 1a. The eligibility criteria included the following: (i) age at registry on the waiting list of kidney transplantation more than 18 years; (ii) a family history of kidney disease that was defined as any family members with urinary abnormalities or impaired kidney function or undiagnosed kidney disease; or clinical suspicion of a genetic kidney disease owing to age onset less than 25 years or extrarenal features^8,9,11^. These families were referred for the evaluation and management of kidney disease and consented to a general genetic research. The following exclusion criteria were listed: (i) ADPKD that diagnosis based on radiological study and long-range PCR sequencing rather than ES; (ii) specific histological renal diagnosis (e.g., Alport syndrome diagnosed with collagen IV a3/a4/a5 abnormal pathological findings); and (iii) specific and plausible clinical diagnosis (e.g., long-term history of diabetes mellitus before ESRD, systemic lupus erythematosus, acquired obstructive uropathy, tumor, etc.).

The primary clinical diagnosis of each patient was determined via medical history review and the primary nephrologist’s referral into one of the following a *priori* clinical diagnosis categories^33^:

- GN: encompassing membranoproliferative GN, crescentic GN, and hemolytic uremic syndrome.
- SRNS, or nephrotic syndrome with biopsy findings of FSGS.
- CAKUT, defined as any abnormality of number, size, shape, or anatomic position within the kidneys or urinary tract.
- Cystic kidney disease including nephronophthisis (NPHP), medullary cystic disease, and other renal cystic ciliopathies.
- TIKD: with biopsy findings of chronic tubulointerstitial nephritis without an obvious precipitating cause.
- ESRD of unknown etiology (ESRDu): patients developed into ESRD with no information of renal histology or definite radiological diagnosis, or no other clinically plausible cause, for instance Fabry disease.

### Exome sequencing and variant information

The samples of the affected individuals were subjected to ES of parent–child trios after the informed consent was obtained. Some of their unaffected family members as potential living donors were selected for ES post informed consent. ES procedure and variants annotation have been described in detail previously^12^. Genomic DNA was isolated from blood lymphocytes and was fragmented to an average size of 250 bp. End repair, adapter ligation, and PCR enrichment were performed following the protocol for VAHTS TM Universal DNA Library Prep Kit for Illumina V3 (Vazyme Biotech Co., Ltd, Nanjing, China). The enriched DNA libraries were subjected to exome capture using Agilent SureSelect Clinical Research Exome V2 or Human All Exon V7. The resulting libraries were sequenced on Illumina sequencers (HiSeq 4000 or Hiseq X) with the paired-end of 150 bp at Precision Medicine Center of Zhengzhou University. Variant interpretation was performed manually by a panel of nephrologists and clinical molecular geneticists. For clinical sequence interpretation the variants were classified according to the ACMG guidelines^16^.

### Primary findings for the monogenic form of nephropathy

In brief, we prioritized variants that occurred in a known CKD-related gene panel^7,9,12^ (Supplementary Table 7) depending on the a *priori* clinical diagnosis and phenotype information. Diagnostic variants were defined as “pathogenic” or “likely pathogenic” according ACMG guidelines. For patients referred with a clinical diagnosis of nephrotic syndrome, we manually searched for the p.Arg229Gln variant in the *NPHS2* gene, because this allele occurs at a frequency of >1%^34^. All diagnostic variants were confirmed by Sanger sequencing with segregation. Additional clinical evaluation and follow-up were performed for the affected and unaffected relatives with pathogenic variants confirmed, especially for the potential donors.

If the genetic diagnosis remained unsolved for a family, we also evaluated the VUS of known disease-causative genes through discussion combined with the genotype and phenotype. In addition, we analyzed those variants in a virtual renal development gene panel based on all the pathways regulating kidney development as well as the regulators associated with renal transport and metabolism identified in human studies and animal models^18^ (Supplementary Table 8). Variants were manually evaluated for possible pathogenicity by a MDT of clinical geneticists, nephrologists, and pathologists after complete genotype–phenotype comparison.

### Secondary findings

We assessed the sequence data for pathogenic variants in the 59 genes recommended by the ACMG to reveal any medically actionable secondary findings for individuals undergoing ES^35^. Per the ACMG recommendations for the analysis of secondary findings, only variants classified as known pathogenic or expected pathogenic were noted.

To investigate the individual pharmacogenetics in transplantation recipients, we collected the allelic variants with functional effects on the pharmacokinetics of tacrolimus or mycophenolate mofetil from the Pharmacokinetics Knowledgebase^36^. We performed the variant screening in 18 established single-nucleotide polymorphisms (Supplementary Table 4).

### Reporting summary

Further information on variants data and research design is available in the Nature Research Reporting Summery linked to this article.

## Supplementary information

Supplementary Information

Reporting Summary

## Data Availability

The pathogenic variants have been submitted to ClinVar (https://www.ncbi.nlm.nih.gov/clinvar/), and the submission number is SUB9604514. This study is compliant with the “Guidance of the Ministry of Science and Technology (MOST) for the Review and Approval of Human Genetic Resources,” which requires formal approval for the export of human genetic material or data from China. The phenotypic data that support the findings of this study are available from the corresponding author upon reasonable request.
