# Supplementary material for: An accessible insight into genetic findings for transplantation recipients with suspected genetic kidney disease
Source: NPJ Genom Med. 2021 Jul 2;6:57. doi: 10.1038/s41525-021-00219-3 (PMC8253729; doi:10.1038/s41525-021-00219-3)
Supplement: Supplementary file 1 — Supplementary Information [file 41525_2021_219_MOESM1_ESM.pdf]

SUPPLEMENTARY REPORT

An Accessible Insight into Genetic Findings for Transplantation Recipients with suspected genetic kidney disease

#Zhigang WANG1, #Hongen XU2, #Tianchao XIANG3,4, Danhua LIU2,5, Fei XU1, Lixiang ZHAO1, Yonghua FENG1, Linan XU3,4, Jialu LIU3,4, Ye FANG3,4, Huanfei LIU2, Ruijun LI2, Xinxin HU2, Jingyuan GUAN2, Longshan LIU6, Guiwen FENG1, Qian SHEN3,4, HONG XU3,4, Dmitrij Frishman, Wenxue TANG7, *Jiancheng GUO2,5,8, *Jia RAO3,4,9, *Wenjun SHANG1

Supplementary section S1. Supplementary Methods

Supplementary Table S2. 412 known genes that represent monogenic causes of human chronic kidney disease.

Supplementary Table S3. A virtual renal development gene panel.

Supplementary Table S4. Allele frequency of variants associated with drug pharmacogenetics in the 226 patients with CKD from the families on waiting list of transplantation

Supplementary Figure S5. Distribution of the dialysis centers with patients on the wailing list for transplantation.

Supplementary Table S6. Identifying mutations in 62 families of ESRD post WES study.

Supplementary Table S7. A potentially pathogenic mutations in candidate genes identified in 17 families

Supplementary Table S8. Secondary findings with pathogenic and likely pathogenic variants unrelated to the phenotype of the probands based on the recommended minimum list of genes selected by the ACMG committee.

Supplementary Figure S9. Exome sequencing for genetic determinant of associated with tacrolimus or mycophenolate concentration on the waitlist cohort of kidney transplant.

Supplementary section S10. QA/QC reports

Supplementary section S1. Supplementary Methods

Whole-exome sequencing

All patients and their unaffected family members selected underwent WES for diagnosing kidney disease. Genomic DNA was isolated from blood lymphocytes and was fragmented to an average size of 250 bp. End repair, adapter ligation, and PCR enrichment were performed following the protocol for VAHTS TM Universal DNA Library Prep Kit for Illumina V3 (Vazyme Biotech Co., Ltd, Nanjing, China). The enriched DNA libraries were subjected to exome capture using Agilent SureSelect Clinical Research Exome V2 or Human All Exon V7. The resulting libraries were sequenced on Illumina sequencers (HiSeq 4000 or Hiseq X) with the paired-end of 150 bp at Precision Medicine Center of Zhengzhou Univerisity.

Bioinformatics processing

Sequencing adapters and low-quality reads were trimmed from raw reads with Trimmomatic 1. Clean reads were then mapped to the human reference genome assembly (GRCh37) using Burrow-Wheeler Aligner (version 0.7.17-r1188) 2. Single nucleotide variants (SNVs) and small indels were characterized using the HaplotypeCaller from the Genome Analysis Toolkit (version 4, GATK4) 3. Variants were annotated using SnpEff 4 and Vcfanno 5 with a number of databases for predicted effect on protein function, allele frequency in healthy population (1000 Genomes Project database 6, dbSNP 7, Exome Aggregation Consortium ExAC 8, and Genome Aggregation Database gnomAD 9), pathogenicity annotations (ClinVar 10, InterVar 11 and HGMD 12), and for *in silico* pathogenicity predictions in dbNSFP 13, which compiled mutation prediction scores from many algorithms. All analysis steps described above were performed in the framework of bcbio-nextgen (https://github.com/bcbio/bcbio-nextgen).

Key resources used for bioinformatics processing are listed below:

Resource	URL
SnpEff	http://snpeff.sourceforge.net/
Vcfanno	https://github.com/brentp/vcfanno/
1000 Genomes Project	http://www.internationalgenome.org/

dbSNP	https://www.ncbi.nlm.nih.gov/SNP/
ExAC	http://exac.broadinstitute.org/
gnomAD	http://gnomad.broadinstitute.org/
ClinVar	https://www.ncbi.nlm.nih.gov/clinvar/
InterVar	http://wintervar.wglab.org/
HGMD	http://www.hgmd.cf.ac.uk/ac/index.php
dbNSFP	https://sites.google.com/site/jpopgen/dbNSFP

Variant filtering and interpretation

The identified variants with SnpEff annotation effects including intergenic_region, upstream_gene_variant, downstream_gene_variant, 5_prime_UTR_variant, 3_prime_UTR_variant, intron_variant, and non_coding_transcript_exon_variant were further removed. At the same time, variants with ada_score >0.5, or rf_score >0.5, or were annotated as likely pathogenic or pathogenic or VUS in ClinVar database were kept. We then filtered out variants with minor allele frequency >0.05 in any general continental population in which at least 2,000 alleles were observed in the gnomAD database, except those on ACMG benign stand-alone exception list 14.

The resulting variant set was loaded into the GEMINI 15 for exploring variants based on inheritance patterns, including autosomal dominant, *de novo*, autosomal recessive, compound heterozygous, X-linked dominant, and X-linked recessive. We then prioritized variants that occurred in the nephropathy associated genes list (Box 1). Variant interpretation (Box 2) was performed by a panel of an nephrologist with expertise in inherited kidney diseases, a bioinformatician, and a molecular geneticist using the American College of Medical Genetics and Genomics and the Association for Molecular Pathology (ACMG) guidelines for clinical sequence interpretation 16. Diagnostic variants were defined as “pathogenic” or “likely pathogenic” according ACMG guidelines. And we also evaluate the variants of uncertain significance (VUS) of known disease causative genes through combined analysis with genotype and phenotype. All diagnostic variants were confirmed by Sanger sequencing and tested for co-segregation in the family.

Box 1. Variant filtering strategy for identifying the potential pathogenic variants in genes known to cause kidney disease

i. Keep rare variants present with a minor allele frequency (MAF) <1% in healthy control cohorts

dbSNP147 (https://www.ncbi.nlm.nih.gov/projects/SNP).

ii. Keep non-synonymous variants and intronic variants that are located within splice sites.

iii. Applying known gene approach by selecting all variants detected in known kidney disease genes.17

iv. Ranking of remaining variants based on their predicted likelihood to be deleterious for the function of the encoded protein using Polyphen 2

(http://genetics.bwh.harvard.edu/pph2, SIFT (http://sift.jcvi.org/) and Mutation Taster (http://www.mutationtaster.org)

v. Reviewing literature and review with referring physician delineating whether the detected mutation matches the phenotype.

vi. Cross reference with the ACMG guidelines to determine if pathogenic, likely pathogenic or a variant of uncertain signi

Box 2. Variant analysis criteria

Autosomal recessive variants

Disease-causing variants in recessive genes were considered if two alleles were found in the same individual that fulfilled at least one of the following criteria:

i) Truncating allele (stop, abrogation of start or stop, obligatory splice site, or frameshift); OR

ii) Missense mutation if a minimum of 4 of 5 of the following criteria were met:

- Continuously conserved at least among vertebrates (or beyond)
- Previously reported as disease causing or functional evidence implicating causality
- Loss of function in human allele is supported by functional data
- Phenotype correlates with the published phenotype for the gene
- Predicted deleterious for the protein function (at least in two among three prediction programs (Polyphen (>0.5), SIFT (Del.), Mutation taster (D.C.))

Exclude allele as disease causing if:

- Allele frequency >1% (in ExAC, gnomAD, 1000 genomes)
- Non segregation: if compound heterozygous variants are in cis or if an affected family member is without the variant or an unaffected family member is with the variant

Discussion of genotype-phenotype correlation in a panel of nephro-geneticists followed by review of clinical phenotype with referring physician

Autosomal dominant variants

Disease-causing variants in dominant genes were considered if one allele fulfilled at least one of the following criteria:

i) Truncating mutation (stop, abrogation of start or stop, obligatory splice site, and frameshift); OR

ii) Missense mutation if a minimum of 4 of 5 of the following criteria were met:

- Continuously conserved at least among vertebrates (or beyond)
- Previously reported as disease causing or functional evidence implicating causality
- Phenotype correlates with the published phenotype for the gene
- Predicted deleterious for the protein function (at least in two among three prediction programs (Polyphen (>0.5), SIFT (Del.), Mutation taster (D.C.))

Exclusion criteria:

- Allele frequency >0.1% ((in, ExAC, gnomAD, 1000 genomes)
- Non segregation:if the allele did not segregate with the affected status in the family.

Or If an unaffected family member is with the allele consider incomplete penetrance and variable expressivity

Discussion of genotype-phenotype correlation in a panel of nephro-geneticists followed by review of clinical phenotype with referring physician

Quality control of sequencing data

The QC was performed at many stages of the analysis pipeline, including pre-cleaning, post-cleaning, post-alignment, and post-variant-calling. The average sequencing depth of targeted regions is 98.3X, with 95% on average of the targeted based sequenced at least 20 times. Detailed QC results for each WES sample were shown in the supplementary Section S10.

References

1. Bolger AM, Lohse M, Usadel B. Trimmomatic: a flexible trimmer for Illumina sequence data. Bioinformatics 2014;30:2114-20.

2. Li H. Aligning sequence reads, clone sequences and assembly contigs with BWA-MEM. arXiv 2013;1303.3997.

3. DePristo MA, Banks E, Poplin R, et al. A framework for variation discovery and genotyping using next-generation DNA sequencing data. Nature Genetics 2011;43:491-8.

4. Cingolani P, Platts A, Wang le L, et al. A program for annotating and predicting the effects of single nucleotide polymorphisms, SnpEff: SNPs in the genome of Drosophila melanogaster strain w1118; iso-2; iso-3. Fly (Austin) 2012;6:80-92.

5. Pedersen BS, Layer RM, Quinlan AR. Vcfanno: fast, flexible annotation of genetic variants. Genome biology 2016;17:118.

6. Genomes Project C, Auton A, Brooks LD, et al. A global reference for human genetic variation. Nature 2015;526:68-74.

7. Sherry ST, Ward MH, Kholodov M, et al. dbSNP: the NCBI database of genetic variation. Nucleic acids research 2001;29:308-11.

8. Lek M, Karczewski KJ, Minikel EV, et al. Analysis of protein-coding genetic variation in 60,706 humans. Nature 2016;536:285-91.

9. Karczewski KJ, Francioli LC, Tiao G, et al. Variation across 141,456 human exomes and genomes reveals the spectrum of loss-of-function intolerance across human protein-coding genes. bioRxiv 2019:531210.

10. Landrum MJ, Lee JM, Benson M, et al. ClinVar: improving access to variant interpretations and supporting evidence. Nucleic acids research 2018;46:D1062-D7.

11. Li Q, Wang K. InterVar: Clinical Interpretation of Genetic Variants by the 2015 ACMG-AMP Guidelines. American journal of human genetics 2017;100:267-80.

12. Stenson PD, Ball EV, Mort M, Phillips AD, Shaw K, Cooper DN. The Human Gene Mutation Database (HGMD) and its exploitation in the fields of personalized genomics and molecular evolution. Current protocols in bioinformatics 2012;Chapter 1:Unit1 13.

13. Liu X, Wu C, Li C, Boerwinkle E. dbNSFP v3.0: A One-Stop Database of Functional Predictions and Annotations for Human Nonsynonymous and Splice-Site SNVs. Human mutation 2016;37:235-41.

14. Ghosh R, Harrison SM, Rehm HL, Plon SE, Biesecker LG, ClinGen Sequence Variant Interpretation Working G. Updated recommendation for the benign stand-alone ACMG/AMP criterion. Human mutation 2018;39:1525-30.

15. Paila U, Chapman BA, Kirchner R, Quinlan AR. GEMINI: integrative exploration of genetic variation and genome annotations. PLoS computational biology 2013;9:e1003153.

16. Richards S, Aziz N, Bale S, et al. Standards and guidelines for the interpretation of sequence variants: a joint consensus recommendation of the American College of Medical Genetics and Genomics and the Association for Molecular Pathology. Genet Med 2015;17:405-24.

17. Groopman EE, Marasa M, Cameron-Christie S, et al. Diagnostic Utility of Exome Sequencing for Kidney Disease. N Engl J Med 2019;380:142-51.

Supplementary Table S2. 412known genes that represent monogenic causes of human chronic kidney disease

Gene	Protein	Reference	Mode of i	Category
B3GALTL	Beta 3-Glucosyltransferase	Lesnik Oberstein Am J Hum Genet 79:562, 2006	AR	Syndromic CAKUT
BBS6	Bardet-Biedl Syndrome 7	Tieder Int J Pediatr Nephrol 3:199, 1982	AR	Syndromic CAKUT
BBS8	Bardet-Biedl Syndrome 8	Tieder Int J Pediatr Nephrol 3:199, 1982	AR	Syndromic CAKUT
BSCL2	BSCL2, Seipin Lipid Droplet Biogenesis Associated	Haghighi Clin. Genet. 89: 434, 2016	AR	Syndromic CAKUT
CD151	CD151 Molecule (Raph Blood Group)	Karamatic Blood 104:2217, 2004	AR	Syndromic CAKUT
CD96	CD96 Molecule	Kaname AJHG 81:835, 2007	AR	Syndromic CAKUT
CHRNG	Cholinergic Receptor Nicotinic Gamma Subunit	Vogt J Med Genet 49:21, 2012	AR	Syndromic CAKUT
CISD2	CDGSH Iron Sulfur Domain 2	Amr AJHG 81:673, 2007	AR	Syndromic CAKUT
CTU2	Cytosolic Thiouridylase, subunit 2	Shaheen AJMG 170:3222, 2016	AR	Syndromic CAKUT
CYP21	Cytochrome P450 Family 21	Martul Arch Dis Child 55:324, 1980	AR	Syndromic CAKUT
DACH1	Dachshund Family Transcription Factor 1	Schild Nephrol Dial Transplant 28:227, 2013	AR	Syndromic CAKUT
DHCR7	7-Dehydrocholesterol Reductase	Löffler AJHG 13;95:174, 2000	AR	Syndromic CAKUT
EMG1	EMG1, N1-Specific Pseudouridine Methyltransferase	Armistead AJHG 84:728, 2009	AR	Syndromic CAKUT
ERCC8	Excision repair cross-complementing, group 8	Bertola J Hum Genet 51:701, 2006	AR	Syndromic CAKUT
ESCO2	Establishment Of Sister Chromatid Cohesion N-Acetyltransferase 2	Vega J Med Genet 47:30, 2010	AR	Syndromic CAKUT
ETFA	Electron Transfer Flavoprotein Alpha Subunit	Lehnert Eur J Pediatr 139:56, 1982	AR	Syndromic CAKUT
ETFB	Electron Transfer Flavoprotein Beta Subunit	Lehnert Eur J Pediatr 139:56, 1982	AR	Syndromic CAKUT
ETFDH	Electron Transfer Flavoprotein Dehydrogenase	Lehnert Eur J Pediatr 139:56, 1982	AR	Syndromic CAKUT
FANCA	Fanconi Anemia Complementation Group A	Joenje & Patel Nat Rev Genet 2:466, 2001	AR	Syndromic CAKUT
FANCB	Fanconi Anemia Complementation Group B	McCauley Am J Med Genet A 155A:2370, 2011	AR	Syndromic CAKUT
FANCD2	Fanconi Anemia Complementation Group D2	Kalb AJHG 80:895, 2007	AR	Syndromic CAKUT
FANCE	Fanconi Anemia Complementation Group E	Wegner Clin Genet 50:479, 1996	AR	Syndromic CAKUT
FANCI	Fanconi Anemia Complementation Group I	Savage AJMG 170A:386, 2015	AR	Syndromic CAKUT
FANCL	Fanconi Anemia Complementation Group L	Vetro Hum Mutat 36:562, 2015	AR	Syndromic CAKUT
FAT4	FAT Atypical Cadherin 4	Alders Hum Genet 133:1161, 2014	AR	Syndromic CAKUT
FOXP1	Forkhead Box P1	Bekheirnia Genet Med 19:412, 2017	AR	Syndromic CAKUT
HES7	Hes Family BHLH Transcription Factor 7	Sparrow Hum Mol Genet 17:3761, 2008	AR	Syndromic CAKUT
HYLS1	HYLS1, Centriolar And Ciliogenesis Associated	Paetau J Neuropathol Exp Neurol 67:750, 2008	AR	Syndromic CAKUT
ICK	Intertinal cell kinase	Lahiry AJHG 84:822, 2009	AR	Syndromic CAKUT
IFT46	Intraflagellar Transport 46	Lee Dev Biol 400:248, 2015	AR	Syndromic CAKUT
IFT74	Intraflagellar Transport 74	Cevik PLoS GeneT 9:e1003977, 2013	AR	Syndromic CAKUT
JAM3	Junctional Adhesion Molecule 3	Mochida Am J Hum Genet 10;87:882, 2010	AR	Syndromic CAKUT
LFNG	LFNG O-Fucosylpeptide 3-Beta-N-Acetylglucosaminyltransferase	Sparrow Am J Hum Genet 78:28, 2006	AR	Syndromic CAKUT
LMNA	Lamin A/C	Klupa Endocrine 36:518, 2009	AR	Syndromic CAKUT
LRIG2	Leucione rich repeats and immunoglobulin like domains containing prot	Stuart AJHG 92:259, 2013	AR	Syndromic CAKUT
LRP2	LDL Receptor Related Protein 2	Kantarci Nat Genet 39:957, 2007	AR	Syndromic CAKUT
LRP4	LDL Receptor Related Protein 4	Li Am J Hum Genet 86:696, 2010	AR	Syndromic CAKUT
MESP2	Mesoderm Posterior BHLH Transcription Factor 2	George-Abraham Am J Med Genet A 158A:1971, 20	AR	Syndromic CAKUT
MKS3	Meckel Syndrome Type 3 Protein	Baala Am J Hum Genet 80:186, 2007	AR	Syndromic CAKUT
PEX5	Peroxisomal Biogenesis Factor 5	Sundaram Nat Clin Pract Gastroenterol Hepatol 5:4	AR	Syndromic CAKUT
PMM2	Phosphomannomutase 2	Horslen Arch Dis Child 66:1027, 1991	AR	Syndromic CAKUT
POC1A	POC1 centriolar protein	Shaheen AJHG 91:330, 2012	AR	Syndromic CAKUT
PROK2	Prokineticin 2	Madan Mol Genet Metab Rep 12:57, 2017	AR	Syndromic CAKUT
RECQL4	RecQ Like Helicase 4	Siitonen Eur J Hum Genet 17:151, 2009	AR	Syndromic CAKUT
ROR2	Receptor Tyrosine Kinase Like Orphan Receptor 2	Wiens Clin Genet 37:481, 1990	AR	Syndromic CAKUT
RPS19	Ribosomal Protein S19	Hoefele Pediatr Nephrol 25:1255, 2010	AR	Syndromic CAKUT
SCARF2	Scavenger Receptor Class F Member 2	Anastasio Am J Hum Genet 87:553, 2010	AR	Syndromic CAKUT
STRA6	Stimulated By Retinoic Acid 6	Golzio Am J Hum Genet 80:1179, 2007	AR	Syndromic CAKUT
TMCO1	Transmembrane And Coiled-Coil Domains 1	Xin Proc Natl Acad Sci U S A 107:258, 2010	AR	Syndromic CAKUT
TWIST2	Twist Family BHLH Transcription Factor 2	Stevens Am J Med Genet 107:30, 2002	AR	Syndromic CAKUT
UBR1	Ubiquitin Protein Ligase E3 Component N-Recognin 1	Vanlieferinghen Genet Couns 14:105, 2003	AR	Syndromic CAKUT
PEX1	Peroxisomal Biogenesis Factor 1	Crane Hum Mutat 26:167, 2005	AR	Syndromic CAKUT
PIGL	Phosphatidylinositol Glycan Anchor Biosynthesis Class L	Schnur Am J Med Genet 72:24, 1997	AR	Syndromic CAKUT
PIGO	Phosphatidylinositol Glycan Anchor Biosynthesis Class O	Krawitz Am J Hum Genet 91:146, 2012	AR	Syndromic CAKUT
PIGN	Phosphatidylinositol Glycan Anchor Biosynthesis Class N	Ohba Neurogenetics 15:85, 2014	AR	Syndromic CAKUT
PIGT	Phosphatidylinositol Glycan Anchor Biosynthesis Class T	Nakashima Neurogenetics 15:193, 2014	AR	Syndromic CAKUT
PIGV	Phosphatidylinositol Glycan Anchor Biosynthesis Class V	Horn Eur J Hum Genet 22:762, 2014	AR	Syndromic CAKUT
PIGY	Phosphatidylinositol Glycan Anchor Biosynthesis Class Y	Ilkovski Hum Mol Genet 24:6146, 2015	AR	Syndromic CAKUT
PTF1A	Pancreas Specific Transcription Factor, 1a	Gurung Mol Med Rep 12:1579, 2015	AR	Syndromic CAKUT
WFS1	Wolframin ER Transmembrane Glycoprotein	Salih Acta Paediatr Scand 80:567, 1991	AR	Syndromic CAKUT
WNT3	Wnt Family Member 3	Niemann Am J Hum Genet 74:558, 2004	AR	Syndromic CAKUT
ZMPSTE24	Zinc Metallopeptidase STE24	Chen Am J Med Genet A 149A:1550, 2009	AR	Syndromic CAKUT
ACTB	Actin Beta	Rivière Nat Genet 44:440, 2012	AD	Syndromic CAKUT
ACTG1	Actin Gamma 1	Rivière Nat Genet 44:440, 2012	AD	Syndromic CAKUT
AIFM3	Apoptosis Inducing Factor, Mitochondria Associated 3	Lopez-Rivera NEJM 376:742, 2017	AD	Syndromic CAKUT
ATXN10	Ataxin 10	Matsuura Nat Genet 26:191, 2000	AD	Syndromic CAKUT
BICC1	BicC Family RNA Binding Protein 1	Kraus Hum Mutat 33:86, 2012	AD	Syndromic CAKUT
BMP7	Bone Morphogenetic Protein 7	Hwang Kidney Int 85:1429, 2014	AD	Syndromic CAKUT
BRAF	B-Raf Proto-Oncogene, Serine/Threonine Kinase	Sarkozy Hum Mutat 30:695, 2009	AD	Syndromic CAKUT
CDC5L	Cell Division Cycle 5 Like	Groenen Genomics 49:218, 1998	AD	Syndromic CAKUT
CREBBP	CREB Binding Protein	Kanjilal J Med Genet 29:669, 1992	AD	Syndromic CAKUT
DACT1	Dishevelled Binding Antagonist Of Beta Catenin 1	Webb Hum Mutat 38:373, 2017	AD	Syndromic CAKUT
EP300	E1A Binding Protein P300	Roelfsema Am J Hum Genet 76:572, 2005	AD	Syndromic CAKUT
ESRRG	Estrogen Related Receptor Gamma	Harewood PLoS One 5:e12375, 2010	AD	Syndromic CAKUT
FBN1	Fibrillin 1	Tokhmafshan Pediatr Nephrol 32:565, 2017	AD	Syndromic CAKUT
FGFR1	Fibroblast growth factor receptor 1	Farrow AJHG 140A:537, 2006	AD	Syndromic CAKUT
FGFR3	Fibroblast growth factor receptor 3	Rohmann Nat Genet 38:495, 2006	AD	Syndromic CAKUT
FGF10	Fibroblast Growth Factor 10	Milunsky Clin Genet 69:349, 2006; Bamforth Am J M	AD	Syndromic CAKUT
FGF8	Fibroblast Growth Factor 8	Falardeau J Clin Invest 118:2822 2008	AD	Syndromic CAKUT
FGF3	Fibroblast Growth Factor 3	Marquis-Nicholson Sultan Qaboos Univ Med J. 13:8	AD	Syndromic CAKUT
FMN1	Formin 1	Dimitrov J Med Genet 47:569, 2010	AD	Syndromic CAKUT
FOXC1	Forkhead Box C1	LeHeup Eur J Pediatr 154:130, 1995	AD	Syndromic CAKUT
FOXF1	Forkhead Box F1	Hilger Hum Mutat 36:1150, 2015	AD	Syndromic CAKUT
GDF3	Growth Differentiation Factor 3	Karaca Am J Med Genet A 167A:2795, 2015	AD	Syndromic CAKUT
GDNF	Glial cell line derived neurotrophic factor	Pini Prato Medicine (Baltimore) 88:83, 2009	AD	Syndromic CAKUT
GFRA1	GDNF Family Receptor Alpha 1	Chatterjee Hum Genet 131:1725, 2013	AD	Syndromic CAKUT
GLI2	GLI Family Zinc Finger 2	Carmichael J Urol 190:1884, 2013	AD	Syndromic CAKUT
HOXA13	Homeobox A13	Halal Am J Med Genet 30:793, 1998	AD	Syndromic CAKUT
HOXD13	Homeobox D13	Garcia-Barceló Am J Med Genet A146A:3181, 2008	AD	Syndromic CAKUT
JAG1	Jagged 1	Kamath Nat Rev Nephrol 9:409, 2013	AD	Syndromic CAKUT
KAT6B	Lysine Acetyltransferase 6B	Campeau Am J Med Genet 90:282, 2012	AD	Syndromic CAKUT
KCTD1	Potassium Channel Tetramerization Domain Containing 1	Marneros Am J Hum Genet 92:621, 2013	AD	Syndromic CAKUT
KCNH2	Potassium Voltage-Gated Channel Subfamily H Member 2	Caselli Am J Med Genet 146A:1195, 2008	AD	Syndromic CAKUT
KRAS	KRAS Proto-Oncogene, GTPase	Schubbert Nat Gene 38:331, 2006	AD	Syndromic CAKUT
LMX1B	LIM Homeobox Transcription Factor 1 Beta	Dreyer Nat Genet 19:47, 1998	AD	Syndromi SRNS
LPP	LIM Domain Containing Preferred Translocation Partner In Lipoma	Hernández-García Am J Med Genet A 158A:1785, 2	AD	Syndromic CAKUT
MAP2K1	Mitogen-activated protein kinase kinase 1	Schulz Clin Genet 73:62, 2007	AD	Syndromic CAKUT
MAP2K2	Mitogen-activated protein kinase kinase 2	Schulz Clin Genet 73:62, 2007	AD	Syndromic CAKUT
MLL2	KMT2D Myeloid/Lymphoid Or Mixed-Lineage Leukemia Protein 2	Banka Eur J Hum Genet 20:381, 2012	AD	Syndromic CAKUT

Supplementary Table S2 (continued)

Gene	Protein	Reference	Mode of i	Alias	Category
NPHP1	Nephrocystin 1	Hildebrandt Nat Genet 17(2):149, 1997	AR		NPHP
INVS	Inversin	Otto Nat Genet 34(4):413, 2003	AR	NPHP2	NPHP
NPHP3	Nephrocystin 3	Olbrich Nat Genet 34(4):455, 2003	AR		NPHP
NPHP4	Nephronophthisis 4	Otto AJHG 71(5):1161, 2002	AR		NPHP
IQCB1	IQ motif containing B1	Otto Nat Genet 37(3):282, 2005	AR	NPHP5	NPHP
CEP290	Centrosomal protein 290kDa	Sayer Nat Genet 38(6):674, 2006	AR	NPHP6	NPHP
DLG5	Discs Large MAGUK Scaffold Protein 5	Marquez J, Mann N, Arana K, Deniz, et al. J Med Gene	AR/AD	NPHP	NPHP
GLIS2	GLIS Family Zinc Finger 2	Attanasio Nat Genet 39(8):1018, 2007	AR	NPHP7	NPHP
RPGRIP1	RPGRIP1 Like	Arts Nat Genet 39(7):882, 2007	AR	NPHP8	NPHP
NEK8	NIMA (never in mitosis gene a) - related kinase 8	Otto JASN 19(3):587, 2008	AR	NPHP9	NPHP
SDCCAG	Serologically Defined Colon Cancer Antigen 8	Otto Nat Genet 42(10):840, 2010	AR	NPHP10	NPHP
TMEM67	Transmembrane Protein 67	Otto J Med Genet 46(10):663, 2009	AR	NPHP11	NPHP
TTC21B	Tetratricopeptide Repeat Domain 21B	Davis Nat Genet 43(3):189, 2011	AR	NPHP12	NPHP
WDR19	WD repeat domain 19	Bedrup AJHG 89(5):634, 2011	AR	NPHP13	NPHP
ZNF423	Zinc finger protein 423	Chaki Cell 150(3):533, 2012	AR	NPHP14	NPHP
CEP164	Centrosomal protein 164kDa	Chaki Cell 150(3):533, 2012	AR	NPHP15	NPHP
ANKS6	Ankyrin repeat and sterile alpha motif domain containing 6	Hoff Nat Genet 45(8):951, 2013	AR	NPHP16	NPHP
IFT172	Intraflagellar transport 172 homolog, (Chlamydomonas)	Halbritter AJHG 93(5):915, 2013	AR	NPHP17	NPHP
CCDC41	Coiled-coil domain containing 41	Failler AJHG 94(6):905, 2014	AR	CEP83	NPHP
DCDC2	Double-cortin domain containing protein 2	Schueler AJHG 96(1):81, 2015	AR	NPHP19	NPHP
MAPKBP	Mitogen activated protein kinase-binding protein 1	Macia AJHG 100(2):323, 2017	AR	NPHP20	NPHP
ADAMT9	ADAM Metallopeptidase With Thrombospondin Type 1 Motif 9	Am J Hum Genet. 2019 Jan 3;104(1):45-54.	AR		NPHP
IFT81	Intraflagellar Transport 81	Perrault J Med Genet 52(10):657, 2015	AR	CDV-1	NPHP
TRAF3IP	TNF receptor-associated factor 3-interacting protein 1	Berbari Dev Biol 360(1):66, 2011	AR	SLS9	NPHP
XPNPEP	X-prolyl aminopeptidase 3	O'Toole J Clin Invest 120(3):791, 2010	AR	NPHPL1	NPHP
FAN1	FANCI-associated nuclease 1	Zhou Nat Genet 44(8):910, 2012	AR	MTMR15	NPHP
PKHD1	PKHD1, Fibrocystin/Polyductin	Bergmann Kidney Int 67(3):829 2005	AR	ARPKD	NPHP
INPP5E	Inositol polyphosphate-5-phosphatase	Bielas Nat Genet 41(9):1032, 2009	AR	JBTS1	NPHP
TMEM21	Transmembrane Protein 216	Edvardson AJHG 86(1):93, 2010	AR	JBTS2	NPHP
AHI1	Abelson Helper Integration Site 1	Parisi J Med Gen 43(4):334 ,2005	AR	JBTS3	NPHP
ARL13B	ADP-ribosylation factot-like 13B	Cantagrel AJHG 83(2):170, 2008	AR	JBTS8	NPHP
CC2D2A	Coiled-coil and C2 domains-containing protein 2A	Noor AJHG 82(4):1011, 2008	AR	JBTS9	NPHP
KIF7	Kinesin family member 7	Putoux Nat Genet 43(6):601, 2011	AR	JBTS12	NPHP
TCTN1	tectonic family member 1	Garcia-Gonzalo Nat Genet 43(8):776, 2011	AR	JBTS13	NPHP
TMEM23	Transmembrane protein 237	Huang AJHG 89(6):713, 2011	AR	JBTS14	NPHP
CEP41	Centrosomal protein 41kDa	Lee Nat Genet 44(2):193, 2012	AR	TSGA14	NPHP
TMEM13	Transmembrane protein 138	Lee Science 335(6071): 966, 2012	AR	JBTS16	NPHP
C5orf42	Chromosome 5 open reading frame 42	Srour AJHG 90(4):693, 2012	AR	JBTS17	NPHP
TCTN3	Tectonic family member 3	Thomas AJHG 91(2):372, 2012	AR	JBTS18	NPHP
TMEM23	Transmembrane protein 231	Srour J Med Genet 49: 636-641, 2012	AR	JBTS20	NPHP
CSPP1	Centrosome spindle pole-associated protein 1	Akizu AJHG 94(1):80, 2014	AR	JBTS21	NPHP
PDE6D	Phosphodiesterase 6D	Thomas Hum Mutat 35(1):137, 2014	AR	JBTS22	NPHP
KIAA058	TALPID 3, chicken homolog of	Bachmann-Gagescu Hum Mutat 36(9):831, 2015	AR	JBTS23	NPHP
TCTN2	Tectonic family member 2	Huppke Eur J Hum Genet 23(5):616, 2015	AR	JBTS24	NPHP
CEP104	Centrosomal protein 41kDa	Korvatska Am J Med Genet B Neuropsychiatr Genet 1	AR	JBTS25	NPHP
KIAA055	Katanin-intereacting protein	Saunders Genome Biol 16:293, 2015	AR	JBTS26	NPHP
MKS1	Meckel syndrome, type 1	Kyttälä Nat Genet 38(2):155, 2006	AR	MKS1	NPHP
B9D1	B9 domain containing protein 1	Romani Orphanet J Rare Dis 9:72, 2014	AR	MKS9	NPHP
B9D2	B9 domain containing protein2	Dowdle AJHG 89(1): 94, 2011	AR	MKS10	NPHP
KIF14	Kinesin family member 14	Filges Clin Genet 86(3):220, 2013	AR	MKS12	NPHP
TMEM10	Transmembrane Protein 107	Shasheen Hum Mol Genet 24(18):5211, 2015	AR	MKS13	NPHP
BBS1	Bardet-Biedl Syndrome 1	Mykytyn Nat Genet 31(4):435, 2002	AR	BBS1	NPHP
BBS2	Bardet-Biedl Syndrome 2	Katsanis Science 293(5538):2256, 2001	AR	BBS2	NPHP
ARL6	Meckel syndrome, type 1	Khaddour Hum Mutat 28(5), 523, 2007	AR	BBS3	NPHP
BBS4	Bardet-Biedl Syndrome 4	Mykytyn Nat Genet 8(2):188, 2001	AR	BBS4	NPHP
BBS5	Bardet-Biedl Syndrome 5	Tieder Int J Pediatr Nephrol 3(3):199, 1982	AR	BBS5	NPHP
MKKS	Bardet-Biedl Syndrome 6	Katsanis Nat Genet26(1):67, 2000	AR	BBS6	NPHP
BBS7	Bardet-Biedl Syndrome 7	Badano AJHG 72(3), 650, 2003	AR	BBS7	NPHP
TTC8	Bardet-Biedl Syndrome 8	Stoetzel J Hum Genet 51(1):81, 2005	AR	BBS8	NPHP
PTHB1	Bardet-Biedl Syndrome 9	Nishimura AJHG 77(6):1021, 2005	AR	BBS9	NPHP
BBS10	Bardet-Biedl Syndrome 10	Stoetzel Nat Genet 38(5):521, 2006	AR	BBS10	NPHP
TRIM32	Bardet-Biedl Syndrome 11	Chiang Proc Natl Acad Sci USA 103(16):6287,2006	AR	BBS11	NPHP
BBS12	Bardet-Biedl Syndrome 12	Stoetzel AJHG 80(1):1, 2007	AR	BBS12	NPHP
WDPCP	WD repeat-containing planar cell polarity effector	Stone Nat Genet 25(1):79, 2000	AR	BBS15	NPHP
LZTFL1	Bardet-Biedl syndrome 17	Marion J Med Genet 49(5):317, 2012	AR	BBS17	NPHP
BBIP1	Bardet-Biedl syndrome 18	Scheidecker J Med Genet 51(2):132, 2014	AR	BBS18	NPHP
IFT27	Bardet-Biedl Syndrome 20	Schaefer J Med Genet 61(5):447 2016	AR	BBS20	NPHP
DDX59	Orofaciodigital syndrome 5	Shamseldin AJHG 93(3):555, 2013	AR	OFD5	NPHP
SCLT1	Orofaciodigital syndrome 9	Adly Hum Mutation 35(1): 36, 2013	AR	OFD9	NPHP
C2CD3	Orofaciodigital syndrome 14	Thauvin-Robinet Nature Genet 46(8):905, 2014.	AR	OFD14	NPHP
KIAA075	Orofaciodigital syndrome 15	Chevrier Hum Mol Genet 25(3):497, 2016	AR	OFD15	NPHP
IFT122	Intraflagellar transport 122	Walczak-Sztulpa AJHG 86(6):949, 2010	AR	CED1	NPHP
WDR35	WD repeat domain 35	Gilissen AJHG 87(3):418, 2010	AR	CED2	NPHP
IFT43	intraflagellar transport 43	Gilissen AJHG 87(3):418, 2010	AR	CED3	NPHP
IFT80	Intraflagellar Transport 80	Beales Nat Genet 39(6):727 2007	AR	SRTD2	NPHP
****DYNC2***	Dynein cystoplasmic 2 heavy chain	El Hokayem J Med Genet 49(4):227, 2012	AR	SRTD3	NPHP
NEK1	NIMA Related Kinase 1	Thiel AJHG 88(1):106, 2011	AR	SRTD6	NPHP
WDR60	WD Repeat Domain 60	McInerney-Leo AJHG 93(3): 515, 2013	AR	SRTD8	NPHP
****IFT140***	intraflagellar transport 140	Perrault AJHG 90(5):864, 2012	AR	SRTD9	NPHP
WDR34	WD Repeat Domain 34	Schmidts AJHG 93(5):932, 2013	AR	SRTD11	NPHP
CEP120	Centrosomal protein 120kDa	Shaheen Hum Mol Genet. 24(5):1410, 2015	AR	SRTD13	NPHP
IFT57	Intraflagellar Transport 57	Bruel J Med Genet 54(6):371, 2017	AR		NPHP
IFT52	Intraflagellar Transport 52	Girisha Clin Genet 90(6):536, 2016	AR	SRTD	NPHP
ALMS1	Altrom Syndrome Protein 1	Collin Nat Genet 31(1):74, 2002	AR	ALMS	NPHP
PIK3R4	Phosphatidyllinositol 3-kinase, regulatorry subunit 4	Panaretou J Biol Chem 272(4): 2477, 1997	AR	VPS15	NPHP
TXNDC1	Thioredoxin domain-containing protein 15	Honjo Cell Rep 16(2):295, 2016	AR		NPHP
SLC41A1	Solute carrier member family 41, member 1	Hurd JASN 24(6):967, 2013	AR		NPHP
POC1B	Cone rod dystrophy 20	Roosing AJHG 95(2):131, 2014	AR		NPHP
HSD17B	17-Beta-Hydroxysteroids hydrogenase IV	Boehmer J Clin Endocrinol Metab 84(12):4713, 1999	AR	MFP2	NPHP
USH2A	Usherin 2A	Smith Genomics 14(4):995, 1992	AR		NPHP
UMOD	Uromodulin	Hart J Med Genet 39(12):882, 2002	AD		NPHP
HOXA4	Heomobox A4	Acampora Nucleic Acids Res 17(24):10385, 1985	AD		NPHP
HOXB6	Heomobox B6	Kaur J Exp Zool 264(3):323, 1992	AD		NPHP
TBC1D3	TBC1 Domain Family Member 32	Adly Hum Mutat 35:36, 2014	AD		NPHP
CCDC28	Coiled-coil domain containing protein 28B	Cardenas-Rodriguez Hum Genet 132(1):91, 2013	AR/DR		NPHP
EVC	EvC Ciliary Complex Subunit 1	Ruiz-Perez Nat Genet. 24(3):283, 2000	AR/AD		NPHP
EVC2	EvC Ciliary Complex Subunit 2	Kurian Indian J Dent Res. 18(1):31, 2007	AR/AD		NPHP

Supplementary Table S2 (continued)

Gene	Protein	Reference	Mode of i	Category
MYCN	Feingold Syndrome	Marcelis Hum. Mut. 29:1125, 2006	AD	Syndromic CAKUT
NFIX	Nuclear Factor I X	Malan Am J Hum Genet 87:189, 2010	AD	Syndromic CAKUT
NOTCH2	Notch 2	Kamath Nat Rev Nephrol 9:409, 2013	AD	Syndromic CAKUT
PAX8	Paired Box 8	Meeus J Clin Endocrinol Metab 89:4285, 2004	AD	Syndromic CAKUT
PKD1	Polycystin 1, Transient Receptor Potential Channel Interacting	Rossetti J Am Soc Nephrol 18:2143, 2007	AD	Syndromic CAKUT
PKD2	Polycystin 2, Transient Receptor Potential Cation Channel	Rossetti J Am Soc Nephrol 18:2143, 2007	AD	Syndromic CAKUT
PROKR2	Prokineticin Receptor 2	Sarfati Front Horm Res 39:121, 2010	AD	Syndromic CAKUT
PTPN11	Protein Tyrosine Phosphatase, Non-Receptor Type 11	Bertola Am J Med Genet 130A:378, 2004	AD	Syndromic CAKUT
RAF1	Raf-1 Proto-Oncogene, Serine/Threonine Kinase	Razzaque Nat Genet 39:1013, 2007	AD	Syndromic CAKUT
RAI1	Retinoic Acid Induced 1	Vilboux PLoS One 6:e22861, 2011	AD	Syndromic CAKUT
SALL4	Spalt Like Transcription Factor 4	Kohlhase GeneReviews®Book Section, 1993	AD	Syndromic CAKUT
SEMA3A	Semaphorin 3A	Young Hum Reprod 27:1460, 2012	AD	Syndromic CAKUT
SEMA3E	Semaphorin 3E	Lalani J Med Genet 41:e94, 2004	AD	Syndromic CAKUT
SETBP1	SET Binding Protein 1	Schinzel Am J Med Genet 1:361, 1978	AD	Syndromic CAKUT
SHH	Sonic Hedgehog	Lurie Am J Med Genet 35:286, 1990	AD	Syndromic CAKUT
SF3B4	Splicing Factor 3b Subunit 4	Bernier Am J Hum Genet 90:925, 2012	AD	Syndromic CAKUT
SNAP29	Synaptosome Associated Protein 29	Lopez-Rivera NEJM 376:742, 2017	AD	Syndromic CAKUT
SOS1	SOS Ras/Rac Guanine Nucleotide Exchange Factor 1	Ferrero Eur J Med Genet 51:566, 2008	AD	Syndromic CAKUT
SOX9	SRY-Box 9	Airik Hum Mol Genet 19:4918, 2010	AD	Syndromic CAKUT
SRCAP	Snf2 Related CREBBP Activator Protein	Hood Am J Hum Genet 90:308, 2012	AD	Syndromic CAKUT
TBX1	T-Box 1	Kujat Am J Med Genet A 140:1601, 2006	AD	Syndromic CAKUT
TBX3	T-Box 3	Meneghini Eur J Med Genet 49:151, 2006	AD	Syndromic CAKUT
TFAP2A	Transcription Factor AP-2 Alpha	Milunsky Am J Hum Genet 82:1171, 2008	AD	Syndromic CAKUT
TP63	Tumor Protein P63	Celli Cell 99:143, 1999	AD	Syndromic CAKUT
TRPS1	Zinc finger transcription factor; Trichorhinophalangeal syndrome	Tasic Ren Fail 36:619, 2014	AD	Syndromic CAKUT
TSC1	Tuberous Sclerosis 1	Curatolo Lancet 372:657, 2008	AD	Syndromic CAKUT
TSC2	Tuberous Sclerosis 2	Kumar Hum Mol Genet 4:1471, 1995	AD	Syndromic CAKUT
WNT5A	Wnt Family Member 5A	Roifman Clin Genet 87:34, 2015; Person Dev Dyn 239:327, 2010	AD	Syndromic CAKUT
ARID1B	AT-Rich Interaction Domain 1B	Levy J Med Genet 28, 1991	AD/ AR	Syndromic CAKUT
DIS3L2	DIS3 Like 3'-5' Exoribonuclease 2	Astuti Nat Genet 5;44:277, 2012	AD/ AR	Syndromic CAKUT
FGFR2	Fibroblast Growth Factor Receptor 2	LeHeup Eur J Pediatr 154:130, 1995	AD/ AR	Syndromic CAKUT
GDF6	Growth Differentiation Factor 6	Tassabehji Hum Mutat 29:1017, 2008	AD/ AR	Syndromic CAKUT
GLI3	GLI Family Zinc Finger 3	Cain PLoS One 4:e7313, 2009	AD/ AR	Syndromic CAKUT
PCSK5	Proprotein Convertase Subtilisin & Kexin Type 5	Nakamura BMC Res Notes 8:228, 2015	AD/ AR	Syndromic CAKUT
PTEN	Phosphatase And Tensin Homolog	Reardon J Med Genet 38:820, 2001	AD/ AR	Syndromic CAKUT
RPS24	Ribosomal Protein S24	Yetgin Turk J Pediatr 36:239, 1994	AD/ AR	Syndromic CAKUT
VANGL1	VANGL Planar Cell Polarity Protein 1	Bartsch Mol Syndromol 3:76, 2012	AD/ AR	Syndromic CAKUT
AXIN1	Axin 1	Oates Am J Hum Genet 79:155, 2006	De novo	Syndromic CAKUT
H19	H19, Imprinted Maternally Expressed Transcript (Non-Protein Coding)	Hur Proc Natl Acad Sci U S A 113:10938, 2016	De novo	Syndromic CAKUT
KCNQ1OT1	KCNQ1 Opposite Strand & Antisense Transcript 1 (Non-Protein Coding)	Chiesa Hum Mol Genet 21:10, 2012	De novo	Syndromic CAKUT
NIPBL	NIPBL, Cohesin Loading Factor	Rohatgi Am J Med Genet 152A:1641, 2010	De novo	Syndromic CAKUT
CDKN1C	Cyclin Dependent Kinase Inhibitor 1C	Mussa Pediatr Nephrol 27:397, 2012	De novo	Syndromic CAKUT
CHD7	Chromodomain Helicase DNA Binding Protein 7	Janssen Hum Mutat 33:1149 2012	De novo	Syndromic CAKUT
AMER1	APC Membrane Recruitment Protein 1	Pellegrino Am J Med Genet 16:159, 1997	XL	Syndromic CAKUT
ATP7A	ATPase Copper Transporting Alpha	Vulpe Nat Genet 3:7, 1993	XL	Syndromic CAKUT
BCOR	BCL6 Corepressor	Ng Nat Genet 36:411, 2004	XL	Syndromic CAKUT
DLG3	Disc large, drosphilia, homologue of 3	Philips Orphanet J Rare Dis 9:49, 2014	XL	Syndromic CAKUT
FAM58A	Family With Sequence Similarity 58 Member A	Green J Med Genet 33:594, 1996; Unger Nat Genet 40:287, 2008	XL	Syndromic CAKUT
FLNA	Filamin A	Robertson Am J Med Genet A 140:1726, 2006	XL	Syndromic CAKUT
GPC3	Glypican 3	Cottereau Am J Med Genet C Semin Med Genet 163:92, 2013	XL	Syndromic CAKUT
MID1	Midline 1	Preiksaitiene Clin Dysmorphol 24:7, 2015	XL	Syndromic CAKUT
NSDHL	NAD(P) Dependent Steroid Dehydrogenase-Like	König J Am Acad Dermatol 46:594, 2002	XL	Syndromic CAKUT
OFD1	Oral-Facial-Digital Syndrome 1 Protein	Bisschoff Hum Mutat 34:237, 2013	XL	Syndromi Feather H
PIGA	Phosphatidylinositol Glycan Anchor Biosynthesis Class A	Johnston Am J Hum Genet 90:295, 2012	XL	Syndromic CAKUT
PORCN	Porcupine O-Acyltransferase	Suskan Pediatr Dermatol 7:283, 1990	XL	Syndromic CAKUT
SMC1A	Structural Maintenance Of Chromosomes 1A	Deardorff GeneReviews® Book Section Seattle(WA), 1993	XL	Syndromic CAKUT
UPF3B	UPF3B, Regulator Of Nonsense Mediated MRNA Decay	Lynch Eur J Med Genet 55:476, 2012	XL	Syndromic CAKUT
ZIC3	Zic Family Member 3	Chung Am J Med Genet 155:1123, 2011	XL	Syndromic CAKUT
GDF11	Growth Differentiation Factor 11	Tsuda Eur J Pediatr Surg 21:238, 2011	Unknown	Syndromic CAKUT
OSR1	Odd-Skipped Related Transciption Factor 1	Zhang Hum Mol Genet 20:4167, 2011	Unknown	Syndromic CAKUT
TTC30A	Tetratricopeptide Repeat Domain 30A	Hilger Hum Mutat 36: 1150, 2015	Unknown	Syndromic CAKUT
UBE3A	Ubiquitin-protein ligase E3A	Abaied AJHG 152A:141, 2010	Unknown	Syndromic CAKUT
SH2B1	SH2B Adaptor Protein 1	Sampson Am J Med Genet 152:2618, 2010	Unknown	Syndromic CAKUT
ACE	Angiotensin I-converting enzyme	Gribouval Nat Genet 37:964, 2005	AR	isolated CAKUT
AGT	Angiotensinogen	Gribouval Nat Genet 37:964, 2005	AR	isolated CAKUT
AGTR1	Angiotensin II receptor, type 1	Gribouval Nat Genet 37:964, 2005	AR	isolated CAKUT
CHRM3	Muscarinic acetylcholine receptor M3	Weber AJHG 19:634, 2011	AR	isolated CAKUT
ETV4	ETS translocation variant 4, E1A enhancer binding protein	Chen IJPCH 4:61, 2016	AR	isolated CAKUT
FRAS1	Extracellular matrix protein FRAS1	Kohl JASN 25:1917, 2014	AR	isolated CAKUT
FREM1	FRAS1 related extracellular matrix protein 1	Kohl JASN 25:1917, 2014	AR	isolated CAKUT
****FREM2***	FRAS1 related extracellular matrix protein 2	Kohl JASN 25:1917, 2014	AR	isolated CAKUT
GRIP1	Glutamate receptor interacting protein 1	Kohl JASN 25:1917, 2014	AR	isolated CAKUT
HPSE2	Heparanase 2 (Inactive)	Bulum Nephron 130:54, 2015	AR	isolated CAKUT
ITGA8	Integrin α8	Humbert AJHG 189:1260, 2014	AR	isolated CAKUT
REN	Renin	Gribouval Nat Genet 37:964, 2005	AR	isolated CAKUT
TRAP1	Heat-shock protein 75 (also known as TNF receptor-associated protein 1)	Saisawat KI 85:880, 2014	AR	isolated CAKUT
FGF20	Fibroblast Growth Factor 20	Barak Dev Cell 22:1191, 2012	AR	isolated CAKUT
BMP4	Bone morphogenic protein 4	Weber JASN 19:891, 2008	AD	isolated CAKUT
CHD1L	Chromodomain helicase DNA binding protein 1-like	Brockschmidt NDT 27:2355, 2012	AD	isolated CAKUT
CRKL	CRK Like Proto-Oncogene, adaptor protein	Lopez-Rivera NEJM 376:742, 2017	AD	isolated CAKUT
DSTYK	Dual serine/threonine and tyrosine protein kinase	Sanna-Cherchi NEJM 369:621, 2013	AD	isolated CAKUT
EYA1	Eyes absent homolog 1	Abdelhak Nat Genet 15:157, 1997	AD	isolated CAKUT
GATA3	GATA binding protein 3	Pandolfi Nat Genet 11:40, 1995; Van Esch Nature 406:419, 2000	AD	isolated CAKUT
GEN1	GEN1 Holliday Junction 5' Flap Endonuclease	Wang X, Wang H, Liu J, Gong Y, et al. Int J Biol Sci. 2018 Jan 1;14(1):10-20.	AR	isolated CAKUT
GREB1L	Growth Regulation By Estrogen In Breast Cancer 1 Like	Brophy Genetics 207:215, 2017	AD	isolated CAKUT
HNF1B	HNF homeobox B	Lindner Hum Mol Genet 24:263, 1999	AD	isolated CAKUT
MUC1	Mucin 1	Kirby Nat Genet 45:299, 2013	AD	isolated CAKUT
NRIP1	Nuclear Receptor Interacting Protein 1	Vivante JASN 28:2364, 2107	AD	isolated CAKUT
PAX2	Paired box 2	Sanyanusin Hum Mol Genet 4:2183, 1995	AD	isolated CAKUT
PBX1	PBX Homeobox 1	Heidet JASN 28:2901, 2017	AD	isolated CAKUT
PRDM15	R/SET Domain 15	Sci Adv. 2020 Jan 10;6(2):eaax9852. doi: 10.1126/sciadv.aax9852.	AD	isolated CAKUT
RET	Proto-oncogene tyrosine-protein kinase receptor Ret	Skinner AJHG 82:344, 2008	AD	isolated CAKUT
ROBO2	Roundabout, axon guidance receptor, homolog 2 (Drosophila)	Hwang Hum Genet 134:905, 2015; Lu AJHG 80:616, 2007	AD	isolated CAKUT
SALL1	Sal-like protein 1 (also known as spalt-like transcription factor 1)	Kohlhase Nat Genet 18:81, 1998	AD	isolated CAKUT
SIX1	SIX homeobox 1	Ruf Proc. Nat. Acad. Sci. 101: 8090, 2004	AD	isolated CAKUT
SIX2	SIX homeobox 2	Weber JASN 19:891, 2008	AD	isolated CAKUT
SIX5	SIX homeobox 5	Hoskins AJHG 80:800, 2007	AD	isolated CAKUT
SLIT2	Slit homolog 2	Hwang Hum Genet 134:905, 2015	AD	isolated CAKUT
SOX17	Transcription factor SIX-17	Gimelli Hum Mut 31:1352, 2010	AD	isolated CAKUT
SRGAP1	SLIT-ROBO Rho GTPase activating protein 1	Hwang Hum Genet 134:905, 2015	AD	isolated CAKUT
TBX6	T-Box transcription factor	N Engl J Med. 2015 Jan 22;372(4):341-50.	AD	isolated CAKUT
TBX18	T-Box transcription factor	Vivante AJHG 97:291, 2015	AD	isolated CAKUT
TNXB	Tenascin XB	Gbadegesin JASN 24:1313, 2013	AD	isolated CAKUT
UPK3A	Uroplakin 3A	Jenkins JASN 16:2141, 2005	AD	isolated CAKUT
WNT4	Protein Wnt-4	Biason-Lauber NEJM 351:792, 2004; Mandel AJHG 82:39, 2008; Vivante JASN 24:550, 2013	AD	isolated CAKUT
KAL1	Anosmin 1	Hardelin PNAS 89:8190, 1992	XL	isolated CAKUT
ZMYM2	Zinc Finger MYM-Type Containing 2	Connaughton DM, Dai R, Owen DJ, et al. Am J Hum Genet. 2020 Aug 31:S0002-9297(20)3028	AD	isolated CAKUT

Supplementary Table S2 (continued)

Gene	Protein	Reference	Mode of i	Category
ADAMTS	ADAM metallopeptidase with thrombospondin type 1 motif 13	Levy Nature 413: 488, 2001	AR	GN
CFI	Complement factor I	Fremeaux-Bacchi J Med Genet 41:e84, 2004	AR	GN
COL4A4	Collagen type IV alpha 4 chain	Mochizuki Nat Genet 8:77, 1994	AR	GN
CFB	CFB	Goicoechea de Jorge Proc Nat Acad Sci 104:240, 2007	AD	GN
CFHR3	CFHR3	Zipfel PLoS Genet 3:e41, 2007	AD	GN
CFHR5	Complement factor H related 5	Gale Lancet 376:794, 2010	AD	GN
EIF2AK3	Eukaryotic Translation Initiation Factor 2 Alpha Kinase 3	Delepine Nature Genet 25:406, 2000	AR	GN
FN1	Fibronectin	Castelletti Proc Nat Acad Sci 105:2538, 2008	AD	GN
FOXC2	Forkhead Box C2	Yildirim-Toruner AJMG 131A:281, 2004	AD	GN
GSN	Gelsolin	Maury FEBS Lett 260:85, 1990	AD	GN
LYZ	Lysozyme	Pepys Nature 362:553, 1993	AD	GN
THBD	Thrombomodulin	Delvaeye NEJM 361:345, 2009	AD	GN
SPRY2	Sprouty RTK Signaling Antagonist 2	Milillo Europ J Hum Genet 23:1673, 2015	AD	GN
C3	Complement C3	Fremeaux-Bacchi Blood 112:4948, 2008	AR/AD	GN
CD46	CD46 molecule	Noris Lancet 362:1542, 2003	AR/AD	GN
CFH	Complement factor H	Edelsten Arch Dis Child 53:255, 1978	AR/AD	GN
CFHR1	Complement factor H related 1	Zipfel PLoS Genet 3:e41, 2007	AR/AD	GN
COL4A3	Collagen type IV alpha 3 chain	Lemmink Hum Mol Genet 3:1269, 1994	AR/AD	GN
COL4A5	Collagen type IV alpha 5 chain	Antignac J Clin Invest 93:1195, 1994	XL	GN
COL4A6	Collagen type IV alpha 6 chain	Renieri Hum Mutat 4:195, 1994	XL	GN
ATP6B1	ATPase H+ Transporting V1 Subunit B1	Karet Nat Genet 21:84, 1999	AR	TK
ATP6V1C	ATPase H+ Transporting V1 Subunit C2	Stover J Med Genet 39:796, 2002	AR	TK
BCS1L	BCS1 Homolog, Ubiquinol-Cytochrome C Reductase Complex Chaperone	de Lonlay Nat Genet 29:57, 2001	AR	TK
BSND	Barttin CLCNK Type Accessory Beta Subunit	Birkenhager Nat Genet 29:310, 2001	AR	TK
COG6	Component Of Oligomeric Golgi Complex 6	Huybrechts JIMD Rep 4:103, 2012	AR	TK
COQ9	Coenzyme Q9	Duncan AJHG 84:558, 2009	AR	TK
CYP11B1	Cytochrome P450 Family 11 Subfamily B Member 1	Pascoe AJHG 51: A28, 1992	AR	TK
CYP17A1	Cytochrome P450 Family 17 Subfamily A Member 1	Kagimoto Molec Endocr 2:564, 1988	AR	TK
CYP27B1	Cytochrome P450 Family 27 Subfamily B Member 1	Wang AJHG 63:1694, 1998	AR	TK
EGF	Epidermal Growth Factor	Groenestege J Clin Invest 117:2260, 2007	AR	TK
FGF23	Fibroblast Growth Factor 23	Benet-Pages Hum Molec Genet 14:385, 2005	AR	TK
GALNT3	Polypeptide N-Acetylgalactosaminyltransferase 3	Barbieri J Hum Genet 52:464, 2007	AR	TK
HSD11B2	Hydroxysteroid 11-Beta Dehydrogenase 2	Wilson J Clin Endocr Metab 80:2263, 1995	AR	TK
MRPS22	Mitochondrial Ribosomal Protein S22	Saada J Med Genet 44:784, 2007	AR	TK
RRM2B	Ribonucleotide Reductase Regulatory TP53 Inducible Subunit M2B	Bourdon (Letter) Nature Genet 39:776, 2007	AR	TK
SCNN1A	Sodium Channel Epithelial 1 Alpha Subunit	Chang Nature Genet 12:248, 1996	AR	TK
SCNN1B	Sodium Channel Epithelial 1 Beta Subunit	Chang Nature Genet 12:248, 1996	AR	TK
SLC12A3	Solute Carrier Family 12 Member 3	Ng Neurology 67:1080, 2006	AR	TK
SLC26A4	Solute Carrier Family 26	Everett Nat Genet 17:411, 1997	AR	TK
SLC2A2	Solute Carrier Family 2 Member 2	Manz Pediatr Nephrol 1:509, 1987	AR	TK
SLC4A4	Solute Carrier Family 4 Member 4	Igarashi JASN 12:713, 2001	AR	TK
SLC4A5	Solute Carrier Family 4 Member 5	Igarashi (Letter) Nature Genet 23:264, 1999	AR	TK
SLC6A19	Solute Carrier Family 6 Member 19	Kleta Nat Genet 36:999, 2004	AR	TK
SLC7A7	Solute Carrier Family 7 Member 7	Borsani Nature Genet 21:297, 1999	AR	TK
SUCLA2	Succinate-CoA ligase, ADP-forming beta subunit	Carrozzo Brain 130: 862, 2007	AR	TK
TRPM6	Transient Receptor Potential Cation Channel Subfamily M Member 6	Schlingmann Nature Genet 31:166, 2002	AR	TK
VIPAR	Vacuolar Protein Sorting 33 Homolog B (Yeast)	Cullinane Nature Genet 42:303 2010	AR	TK
VIPAS39	VPS33B-Interacting Protein Involved In Polarity And Apical Protein Restricti	Cullinane Nature Genet 42:303 2010	AR	TK
WNK1	WNK Lysine Deficient Protein Kinase 1	Lafreniere AJHG 74:1064, 2004	AR	TK
WNK4	WNK Lysine Deficient Protein Kinase 4	Wilson Science 293:1107, 2001	AR	TK
AP2S1	Adaptor Related Protein Complex 2 Sigma 1 Subunit	Nesbit Nat Genet 45:93, 2013	AD	TK
AVP	Arginine Vasopressin	Abbes Clin Chem 46:1699, 2000	AD	TK
CACNA1	Calcium Voltage-Gated Channel Subunit Alpha1 S	Miller Neurology 63:1647, 2004	AD	TK
CNNM2	Cyclin And CBS Domain Divalent Metal Cation Transport Mediator 2	Stuiver AJHG 88: 333, 2011	AD	TK
CUL3	Cullin 3	Boyden Nature 482:98, 2012	AD	TK
EHHADH	Enoyl-CoA Hydratase And 3-Hydroxyacyl CoA Dehydrogenase	Klootwijk NEJM 370:129, 2014	AD	TK
FXYD6-F	FXYD6-FXYD2 Readthrough	Meij Nature Genet 26:265, 2000	AD	TK
GNA11	G Protein Subunit Alpha 11	Li J Clin Endocr Metab 99:E1774, 2014	AD	TK
KCNJ5	Potassium Voltage-Gated Channel Subfamily J Member 5	Charmandari J Clin Endocr Metab 97:E1532, 2012	AD	TK
KLHL3	Kelch Like Family Member 3	Louis-Dit-Picard Nat Genet 44:609, 2012	AD	TK
NR3C2	Nuclear Receptor Subfamily 3 Group C Member 2	Riepe J Clin Endocr Metab 91:4552, 2006	AD	TK
SAC (AD	Sacsin Molecular Chaperone (Adenylate Cyclase 10, Soluble)	Reed J Clin Endocr Metab 87:1476, 2002.	AD	TK
SCN4A	Sodium Voltage-Gated Channel Alpha Subunit 4	Ptacek Cell 67:1021, 1991	AD	TK
CLCNKA	Chloride Voltage-Gated Channel Ka	Nozu J Med Genet 45: 182, 2008	DR	TK
CLCKNB	Chloride Voltage-Gated Channel Ka	Nozu J Med Genet 45: 182, 2008	AR/DR	TK
SLC36A2	Solute Carrier Family 36 Member 2	Broer JCI 118:3881, 2008	AR/DR	TK
SLC6A20	Solute Carrier Family 6 Member 20	Broer JCI 118:3881, 2008	AR/DR	TK
AQP2	Aquaporin 2	Knoers Europ J Pediat 150:370, 1991	AR/AD	TK
SCNN1G	Sodium Channel Epithelial 1 Gamma Subunit	Hansson Nat Genet 11:76, 1995	AR/AD	TK
SLC5A2	Solute Carrier Family 5 Member 2	van den Heuvel Hum Genet 111:544, 2002	AR/AD	TK
AVPR2	Arginine Vasopressin Receptor 2	Feldman NEJM 352: 1884-1890, 2005	XLR	TK
AGXT	Alanine-glyoxylate aminotransferase	Purdue Proc Natl Acad Sci U S A 88:10900, 1991	AR	Stone
ALDOB	Aldolase B fructose biphosphate	Paolella Hum Genet 77:115, 1987	AR	Stone
ALPL	Alkaline Phosphatase, Liver	Weiss Proc Natl Acad Sci U S A 85:7666, 1988	AR	Stone
APRT	Adenine phosphoribosyltransferase	Hidaka J Clin Invest 80:1409, 1987	AR	Stone
ATP6V0A	ATPase, H+ transporting, lysosomal V0 subunit a4	Smith Nat Genet 26:71, 2000	AR	Stone
ATP6V1B	ATPase, H+ transporting, lysosomal 56/58kDa, V1 subunit B1	Karet Nat Genet 21:84, 1999	AR	Stone
ATP7B	ATPase, Cu(2+)-transporting, beta polypeptide	Gromadzka Clin Genet 68:524, 2005	AR	Stone
CA2	Carbonic anhydrase II	Venta AJHG 49:1082, 1991	AR	Stone
CLCNKB	Chloride channel, voltage-sensitive Kb	Simon Nat Genet 17:171, 1997	AR	Stone
CLDN16	Claudin 16	Simon Science 285:103, 1999	AR	Stone
CLDN19	Claudin 19	Konrad AJHG 79:949, 2006	AR	Stone
CTNS	Cystinosin	Town Nat Genet 18:319, 1998	AR	Stone
CYP24A1	Cytochrome P450, family 24, subfamily A, polypeptide 1	Schlingmann NEJM 36:410, 2011	AR	Stone
ENPP1	Ectonucleotide Pyrophosphatase/Phosphodiesterase 1	Am J Med Genet A. 2019 Oct;179(10):2112-2118.	AR	Stone
FAH	Fumarylacetoacetate hydrolase	Aponte Proc Nat Acad Sci 98:641, 2001	AR	Stone
FAM20A	Family with sequence similarity 20, member A	Jaureguiberry Nephron Physiol 122:1, 2012	AR	Stone
G6PC	Glucose 6 phosphate catalytic	Seydewitz Hum Mutat 15:115, 2000	AR	Stone
GRHPR	Glyoxylate reductase/hydroxypyruvate	Cramer Hum Mol Genet 8:2063, 1999	AR	Stone
HOGA1	4-hydroxy-2-oxoglutarate aldolase 1	Belostotsky AJHG 87:392, 2010	AR	Stone
KCNJ1	Potassium inwardly-rectifying channel, subfamily J, member 1	Simon Nat Genet 14:152, 1996	AR	Stone
KCNJ10	Potassium Channel inwardly rectifyinh subfamily J, member 10	Bockenhauer NEJM 360:1960, 2009	AR	Stone
SLC12A1	Solute carrier family 12, member 1	Simon Nat Genet 13:183, 1996	AR	Stone
SLC26A1	Solute carrier family 26 (sulfate transporter), member 1	Gee AJHG 98:1228, 2016	AR	Stone
SLC2A2	Solute carrier family 2 (faciliated glucose transporter)	Akagi J Hum Genet 45:60, 2000	AR	Stone
SLC34A3	Solute carrier family 34 (sodium	Lorenz-Depiereux AJHG 78:193, 2006	AR	Stone
XDH	Xanthine dehydrogenase	Ichida J Clin Invest 99:2391, 1997.	AR	Stone
HNF4A	Hepatocyte nuclear factor 4, alpha	Hamilton J Med Genet 51:165, 2014	AD	Stone
SLC9A3R	Solute carrier family 9, subfamily A	Karim NEJM 359:1128, 2008	AD	Stone
CASR	Calcium-sensing receptor	Pearce NEJM 335:1115, 1996	AD/AR	Stone
SLC22A1	Solute carrier family 22	Enomoto Nature 417:447, 2002	AD/AR	Stone
SLC2A9	Solute carrier family 2	Matsuo AJHG 83:744, 2008	AD/AR	Stone
SLC34A1	Solute carrier family 34	Prie NEJM 347:983, 2002	AD/AR	Stone
SLC3A1	Solute carrier family 3, member 1	Calonge Nat Genet 6:420, 1994	AD/AR	Stone
SLC4A1	Solute carrier family 4, anion exchanger, member 1	Bruce J Clin Invest 100:1693, 1997	AD/AR	Stone
SLC7A9	Solute carrier family 7 (glycoprotein associated	Feliubadalo Nat Genet 23:52, 1999	AD/AR	Stone
VDR	Vitamin D (1,25- dihydroxyvitamin D3) receptor	Scott JASN 10:1007, 1999	AD/AR	Stone
CLCN5	Chloride channel, voltage-sensitive 5	Lloyd Nature 379:445, 1996	XL	Stone
HPRT1	Hypoxanthine	Davidson AJHG 48:951, 1991	XL	Stone
OCRL	Oculocerebrorenal syndrome of Lowe	Reilly AJHG 42:748, 1988	XL	Stone

Supplementary Table S3. A virtual renal development gene panel

Gene	Protein	PubMed or MGI Reference ID	Inheritance (Mouse)
Ace	Angiotensin I converting enzyme	8642790	AR
Acvr2b	Activin A Receptor Type 2B	9242489	AR
Adamts1	ADAM Metallopeptidase With Thrombosp	10811842	AR
Agt	Angiotensinogen	8675666	AR
Agtr1a	Angiotensin II receptor, type 1a	10024874	AR
Agtr1b	Angiotensin II receptor, type 1b	10024874	AR
Agtr2	Angiotensin II Receptor Type 2	10024874	XR*(null mice)
Aldh1a2	Aldehyde Dehydrogenase 1 Family Memb	20040494	AR
Amer1	APC Membrane Recruitment Protein 1	21571217	AR
Anp32b	Acidic Nuclear Phosphoprotein 32 Family	21636789	AR
Aprt	Adenine Phosphoribosyltransferase	8864750	AR
Aqp2	Aquaporin 2	3184310	AR
Arhgap1	Rho GTPase Activating Protein 1	17227869	AR
Arhgap35	Rho GTPase Activating Protein 35	26859289	AR
Arid5b	AT-Rich Interaction Domain 5B	17143286	AR
Arl3	ADP Ribosylation Factor Like GTPase 3	16565502	AR
Atmin	ATM Interactor	24852369	AR
Atp7a	ATPase Copper Transporting Alpha	11534785	XR
Axin1	Axin 1	17246824 13340237	AD/AR
Bag6	BCL2 Associated Athanogene 6	16287848	AR
Bcl2	BCL2, Apoptosis Regulator	8623928	AR
Bmp4	Bone Morphogenetic Protein 4	10749566	AD
Bmp5	Bone Morphogenetic Protein 5	5692092	AD/AR
Bmp7	Bone Morphogenetic Protein 7	7590254	AR
Bmper	BMP Binding Endothelial Regulator	17035289	AR
Cdc42	Cell Division Cycle 42	23555292	AR
Cdh4	Cadherin 4	11839813	AR
Cdh6	Cadherin 6	10864459	AR
Chrm3	Cholinergic Receptor Muscarinic 3	10944224	AR
Cntrl	Centriolin		?
Crb3	Crumbs 3, Cell Polarity Complex Compor	26631503	AR
Crim1	Cysteine Rich Transmembrane BMP Reg	22511315	AR
Ctdnep1	CTD Nuclear Envelope Phosphatase 1	23360989	?
Ctnnb1	Catenin Beta 1	20454682	AD/AR
Ctnnbip1	Catenin Beta Interacting Protein 1	17803964	AR
Cxcr4	C-X-C Motif Chemokine Receptor 4	J:175213	?(Hom)
Cyp26a1	Cytochrome P450 Family 26 Subfamily A	11157778	AR
Dact1	Dishevelled Binding Antagonist Of Beta C	20145239	AR
Dchs1	Dachsous Cadherin-Related 1	21303848	AR
Dhcr7	7-Dehydrocholesterol Reductase	11230174	AR
Dlg1	Discs Large MAGUK Scaffold Protein 1	17172448	AR
Dlg5	Discs Large MAGUK Scaffold Protein 5	17765678	AR
Dnah11	Dynein Axonemal Heavy Chain 11	J:175213	?(hom)
Dnah5	Dynein Axonemal Heavy Chain 5	J:175213	?(hom)
Dym	Dymeclin	18852472	AR
Efnb2	Ephrin B2	15223334	AD
Emx2	Empty Spiracles Homeobox 2	9165114	AR
Esrrg	Estrogen Related Receptor Gamma	21138943	AR
Etl4/Etn2	Early transposon element insertion site 2	23436999	AD/AR
Etv4	ETS variant 4	19898483	AR
Etv5	ETV variant 5	19898483	AR
Exoc5	Exocyst complex component 5	26046524	AR
Eya1	EYA Transcriptional Coactivator And Pho	10471511	AD/AR
Fat4	FAT Atypical Cadherin 4	21303848	AR
Fgf10	Fibroblast Growth Factor 10	11062007	AR
Fgf7	Fibroblast Growth Factor 7	9876183	AR
Fgf8	Fibroblast Growth Factor 8	16049111	AR
Fgfr2	Fibroblast Growth Factor Receptor 2	15843416	AR
Fgfrl1	Fibroblast Growth Factor Receptor-Like 1	19715689	AR
Fmn1	Formin 1	7517224	AR
Foxc1	Forkhead Box C1	5500588	AD/AR
Foxd1	Forkhead Box D1	8666231	AR

Foxd2	Forkhead Box D2	10648626	AR
Foxg1	Forkhead Box G1	16109771	AD/AR
Fras1	Fraser Extracellular Matrix Complex Subu	12766769	AR
Frem1	FRAS1 Related Extracellular Matrix 1	12766769	AR
Frem2	FRAS1 Related Extracellular Matrix Prote	12766769	AR(h)
Fstl1	Follistatin Like 1	22485132	AR
Fzd4	Frizzled Class Receptor 4	21343368	AR
Fzd8	Frizzled Class Receptor 8	21343368	AR/Digenic
Gata2	GATA Binding Protein 2	18233958	AR
Gata3	GATA Binding Protein 3	16319112	AD(H)Hom Lethal
Gdf11	Growth Differentiation Factor 11	12729564	AD
Gdnf	Glial Cell Derived Neurotrophic Factor	11422733	AD
Gen1	GEN1 Holliday Junction 5' Flap Endonucl	32226308	AD
Gfra1	GDNF Family Receptor Alpha 1	23542432	AD
Glce	Glucuronic Acid Epimerase	12788935	AR
Gli3	GLI Family Zinc Finger 3	11978771	AD/AR
Gpc3	Glypican 3	10402475	XR(h)
Grem1	Gremlin 1, DAN Family BMP Antagonist	15201225	AD(null)
Grip1	Glutamate Receptor Interacting Protein 1	10974668	AR(h)
Hnf1b	HNF1 Homeobox B	23362348	AD(h)
Hoxa11	Homeobox A11	7596412	Hoxd11
Hoxd11	Homeobox D11	12050119	?
Hoxa13	Homeobox A13	12783783	AD
Hoxc10	Homeobox C10	19623272	AD
Hoxc11	Homeobox C11	12050119	Hoxa11
Hpse2	Heparanase 2	25510506	AR(h)
Hs2st1	Heparan Sulfate 2-O-Sulfotransferase 1	9637690	AR
Hsd17b2	Hydroxysteroid 17-Beta Dehydrogenase 2	18048640	?
Hspa4l	Heat Shock Protein Family A (Hsp70) Me	16923965	?
Htr3a	5-Hydroxytryptamine Receptor 3A	15201326	AD
Id2	Inhibitor Of DNA Binding 2, HLH Protein	15569159	AD
Ilk	Integrin Linked Kinase	19829382	?
Itga3	Integrin Subunit Alpha 3	10433923	AR
Itga6	Integrin Subunit Alpha 6	10433923	AR
Itga8	Integrin Subunit Alpha 8	9054500 17537792	AR
Itgb1	Integrin Subunit Beta 1	19439520	AR
Kif26b	Kinesin Family Member 26B	20439720	AR
Lama5	Laminin Subunit Alpha 5	10625553	AR
Lamc1	Laminin Subunit Gamma 1	12015298	AR
Lgr4	Leucine Rich Repeat Containing G Protei	21523854 22738954	?
Lhx1	Luteinizing Hormone/Choriogonado-tropir	16216236	?(hom lethal)
LIFR	leukemia inhibitory factor receptor	· 32179912	AD
Lin7c	Lin-7 Homolog C, Crumbs Cell Polarity Co	17923534	?(KO)
Lrp4	LDL Receptor Related Protein 4	20454682	AR
Lzts2	Leucine Zipper Tumor Suppressor 2	21949185	?
Megf8	Multiple EGF Like Domains 8	18043505	H:AR
Mmp14	Matrix Metallopeptidase 14	20727881	?
Mmp17	Matrix Metallopeptidase 17	21347258	?
Mycn	V-Myc Avian Myelocytomatosis Viral Onco	1459449	AD
Ndst1	N-Deacetylase And N-Sulfotransferase 1		?
Nf1	Neurofibromin 1	7926784	AR
Nfia	Nuclear Factor I A	17530927	AD
Nmnat2	Nicotinamide Nucleotide Adenylyltransfer	23082226	AR
Nog	Noggin	18028901	?
Notch2	Notch 2	20299358	AD
Npnt	Nephronectin	17537792	AD
Osr1	Odd-Skipped Related Transciption Factor	16790474	AR
Parva	Parvin Alpha	19829382	?
Pax2	Paired Box 2	8575306	AD
Pax8	Paired Box 8	12435636	AD
Pbx1	PBX Homeobox 1	12591246	AD(h)
Pcnt	Pericentrin (kendrin)	25220058	AR(?)
Pcsk5	Proprotein Convertase Subtilisin/Kexin Ty	18519639	AR/AD

Pdgfra	Platelet Derived Growth Factor Receptor .	19217431	AR
Pds5a	PDS5 Cohesin Associated Factor A	19412548	AD
Plxnb1	Plexin B1	18799546	?
Plxnb2	Plexin B2	21035938	?
Plxnd1	Plexin D1	J:175213	?
Ppp3r1	Protein Phosphatase 3 Regulatory Subun	15057312	?
Prickle1	Prickle Planar Cell Polarity Protein 1	25190059	AR(h)
Ptch1	Patched 1	22792366	?
Pten	Phosphatase And Tensin Homolog	17540362	AD(h)
Ptprf	Protein Tyrosine Phosphatase, Receptor	19273906	?
Pygo1	Pygopus Family PHD Finger 1	17425782	AR
Pygo2	Pygopus Family PHD Finger 2	17425782	?
Rara	Retinoic Acid Receptor Alpha	9376317	AD
Rdh10	Retinol Dehydrogenase 10 (All-Trans)	21930923 17473173	?
Rere	Arginine-Glutamic Acid Dipeptide Repeats	23451234	AR
Ret	Ret Proto-Oncogene	16452504	AD(H)
Robo1	Roundabout Guidance Receptor 1	J:175213	?
Robo2	Roundabout Guidance Receptor 2	17357069	AD
Rspo2	R-Spondin 2	17904116 12782276	
Sall1	Spalt Like Transcription Factor 1	11688560	AD
Sall4	Spalt Like Transcription Factor 4	17216607	AD
Sc5d	Sterol-C5-Desaturase	J:175213	AR(h)
Scarb2	Scavenger Receptor Class B Member 2	12620969	AR(h)
Sema3a	Semaphorin 3A	18249526	AD
Sestd1	SEC14 And Spectrin Domain Containing	23696638	?
Shh	Sonic Hedgehog	12399320	?
Six1	SIX Homeobox 1	14695375	AD
Six2	SIX Homeobox 2	17036046	AD
Slit2	Slit Guidance Ligand 2	15130495	AD
Slit3	Slit Guidance Ligand 3	14550534	?
Sox4	SRY-Box 4	16109771	AD
Sox9	SRY-Box 9	20881014	AD(h)
Spry1	Sprouty RTK Signaling Antagonist 1	15691764	AR
Sulf1	Sulfatase 1/ Sulfatase 2	17593974	Sulf1+Sulf2
Sulf2	Sulfatase 1/ Sulfatase 2	17593974	Sulf1+Sulf2
Tbx18	T-Box 18	24016759	AD
Tbx6	T-Box 6	4073528	AR/AD(H)
Tcf21	Transcription Factor 21	10572052	AR
Tfcp2l1	Transcription Factor CP2-Like 1	17079272	AR
Tgfb2	Transforming Growth Factor Beta 2	9217007	AR
TNS1	Tensin 1	23095816	
Trp53	Transformation related protein 53	11780111	?
Trps1	Transcriptional Repressor GATA Binding	19820125	?(null)
Tshz3	Teashirt Zinc Finger Homeobox 3	18776146	AD
Tyr	Tyrosinase	J:179802	?(hom)
Umod	Uromodulin	15611339	AD
Upk3a	Uroplakin 3A	11085999	AD(h)
Wasl	WAS/WASL Interacting Protein Family Me	23555292	
Wnt11	Wnt Family Member 11	12783789	
Wnt4	Wnt Family Member 4	7990960	AD
Wnt5a	Wnt Family Member 5A	J:175213	AD
Wnt7b	Wnt Family Member 7B	19060336	?(null)
Wnt9b	Wnt Family Member 9B	16054034	?
Wt1	Wilms Tumor 1	18040647	AD
Xpl	X-linked polydactyly	7391545	XLD(h)
Yap1	Yes Associated Protein 1	23555292	?
Zbtb14	Zinc Finger And BTB Domain Containing	J:175213	?
Cc2d2a	Coiled-Coil And C2 Domain Containing 2A	J:175213	AR(h)
Dync2h1	Dynein Cytoplasmic 2 Heavy Chain 1	J:175213	AR(h)
Mks1	Meckel Syndrome, Type 1	21045211	H:AR
Tbc1d32	TBC1 Domain Family Member 32	J:175213	(hom)?
Wdpcp	WD Repeat Containing Planar Cell Polari	24302887	?

Supplementary Table S4. Allele frequency of variants associated with drug pharmacogenetics in the 226 patients with CKD from the families in waiting list of transplantation

Gene	Variants	Genotype	[a]Frequency, N(%)	[b]gnomAD in EA, N(%)	[c]HWE P value	Genetic determinant of immunosuppressant	Reference
SLCO1B3	*rs4149117*	GG	138(61.1)	5235(52.5)	0.717	MMF	Eur J Pharm Sci. 2020 Jul 1;150 105370.
		GT	76(33.6)	3983(40.0)			*rs4149117_G* associated with higher dose-normalized MMF
		TT	12(5.3)	248(7.5)			
SLCO1B3	*rs7311358*	AA	138(61.1)	5228(52.5)	0.717	MMF	Eur J Clin Pharmacol. 2007 Dec;63(12):1161-9.
		AG	76(33.6)	3985(40.0)			*rs7311358_A* associated with higher dose-normalized MMF
		GG	12(5.3)	748(7.5)			
UGT1A8	*rs7439366*	CC	80(35.4)	4885(49.4)	0.969	MMF	J Clin Pharmacol. 2010 Nov;50(11):1280-91.
		CT	102(45.1)	4147(42.0)			*rs7439366_C* associated with higher dose-normalized MMF
		TT	44(19.5)	865(8.6)			
CYP3A5	*rs4646453*	AA	24(10.6)	51(6.6)	0.534	Tac	Curr Drug Metab. 2019;20(7):609-618.
		AC	93(41.2)	303(39.0)			*rs4646453_A* associated with higher dose-normalized tacrolimus
		CC	109(48.2	422(54.4)			
CYP3A5	*rs776746*	CC	33(14.6)	62(8.0)	0.210	Tac	Pharmacogenomics J. 2015 Feb;15(1):38-48
		CT	95(42.0)	320(41.3)			*rs776746_C* associated with lower dose-normalized tacrolimus
		TT	9(43.4)	393(50.7)			
CYP3A5	*rs15524*	AA	29(12.8)	788(8.0)	0.925	Tac	Pharmacogenomics J. 2015 Feb;15(1):38-48
		AG	103(45.6)	4075(41.3)			*rs15524_A* associated with lower dose-normalized tacrolimus
		GG	94(41.6)	4994(50.7)			
CYP3A7	*rs10211*	TT	98(43.4)	388(50.2)	0.945	Tac	Sci Rep. 2018 Dec 24;8(1):18064.
		CT	102(45.1)	323(41.8)			*rs10211_T* associated with higher dose-normalized tacrolimus
		CC	26(11.5)	62(8.0)			
CYP3A7	*rs12360*	AA	98(43.4)	5153(51.8)	0.945	Tac	Sci Rep. 2018 Dec 24;8(1):18064.
		AG	102(45.1)	4041(40.7)			*rs12360_A* associated with higher dose-normalized tacrolimus
		GG	26(11.5)	747(7.5)			
CYP3A7	*rs2257401*	GG	98(43.4)	5189(52.1)	0.945	Tac	Sci Rep. 2018 Dec 24;8(1):18064.
		CG	102(45.1)	4033(40.5)			*rs2257401_G* associated with higher dose-normalized tacrolimus
		CC	26(11.5)	735(7.4)			
CYP3A4	*rs2242480*	TT	13(5.8)	664(6.7)	0.131	Tac	Pharmacogenet Genomics. 2016 Oct;26(10):462-72.
		CT	100(44.2)	3910(39.5)			*rs2242480_T* associated with higher dose-normalized tacrolimus
		CC	113(50.0)	5329(53.8)			
NR1I2	*rs6785049*	AA	43(19.0)	141(18.2)	0.921	Tac	Pharmacogenet Genomics. 2017 Oct;27(10):372-377.
		AG	112(49.6)	366(47.3)			*rs6785049_A* associated with higher dose-normalized tacrolimus
		GG	71(31.4)	267(34.5)			
NR1I2	*rs2276707*	TT	55(24.3)	2166(21.7)	0.124	Tac	Pharmacogenet Genomics. 2017 Oct;27(10):372-377.
		CT	101(44.7)	4969(49.9)			*rs2276707_T* associated with higher dose-normalized tacrolimus
		CC	70(31.0)	2835(28.4)			
SUMO4	*rs237025*	AA	117(51.8)	5141(51.6)	0.958	Tac	Liver Int. 2018 Apr;38(4):724-732.
		AG	91(40.3)	4032(40.4)			*rs237025_A* associated with lower dose-normalized tacrolimus
		GG	18(8.0)	802(8.0)			
ABCB1	*rs2032582*	CC	74(32.7)	2278(22.9)	0.712	Tac	Pharmacogenet Genomics. 2016;26(10):462-72.
		AC	113(50.0)	4936(49.5)			*rs2032582_C* associated with higher dose-normalized tacrolimus
		AA	39(17.3)	2747(27.6)			
CPSF1	*rs883403*	CC	36(15.9)	1090(11.0)	0.773	Tac	Sci Rep. 2018 Dec 24;8(1):18064.
		CT	111(49.1)	4479(45.0)			*rs883403_C* associated with lower dose-normalized tacrolimus
		TT	79(35.0)	4385(44.0)			
ZNF789	*rs6962772*	GG	36(15.9)	1104(11.1)	0.773	Tac	Sci Rep. 2018 Dec 24;8(1):18064.
		AG	111(49.1)	4494(45.1)			*rs6962772_G* associated with lower dose-normalized tacrolimus
		AA	79(35.0)	4373(43.8)			
FAM200A	*rs10238965*	TT	36(15.9)	988(10.8)	0.609	Tac	Sci Rep. 2018 Dec 24;8(1):18064.
		CT	113(50.0)	4124(45.2)			*rs10238965_T* associated with higher dose-normalized tacrolimus
		CC	77(34.1)	4012(44.0)			
ZSCAN25	*rs1859690*	GG	29(12.8)	867(8.7)	0.993	Tac	Sci Rep. 2018 Dec 24;8(1):18064.
		GA	104(46.0)	4211(42.2)			*rs1859690_G* associated with lower dose-normalized tacrolimus
		AA	93(41.2)	4898(49.1)			

[a] Frequency is given as the number of the patients, with the percentage of patient cohort; [b] Frequency is given as the number of the individuals with the percentage of individuals from eastern Asian reported in gnomAD; [c] HWE, Hardy-Weinberg equilibrium

Provinces /Municipalities	Number of dialysis centers	Number of families
Henan	32	56
Shanghai	1	3
Tianjin	2	3
Beijing	1	3
Hebei	1	3
Shandong	2	9
Shaanxi	3	5
Zhejiang	4	5
Jiangsu	2	2
Fujian	2	3
Hubei	2	4
Shanxi	1	1
Heilongjiang	1	2
Nei Mongol	0	1
Gansu	1	1
Liaoning	2	2
Guangdong	5	8
Anhui	2	4
Total	64	115

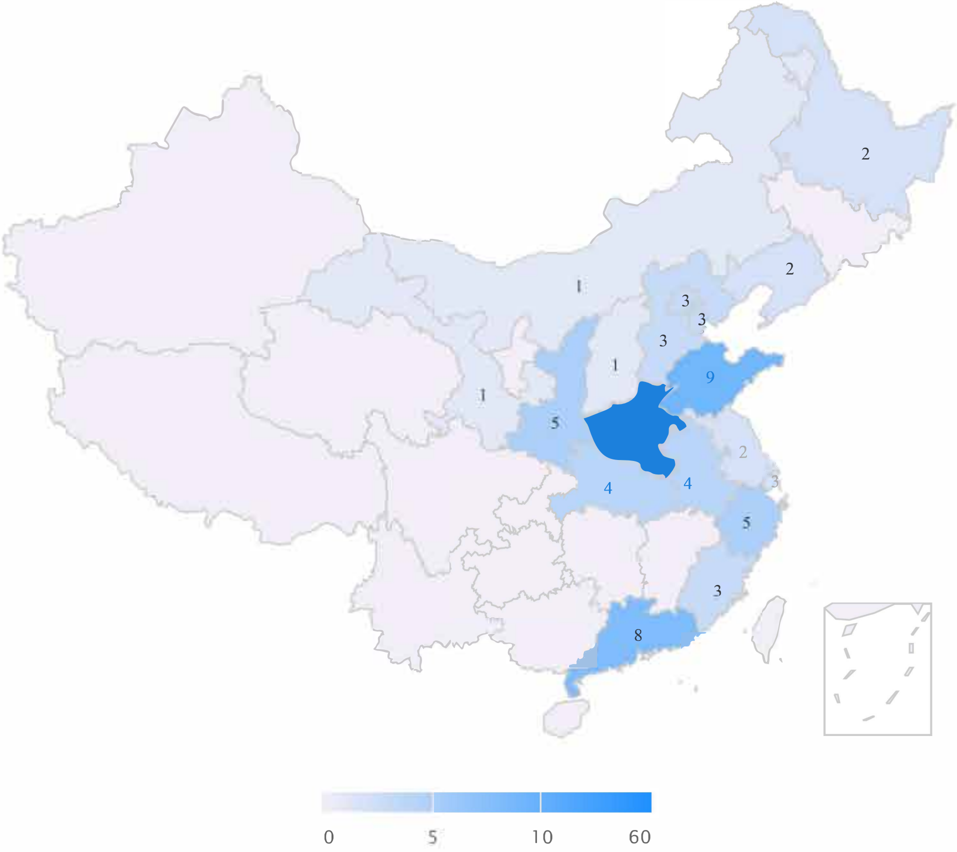

Supplementary Figure S5. Distribution of the dialysis centers with patients in the wailing list for transplantation

Supplementary Table S6. Identifying mutations in 62 families of ESRD post exome sequecing study.

Family	A priori clinical Diagnosis	Modified Dx	Post ES Dx	Extrarenal Manifestations	Family History	Knonw Disease causing gene	Inherited pattern	c.Change; p.Change; Segregation (p,m)[a]	gnomAD (All)[b]	gnomAD (EA)[b]	HGMD; ACMG Category [c]	SIFT	Polyphen 2_HDIV	Polyphen 2_HVAR	MutationTaster	CADD	ClinPred
100004	SRNS	Diagnosis correction	Alport syndrome	Hearing lost	Y,3	***COL4A5***	XL	NM_000495.4:c.4944G>A:p.W1648X; (Hemi; p,wt;m,het;sibling,hemi)	none	none	N;P(PVS1,PM1,PM2,PP3)				D(1)		
100011	GN	Precise diagnosis	Alport syndrome	None	Y,6	***COL4A3***	AD	NM_000091.4:c.697G>A,p.Gly233Arg;(het;p,wt;sibling02,het;sibling04,het;sibling05,wt;sibling09,wt;offspring23,het;offspring24,wt;offspring25,het)		none	N;LP(PM1,PM2,PM5,PP3)	D(0.0)	P(1.0)	P(1.0)	D(1.000)	D(33)	P(0.99954849)
100042	ESRDu	Diagnosis correction	NPHP	None	Y,2	***NPHP3***	AR	NM_153240.5:c.3757C>G;p.Leu1253Val;(HOM;p,het;m,het; sibling,HOM)	0.00002786(7/0/251268)	0.0001631(3/0/18394)	DM, LP(PM1,PM2, PP1, PP3, PP4)	D(0.006)	P(0.784)	P(0.676)	D(1)	D(24.0)	B(0.32238575
100046	SRNS	**Precise diagnosis**	CoQ8B nephropathy	None	N,1	***COQ8B***	AR	NM_024876.3:c.893+2T>A;(het;p,wt;m,het)	0.00001199(3/0/250162)	0.0001632(3/0/18380)	N; P(PVS1,PM2,PP1, PP3, PP4						
							AR	NM_024876.3:c.1035+3A>G;(het;p,wt;m,wt)	0.00003912(11/0/281218)	None	N; LP(PVS1,PM2,PP1, PP3, PP4)						
100054	GN	Diagnosis correction	Fabry disease	Hearing lost	Y,2	***GLA***	AD	NM_000169.2:c.878C>T:p.Pro293Leu	none	none	DM; LP(PM1,PM2,PP2,PP3,PP4)	D(0.0)	P(1.0)	P(1.0)	D(1)	D(32)	P(0.99998652)
100059	GN	Reclassification of tl	FSGS	Hearing lost	Y,2	***PAX2***	AD	NM_003990.4:c.148C>T:p.Arg50Trp;(het;m,wt;sibling02,het;sibling03,het)	none	none	N;LP(PM1,PM2,PP1,PP2,PP3)	D(0.0)	P(1.0)	P(1.0)	D(1)	D(33)	P(0.99858093)
100062	GN	Reclassification of tl	FSGS	None	Y,3	***PAX2***	AD	NM_003990.4:c.221_226dupAGACCG;p.Glu74_Thr75dup;(het;p,wt;m,wt)	none	none	DM; LP(PM1,PM2,PM4,PP3)						
100063	GN	Precise diagnosis	Alport syndrome	None	Y,2	***COL4A5***	XLR	NM_000495.4:c.2146G>A,p.Gly716Ser (hemi;p,wt;m,het;sibling,hemi)	none	none	N; P(PVS1,PM1,PM2,PP1,PP3)	D(0.007)	P(1.0)	P(1.0)	D(1)	D(25.2)	P(0.99852448)
100073	GN	**Precise diagnosis**	FSGS	None	N,1	***WT1***	AD	NM_024426.6:c.1534C>T,p.Gln512X (het; p,wt)	none	none	N; LP(PVS1,PM2,PP3)						
100074	SRNS	Diagnosis correction	Alport syndrome	Hearing lost	Y,2	***COL4A5***	XL	NM_000495.4:c.2288G>A;p.G763E;(hemi;p,wt)	none	none	DM;LP(PM1,PM2,PM5,PP1,PP2,PP3)	D(0.002)	P(1.0)	P(1.0)	D(1)	D(24.9)	P(0.99958783)
100075	SRNS	**Precise diagnosis**	**FSGS**	None	Y,2	***NUP160***	AR	NM_015231.2:c.4250_4251delAG,p.Glu1417Glyfs*7(het;p,wt; m,het)	none	None	N,P(PVS1,PM2,PP3)						
								NM_015231.2:c.2407G>A:p.Glu803Lys(het;p,het;m,wt)	0.00001592(4/0/251206)	None	DM,LP(PM2,PP1,PP2, PP3, PP4)	T(0.668)	B(0.061)	B(0.045)	D(0.996)	D(23.2)	B(0.20869764)
100076	GN	Precise diagnosis	Alport syndrome	Hearing lost	Y,2	***COL4A5***	XLR	NM_000495.4:c.1033-2A>G(hemi;p,wt;m,het)	none	none	N;P(PVS1,PM2,PP3)						
100078	CAKUT	Precise diagnosis	CAKUT	Hearing loss	Y,2	***ITGB4***	AD	NM_000213.5:c.2524C>T;p.Gln842X;(het;m,wt;s,het)	0.000007990(2/0/250300)	0.00005442(1/0/18374)	DM;P(PVS1;PM2;PP3)				D(1)	D(37)	
100080	GN	Precise diagnosis	Alport syndrome	Hearing loss	Y,2	***COL4A5***	XL	NM_000495.4:c.937G>T;p.Gly313Cys;(hemi;p,wt;m,het)	none	none	N;LP(PM1,PM2,PP2,PP3)	D(0.0)	P(1.0)	P(1.0)	D(1)	D(24.9)	P(0.99979919)
100092	SRNS	Diagnosis correction	Alport syndrome	Hearing loss	Y,3	***COL4A5***	XL	NM_000495.4:c.439-1G>A;(hemi;p,wt;m,het)	none	none	DM;P(PVS1,PM2,PP3)						
100093	GN	Precise diagnosis	Alport syndrome	visual impairment	Y,2	***COL4A5***	XL	NM_000495.4:c.2330G>C;p.Arg777Pro;(hemi;p,wt;m,het)	none	none	N;LP(PM1,PM2,PP1,PP2,PP4)	T(0.33)	B(0.002)	B(0.006)	D(0.815)	D(23.8)	P(0.80960875)
100095	GN	Precise diagnosis	Alport syndrome	Hearing loss,Blurred vision	Y,3	***COL4A5***	XL	NM_000495.4:c.1930G>T;p.Gly644Cys(het;p,hemi;m,wt)	none	none	N;LP(PM1,PM2,PP1,PP3,PP4)	D(0.002)	P(1.0)	P(1.0)	D(1)	D(25.5)	P(0.99966228)
100098	SRNS	Diagnosis correction	Alport syndrome	Hearing loss	Y,2	***COL4A3***	AD	NM_000091.4:c.600_603dupGGGA;p.Phe202Glyfs*12;(het;p,het;m,wt)	none	none	N;P(PVS1,PM1,PM2,PP1,PP3)						
100107	GN	Precise diagnosis	Alport syndrome	Hearing loss	Y,3	***COL4A5***	XL	NM_000495.4:c.1480G>T;p.Gly494Cys;(het;p,hemi;m,wt)	none	none	N;P(PM1,PM2,PM5,PP2,PP3)	D(0.001)	P(1.0)	P(1.0)	D(1)	D(24.8)	P(0.99980384)
100112	SRNS	Diagnosis correction	Alport syndrome	None	Y,2	***COL4A5***	XL	NM_000495.4:c.584G>A;p.Gly195Asp;(hemi;p,wt;m,het)	none	none	DM;P(PM1,PM2,PM5,PP2,PP3)	D(0.002)	P(1.0)	P(1.0)	D(1)	D(24.8)	P(0.99916028)
100113	SRNS	Diagnosis correction	Alport syndrome	None	Y,2	***COL4A5***	XL	NM_000495.4:c.2237G>A;p.Gly746Glu;(hemi;p,wt;m,het)	none	none	DM;LP(PM1,PM2,PP2,PP3)	D(0.001)	P(1.0)	P(1.0)	D(1)	D(23.6)	P(0.999574)
100115	SRNS	Diagnosis correction	Alport syndrome	Hearing loss	Y,2	***COL4A5***	XL	NM_000495.4:c.3659G>A;p.Gly1220Asp;(het;p,wt;m,het)	none	none	DM;LP(PM1,PM2,PM5,PP1,PP2,PP3,PP4)	D(0.003)	P(0.996)	P(0.885)	D(1)	D(25.1)	P(0.99816966)
100118	SRNS	**Precise diagnosis**	**FSGS**	Hearing loss,high myopia	Y,2	***TRPC6***	AD	NM_004621.6:c.1394T>C;p.Phe465Ser;(het;p,wt;m,het)	none	none	N;LP(PM2,PP1,PP3,PP4,PP5)	T(0.256)	P(0.987)	P(0.88)	D(1)	D(29.2)	P(0.99800461)
100119	GN	Precise diagnosis	Alport syndrome	tinnitus	Y,2	***COL4A5***	XL	NM_000495.4:c.4510G>C;p.Gly1504Arg;(hemi;p,wt;m,het)	none	none	N; P(PVS1,PM1,PM2,PP2,PP3)	D(0.0)	P(1.0)	P(1.0)	D(1)	D(32)	P(0.99963581)
100122	TK	Diagnosis correction	Renal colobma syn	None	Y,3	***PAX2***	AD	NM_003990.4:c.1127A>C;p.Gln376Pro;(het;p,het;m,wt;)	0.0001709(48/0/280814)	0.002362(47/0/19896)	N;LP (PM1,PP1,PP2,PP3,PP4)	T(0.114)	P(0.99)	P(0.983)	D(1.000)	D(24.9)	B(0.26589816)
100123	GN	Diagnosis correction	Lipoprotein nephropathy	None	Y,2	***APOE***	AD	NM_000041.4:c.127C>T;p.Arg43Cys;(het??)	0.000007988(2/0/250366)	0.00005445(1/0/18364)	DM,LP(PM2,PP1,PP2,PP3,PP4)	D(0.017)	P(0.678)	B(0.105)	D(0.988)	D(24.2)	B(0.31882999)
100127	CAKUT	Diagnosis correction	ADTKD	None	Y,3	***UMOD***	AD	NM_001008389.2:c.203A>T;p.Glu68Val;(het;m,wt;sibling06,het;sibling07,wt;sibling12,het;sibling16,wt)	none	none	N;LP(PM1,PM2,PP2,PP3)	T(0.161)	P(1.0)	P(0.999)	D(0.999)	D(26.6)	P(0.99402987)
100128	SRNS	Diagnosis correction	Alport syndrome	Hearing loss,Blurr	Y,3	***COL4A5***	XL	NM_000495.4:c.2597G>A;p.Gly866Glu;(hemi;p,wt;m,het)	none	none	DM;LP(PM1,PM2,PP2,PP3,PP4)	D(0.0)	P(1.0)	P(1.0)	D(1)	D(25.0)	P(0.99966371)
100130	SRNS	Diagnosis correction	Alport syndrome	Hearing loss	Y,2	***COL4A5***	XL	NM_000495.4:c.4960_4961insAAAA;p.Val1654Glufs*8;(het;p,wt;m,het)	none	none	N;P(PVS1,PM1,PM2)						
100131	SRNS	Diagnosis correction	Alport syndrome	Hearing loss,high	Y,2	***COL4A3***	AR	NM_000091.4:c.1865G>A;p.Gly622Glu;(het;p,het;m,wt)	none	none	N;LP(PM1,PM2,PP2,PP3)	D(0.0)	P(0.999)	P(0.979)	D(1.000)	D(24.0)	P(0.99894136)
							AR	NM_000091.4:c.3575G>A;p.Gly1192Glu;(het;p,wt;m,het)	none	none	DM;LP(PM1,PM2,PP2,PP3)	D(0.0)	P(1.0)	P(1.0)	D(1)	D(23.3)	P(0.99948406)
100133	SRNS	Diagnosis correction	Alport syndrome	None	Y,2	***COL4A4***	AR	NM_000092.4:c.2726G>A;p.Gly909Glu;(het;p,het;m,wt)	none	none	DM;LP(PM1,PM2,PP1,PP3)	D(0.002)	P(1.0)	P(1.0)	D(1)	D(25.7)	P(0.99881279)
							AR	NM_000092.4:c.1459+5G>A;(het;p,wt;m,het)	none	none	N;P(PVS1,PM2,PP1,PP3)						
100135	GN	Precise diagnosis	Alport syndrome	None	Y,3	***COL4A5***	XL	NM_000495.4:c.4688+2T>C;(het;m,het)	none	none	N;P(PVS1,PM2,PP3)						
100137	SRNS	**Diagnosis correctio**	Lipoprotein nephropathy	visual impairment	Y,6	***APOE***		NM_000041.4:c.494G>C:p.Arg165Pro(het;p,het;m,wt;uncle07,het;aunt10,het;aunt12,het;wife05,wt;offspring06,het;grandmother01,het)	none	none	N;LP(PM1,PM2,PP2,PP3,PP4)	D(0.0)	P(1.0)	P(1.0)	D(1.000)	D(32)	P(0.99293875)

100140	GN	Precise diagnosis	Alport syndrome	None	Y,5	***COL4A5***	XL	NM_000495.4:c.476G>A;p.Gly159Asp;(hemi;p,wt;m,het)	none	none	N;LP(PM1,PM2,PP1,PP2,PP3)	D(0.0)	P(1.0)	P(1.0)	D(1)	D(24.8)	P(0.99925869)
100141	SRNS	**Precise diagnosis**	**FSGS**	None	Y,4	***TRPC6***	AD	NM_004621.6:c.644G>A;p.Arg215Gln;(het;p,wt;m,het;sibling03.het;sibling04.het;sibling05.het)	none	none	N;LP(PM2,PP1,PP2,PP3,PP4)	T(0.079)	P(1.0)	P(0.999)	D(1)	D(27.9)	P(0.97448414)
100145	GN	Precise diagnosis	Alport syndrome	None	Y,3	***COL4A5***	XL	NM_000495.4:c.3790+1724G>T;het(m,het)	none	none	N;LP(PM1,PM2,PP2,PP3)						
100146	GN	Precise diagnosis	Alport syndrome	Hearing loss	Y,2	***COL4A5***	XL	NM_000495.4:c.3670G>T;p.Glu1224X;hemi(p,wt;m,het)	none	none	DM;P(PVS1,PM1,PM2,PP3,PP4)				D(1)		
100148	CAKUT	**Diagnosis correcti**	NPHP	None	Y,2	***TTC21B***	AR	NM_024753.5:c.1552T>C;p.Cys518Arg;(het;p,het;m,wt)	none	none	DM;LP(PM2,PP1,PP3,PP4,PP5)	D(0.001)	P(0.981)	P(0.963)	D(1)	D(24.8)	P(0.99962484)
							AR	NM_024753.5:c.497delA;p.Lys166SerfsX36;(het;p,wt;m,het)	none	none	N, P(PVS1,PM2,PP1,PP3)						
100150	SRNS	Reclassification of t	**UMOD associated FSGS**	visual impairment	Y,2	***UMOD***	AD	NM_001008389.3:c.1196A>G;p.His399Arg;het(p,wt;m,het)	none	none	N;LP(PM1,PM2,PP1,PP2,PP3)	D(0.034)	P(1.0)	P(0.998)	D(0.983)	D(23.4)	P(0.98754233)
100152	SRNS	Diagnosis correction	Alport syndrome	None	Y,2	***COL4A5***	XL	NM_000495.4:c.1871G>T;p.Gly624Val;hemi(p,wt;m,het)	none	none	N;LP(PM1,PM2,PM5,PP2,PP3)	D(0.009)	P(0.999)	P(0.949)	D(1)	D(23.6)	P(0.993887)
100154	SRNS	**Precise diagnosis**	CoQ8B nephropathy	None	Y,2	***COQ8B***	AR	NM_024876.4:c.737G>A;p.Ser246Asn;(HOM; p,het;m,het)	0.00006401(18/0/281206)	0.0009023(18/0/19948)	DM;LP(PM1,PM2,PP1,PP3)	D(0.002)	P(1.0)	P(0.995)	D(1)	D(32)	B(0.42169388)
100156	GN	Precise diagnosis	Alport syndrome	None	Y,2	***COL4A5***	XL	NM_000495.4:c.539G>A;p.Gly180Glu;hemi(p,wt)	none	none	DM;LP(PM1,PM2,PM5,PP2,PP3)	D(0.001)	P(1.0)	P(1.0)	D(1)	D(23.2)	P(0.99884676)
100158	GN	Precise diagnosis	Alport syndrome	None	Y,2	***COL4A5***	XL	NM_000495.4:c.3940C>T;p.Pro1314Ser;(hemi;p,wt;m,het)	0.0003128(64/0/204636)	0.004257(63/0/14798)	DM;LP(PS1,PM1,PP2,PP3)	T(0.088)	P(0.606)	B(0.212)	D(1.000)	D(25.7)	B(0.15954074)
100159	SRNS	**Precise diagnosis**	**FSGS**	None	N,1	***NPHS2***	AR	NM_014625.4:c.547G>T;p.Asp183Tyr;(het;p,het;m,wt)	none	none	N;LP(PM2,PP1,PP2,PP3,PP4)	D(0.0)	P(1.0)	P(1.0)	D(1)	D(34)	P(0.99951052)
							AR	NM_014625.4:c.686G>A;p.Arg229Gln;(het;p,wt;m,het)	0.03025(8538/186/282294)	0.0001003(2/0/19942)	DM;LP(PM1,PP1,PP2,PP3,PP4)	T(0.21)	P(0.903)	B(0.313)	D(1)	D(28.2)	B(0.03039302)
100164	SRNS	**Precise diagnosis**	CoQ8B nephropathy		N,1	***COQ8B***	AR	NM_024876.4:c.1430G>A;p.Arg477Gln;(het;p,wt;m,het)	0.000004133(1/0/241932)	none	DM;LP(PM2,PP1,PP3,PP4,PP5)	D(0.0)	P(1.0)	P(1.0)	D(1)	D(34)	P(0.9882759)
								NM_024876.4:c.748G>C;p.Asp250His;(het;p,het;m,wt)	0.00005344(15/0/280686)	0.0007523(15/0/19938)	DM;LP(PM1, PP1,PP3,PP4,PP5)	D(0.0)	P(1.0)	P(1.0)	D(1)	D(29.5)	P(0.97774112)
100193	GN	Precise diagnosis	Alport syndrome	None	Y,2	***COL4A5***	XL	NM_000495.4:c.322-1G>T;hemi(m,het)	none	none	N;P(PVS1,PM2,PP1,PP3)						
100207	GN	Precise diagnosis	Alport syndrome	Hearing loss,myopia	Y,2	***COL4A5***	XL	NM_000495.4:c.4769G>A;p.Trp1590X;het(m,het)	none	none	N;P(PVS1,PM2,PP3)				D(1)		
100212	TK	Precise diagnosis	NPHP	None	Y,2	***TMEM67***	AR	NM_153704.6:c.520G>C;p.Glu174Gln;(het;m,wt)	none	none	N;LP(PM1,PM2,PP1,PP3,PP4)	T(0.221)	B(0.203)	B(0.085)	D(0.992)	T(19.68)	P(0.70018416)
							AR	NM_153704.6:c.224-3delT;(het;m,het)	none	none	N;LP(PVS1,PM2)						
100215	GN	Precise diagnosis	Alport syndrome	None	Y,2	***COL4A3***	AD	NM_000091.4:c.2267G>A;p.Gly756Asp;het(p,wt;m,het)	none	none	N;LP(PM1,PM2,PP2,PP3)	D(0.0)	P(1.0)	P(1.0)	D(1)	D(24.6)	P(0.99961566)
100232	GN	Precise diagnosis	Alport syndrome	None	Y,2	***COL4A5***	XL	NM_000495.4:c.3311G>A;p.Gly1104Asp;hemi(p,wt)	none	none	N;LP(PM1,PM2,PM5,PP2,PP3)	D(0.002)	P(1.0)	P(1.0)	D(1)	D(23.7)	P(0.99904173)
100234	GN	Precise diagnosis	Alport syndrome	None	Y,2	***COL4A3***	AR	NM_000091.4:c.3769G>A;p.G1257R;(het;p,het;m,wt)	none	none	DM;LP(PM1,PM2,PP1,PP3,PP4)	D(0.002)	P(1.0)	P(1.0)	D(1)	D(24.5)	P(0.99964177)
							AR	NM_000091.4:c.4793T>G;p.L1598R;(het;p,wt;m,het)	0.00005341(15/0/280832)	0.0007678(15/0/19536)	DM;LP(PM1,PP1,PP2,PP3,PP4,PP5)	D(0.0)	P(1.0)	P(1.0)	D(1)	D(25.1)	P(0.75956451)
100235	SRNS	Diagnosis correction	Alport syndrome	high myopia	Y,2	***COL4A3***	AD	NM_000091.4:c.2990G>A;p.Gly997Glu;(HOM;p,het;m,het)	none	none	DM;LP(PM1,PM2,PM3,PP3,PP4)	D(0.0)	P(1.0)	P(1.0)	D(1)	D(24.7)	P(0.99938869)
100240	SRNS	Diagnosis correction	Alport syndrome	None	Y,3	***COL4A3***	AR	NM_000091.4:c.1855G>A;p.Gly619Arg;het(p,wt;m,het)	none	none	DM;LP(PM1,PM2,PP1,PP3)	D(0.002)	P(1.0)	P(1.0)	D(1.000)	D(25.9)	P(0.99776577)
							AR	NM_000091.4:c.4793T>G;p.Leu1598Arg;het(p,het;m,wt)	0.00005341(15/0/280832)	0.0007678(15/0/19536)	DM;LP(PM1,PP1,PP3,PP4,PP5)	D(0.0)	P(1.0)	P(1.0)	D(1)	D(25.1)	P(0.75956451)
100242	GN	Precise diagnosis	Alport syndrome	Hearing loss,Blurred vision	Y,3	***COL4A5***	XL	NM_000495.4:c.891+5G>A;hemi(p,wt;m,het)	none	none	N;P(PVS1,PM2,PP1,PP4)						
100243	SRNS	Diagnosis correction	Alport syndrome	None	Y,2	***COL4A5***	XL	NM_000495.4:c.670G>A;p.Gly224Arg;(het;p,hemi;m,wt)	none	none	N;LP(PM1,PM2,PP1,PP3)	D(0.001)	P(1.0)	P(0.999)	D(1)	D(23.8)	P(0.9983347)
100244	SRNS	Diagnosis correction	Alport syndrome	None	Y,2	***COL4A5***	XL	NM_000495.4:c.2395+3A>G;(hemi； p,wt;m,het)	none	none	DM;P(PVS1,PM2,PP1,PP4)						
100245	SRNS	Diagnosis correction	Alport syndrome	Hearing loss	Y,2	***COL4A5***	XL	NM_000495.4:c.687+1G>A;(hemi;m,het)	none	none	DM;P(PVS1,PM2,PP3)						
fam1002491	ESRDu	**Diagnosis correcti**	Renal colobma syn	None	N,1	***PAX2***	AD	NM_003990.4: c.239C>A;p.Pro80Gln;het	none	none	N;LP(PM1,PM2,PM5,PP3)	D(0.0)	P(1.0)	P(1.0)	D(1)	D(33)	P(0.99570953)
100250	GN	Precise diagnosis	Alport syndrome	None	Y,2	***COL4A5***	XL	NM_000495.4:c.3799G>A;p.Gly1267Ser;(hemi;m,wt)	none	none	DM;LP(PM1,PM2,PP2,PP3)	D(0.0)	P(1.0)	P(0.999)	D(1)	D(24.5)	P(0.99934774)
100253	SRNS	**Precise diagnosis**	**FSGS**	None	N,1	***PAX2***	AD	NM_003990.5:c.76dupG;p.Val26Glyfs*28;(het;sibling01,wt)	0.00002834(7/0/246960)	none	DM;P(PVS1,PM2,PP3)						
100276	GN	Precise diagnosis	Alport syndrome	None	Y,2	***COL4A5***	XL	NM_000495.5:c.1033-6A>G;(hemi;p,wt)	none	none	DM;P(PVS1,PM2,PP3)						
100041	GN	Diagnosis correction	Mitochondrial disorder	Hearing lost	Y,2	***RMND1***	AR	NM_017909.4:c.859A>T,p.Ile287Phe(HOM, s,HOM, p,het; m,het;)	none	none	N;LP(PM2,PM3, PP1, PP3, PP4)	T(0.666)	B(0.192)	B(0.229)	D(1.000)	D(24.8)	P(0.87354737)

a Impact of variant on cDNA level.; Impact of variant on the amino acid or protein level.
b gnomAD, variant frequencies listed for homozygous/ hemizygous (if applicable)/ heterozygous/ total alleles(http://gnomad.broadinstitute.org/). All, all population, EA, eastern Asian.
c HGMD, Human Gene Mutation Database(https://portal.biobaseinternational.com/hgmd). If the exact variant has been reported previously on HGMD® Professional 2017.2 for the reported phenotype and classified as a disease-causing pathogenic mutation, the variant is denoted as “DM.” The variant is denoted as “LD” if the variant is likely a disease-causing pathogenic mutation, but either the author indicated some doubt or subsequent evidence calls the deleterious nature of the variant into question. If the gene, but not the exact variant, has been reported for the corresponding phenotype, then “N” is indicated in this column.
ACMG, American College of Medical Genetics and Genomics Standards and Guidelines Classification as pathogenic, likely pathogenic or VUS (Richards Genet Med 17(5):405, 2015).

Supplementary Table S8. Secondary findings with pathogenic and likely pathogenic variants unrelated to the phenotype of the probands based on the recommended minimum list of genes selected by the ACMG committee

Family ID	Gene	HGVS nomenclature c.Change; p.Change	Inherited pattern	ACMG		Variants to report
100071	***TGFBR1***	NM_004612.4:c.343+1G>A	AD	Pathogenic	PVS1,PM2,PP3	KP and EP
100145	***BRCA2***	NM_000059.3:c.2946A>G,p.Ile982Met	AD	likely pathogenic	PM1,PM2,PM5,BP4	KP and EP
100164	***FBN1***	NM_000138.5:c.3758A>G,p.Gln1253Arg	AD	likely pathogenic	PM1,PM2,PP2,PP3	KP and EP
100215	***COL3A1***	NM_000090.4:c.3299G>A,p.Arg1100His	AD	likely pathogenic	LP(PM1,PM2,PP3,PP2)	KP and EP
100232	***DSP***	NM_004415.4:c.268C>T,p.Gln90Ter	AD	Pathogenic	P(PVS1,PM2,PP3)	KP
100235	***TSC2***	NM_000548.5:c.3814+1G>T	AD	Pathogenic	P(PVS1,PM2,PP3)	KP and EP
100243	***KCNQ1***	NM_000218.3:c.1552C>T,p.Arg518Ter	AD	likely pathogenic	LP(PVS1,PM2)	KP and EP
100067	***BRCA2***	NM_000059.3:c.4385T>A,p.Leu1462Ter	AD	Pathogenic	PVS1,PM2	KP and EP
100211	***LDLR***	NM_000527.5:c.292G>A,p.Gly98Ser	AD	likely pathogenic	PM1,PM2,PP2,PP3	KP and EP

AD, autosomal dominant; AR, autosomal recessive; c. change, nucleotide change; EP:expected pathogenic; HGSV, human genome variation society; KP:known pathogenic; p. change, amino acid change;

Supplementary Table S7. A potentially pathogenic mutations in knonw disease causitive genes or candidate genes related to kidney idnentified in 17 faimilies

Family	A priori clinical Dx	Post WES Dx	Consanguinity, Family	Age at initial diagnosis,gender	Initial clinical features	Renal Ultrasound	Renal pathological findings	Extrarenal Manifestations	Age of developing into ESRD	Gene	Inherited pattern	c.Change; p.Change; Segregation (p,m,s)	gnomAD (ALL/EA)	ACMG Category	SIFT	Polyphen2_HDIV	Polyphen2_HVAR	MutationTaster	CADD	ClinPred	Note from MDT
100034	GN	SRNS(FSGS)	N,Y,3	100034_21, 13 yr, M	non-nephrotic proteinuria	Diffuse lesions of both kidneys, reduced size of both kidneys	N.D.	None	25 yr	***LAMA5***	AR	NM_005560.6:c.9700_9728del,p.Leu3234fs;(het)	none	N;P(PVS1,PM2)							Possible genetic diagnosis of FSGS – additional evidence required to confirm or refute this result
												NM_005560.6:c.7051C>T,p.Arg2351Trp;(het)	0.00009130(25/0/273818);0.0004580(9/0/19652)	N;VUS(PP1,PP3,PP4)	D(0.0)	P(1.0)	P(0.95)	D(1.000)	D(29.3)	P(0.69959253)	
100068	GN	SRNS(FSGS)	N,Y,2	100068_21, 32 yr, M	Nephrotic proteinuria, hematuria	Diffuse lesions of both kidneys, reduced size of both kidneys	N.D.	None	37 yr	***LAMA5***	AR	NM_005560.6:c.8488G>A;p.Ala2830Thr;het(p,wt; m,het)	0.00004407(11/0/249578);0.0004897(9/0/18380)	N,VUS(PP1,PP3,PP4)	T(0.21)	B(0.002)	B(0.012)	N(0.992)	T(10.59)	B(0.01040332)	Possible genetic diagnosis of FSGS – additional evidence required to confirm or refute this result
												NM_005560.6:c.4315G>A;p.Gly1439Ser;het(p,het; m,wt)	0.00002402(6/0/249744);0.0002178(4/0/18366)	N,VUS(PP1,PP3,PP4)	T(0.297)	B(0.023)	B(0.03)	N(0.755)	D(23.2)	B(0.14545386)	
100069	GN	SRNS(FSGS)	N,Y,2	27 yr, F	Nephrotic proteinuria, hematuria	Diffuse lesions of both kidneys, reduced size of both kidneys	N.D.	None	29 yr	***LAMA5***	AR	NM_005560.6:c.5315C>T;p.Thr1772Met;het(m,het)	0.00005301(13/0/245234);0.0003825(7/0/18302)	N,VUS(PP1,PP3,PP4)	T(0.184)	B(0.329)	B(0.04)	N(1)	T(14.37)	B(0.03238507)	Possible genetic diagnosis of FSGS – additional evidence required to confirm or refute this result
												NM_005560.6:c.1538G>A;p.Gly513Glu;het(m,wt)	0.000008190(2/0/244190);0.0001106(2/0/18082)	N,VUS(PP1,PP3,PP4)	T(0.386)	P(0.896)	P(0.46)	N(0.951)	D(23)	P(0.96350067)	
100241	GN	SRNS(FSGS)	N,Y,2	18 yr, M	non-nephrotic proteinuria, hematuria	Diffuse lesions of both kidneys, reduced size of both kidneys	N.D.	None	20 yr	***LAMA5***	AR	NM_005560.6:c.8674C>T;p.Arg2892Cys;(het;p,het;m,wt)	0.00002952(8/0/270988);0.0001538(3/0/19512)	N,VUSPP1,PP3,PP4	D(0.01)	P(0.974)	P(0.664)	N(1.000)	D(25.3)	P(0.5115668)	Possible genetic diagnosis of FSGS – additional evidence required to confirm or refute this result
												NM_005560.6:c.6859C>Tp.Arg2287Cys;(het;p,wt;m,het)	0.0002728(48/1/175950);0.003275(41/1/12518)	N,VUSPP1,PP3,PP4)	D(0.011)	P(0.926)	B(0.37)	N(1.000)	D(22.4)	B(0.10872701)	
100087	SRNS	SRNS	N,Y,2	1y,F	SRNS	no abnormal	FSGS	none	6 yr	***NPHS1***	AR	NM_004646.3:c.2512C>A;p.Pro838Thr;het(p,wt;m,het)	none	DM;VUS(PM2,PP1,PP3)	D(0.0)	P(1.0)	P(1.0)	D(1.000)	D(25.8)	P(0.99948453)	Possible genetic diagnosis of FSGS – additional evidence required to confirm or refute this result
											AR	NM_004646.3:c.14C>T;p.Thr5Met;het(p,het;m,wt)	0.0002526(47/0/186058);0.003157(41/0/12988)	DM;VUS(BP4)	D(0.018)	B(0.002)	B(0.001)	N(1)	T(10.75)	B(0.02658752)	
100148	GN	NPHP	N,Y,2	100148_21,1 yr,M; 100248_22, 6 yr, F	100148_21,non-nephrotic proteinuria ; 100248_22, 6 yr,	100148_21,no abnormal 100248_22, 6 yr, mutiple	none	none	100148_21,7 yr	***TTC21B***	AR	NM_024753.5:c.3664C>T;p.Arg1222Trp;(het;p,het;m,wt)	none	N,LP(PM2,PP1,PP3,PP4)	D(0.001)	P(0.998)	P(0.614)	D(1.000)	D(34)	P(0.93935436)	Possible genetic diagnosis of FSGS – additional evidence required to confirm or refute this result
												NM_024753.5:c.256A>C;p.Asn86His;(het;p,wt;m,het)	0.0001026(29/0/282644),0.001453(29/0/19952)	N,VUS(PM2,PP3)	T(0.278)	B(0.034)	B(0.015)	D(1.000)	T(13.98)	B(0.04010099)	
100058	SRNS	SRNS	N,Y,2	100058_21,8 yr ,F 100058_11,42 yr,M	100058_21,nephrotic proteinuria;100058_22,nephrotic	100058_21 Diffuse lesions of both kidneys, reduced size of	100058_21 MsPGN	100058_21, Hearing loss	100058_21,13 yr	***SHROOM3***	AD	NM_020859.4:c.4726A>G,p.Lys1576Glu(het;p,het;m,wt)	0.0006027(169/1/280398);0.007002(139/1/19852)	VUS	T(0.165)	P(0.608)	B(0.188)	N(0.978)	T(18.49)	B(0.05094900)	Consider further investigation
100091	SRNS	SRNS	N,Y,2	13 yr, M	100091_21,none-nephrotic proteinuria; maternal	Diffuse lesions of both kidneys, reduced size of both kidneys	N.D.	None	22 yr	***VAV2***	AD	NM_001134398.2:c.2459C>T,p.Thr820Ile (het;p,het; m,wt)	none	VUS(PM1;PM2)	T(1.0)	P(0.983)	P(0.78)	D(1.0)	D(23.6)	P(0.93146055)	Consider further investigation
100147	SRNS	SRNS	N,Y,2	100147_21, 23 yr, M; 100147_11, 43 yr, M	100147_21, nephrotic proteinuria, edema;	no abnormal	100147_21, FSGS	None	100147_21, 27 yr; 100147_11, 43 yr, died of ESRD of	***VAV2***	AD	NM_001134398.2:c.961G>C:p.Gly321Arg (het;m,wt)	0.00002483(7/0/281956);0.0003510(7/0/19944)	VUS(PM1;PM2)	D(0.001)	P(1.0)	P(1.0)	D(1)	D(25.2)	P(0.82344979)	Consider further investigation
100134	SRNS	SRNS	N,Y,2	100134_21, 22 yr, F; 100134_12, 36 yr,F	100134_21, nephrotic proteinuria, edema;	no abnormal	100134_21,FSGS	none	100134_12, 41 yr	***DSCAM***	AD	NM_001389.5:c.494T>C， p.Val165Ala (het;p,wt; m,het)	none	VUS(PM1;PM2)	T(0.348)	P(0.472)	B(0.138)	D(0.732)	T(13.25)	B(0.40944957)	Consider further investigation
100134	SRNS	SRNS	N,Y,2	100136_21, 12 yr, M; 100136_12, 34 yr,F	100136_21, nephrotic proteinuria, edema;	no abnormal	100136_21,IgA nephropathy with	100136_21,amblyopia at age of 2.5 yr, hamartoma;	100136_12, 47 yr	***DSCAM***	AD	NM_001389.5:c.733C>A,p.Pro245Thr(het;p,wt;m,het)	none	VUS(PM1;PM2)	T(0.074)	P(0,999)	P(0.995)	D(1.000)	D(23.4)	P(0.99530631)	Consider further investigation
100157	TK	CAKUT	N,Y,2	35 yr, F	100157_21, higher value of serum creatinine without abnormal	Diffuse lesions of both kidneys, reduced size of both kidneys	100157_21, tubulointerstitial inflammation	None	100157_21,36 yr	***MAZ***	AD	NM_002383.4:c.1342G>A,p.Ala448Thr(het;m,wt,)	none	VUS(PM2;BP4)	T(0.869)	B(0.267)	B(0.054)	D(1)	T(2.33)	B(0.07706873)	Consider further investigation

100248	CAKUT	CAKUT	N,Y,2	38 yr, M	100248_21, higher value of serum creatinine without abnormal	Reduced size of both kidneys	N.D.	None	100248_21,39 yr;	***PLXNB2***	AR	NM_012401.4:c.2339C>T,p.Ala780Val (het); c.3136G>A, p.Val1046Met (het)	0.00001617(3/0/185556);none||0.000008129(2/0/246020);none	VUS(PM1;PM2;BP4);VUS(PM2;BP4)	T(1.0)	B(0)	B(0)	N(0.998)	T(4.838)	B(0.04518869)	Consider further investigation
1002492	ESRD unknwon	CAKUT	N,Y,3	1002492_21,44 yr,M; 1002492_22, 41,F	1002492_21, higher value of serum creatinine with non nephrotic	Diffuse lesions of both kidneys, reduced size of both kidneys	N.D.	None	10002492_21, 44 yr; 1002492_12, died of ESRD	***KDM2B***	AD	NM_032590.5 :c.1699G>C,p.Val567Leu(het)	none	VUS(PM2;PP2)	T(0.222)	B(0.034)	B(0.01)	D(1)	T(16.2)	B(0.20335993)	Consider further investigation
100251	ESRD unknwon	CAKUT	N,Y,2	100251_21, 22 yr, M; 100251_11, 45 yr, M	100251_21,higher value of serum creatinine with non nephrotic	Diffuse lesions of both kidneys, reduced size of both kidneys	N.D.	None	100251_21,22 yr; 100251_11, died of Stroke	***GLI3***	AD	NM_000168.6:c.1348G>A,p.Gly450Arg(het)	0.000003981(1/0/251198);none	VUS(PM2;BP1)	D(0.034)	B(0.084)	B(0.058)	D(0.965)	D(23.0)	B(0.47141351)	Consider further investigation
100067	ESRD unknwon	CAKUT	N,Y,2	10067_21, 3 yr, M; 10067_12, 25 yr, F	non-nephrotic range proteinuria presented in both 100067_21 and	Diffuse lesions of both kidneys, reduced size of both kidneys	N.D.	Hearring loss presented in both 100067_21	100067_21, 17 yr; 100067_12, 28 yr	***GLI2***	AD	NM_001374353.1:c.305G>A;p.Arg102Gln(het;p,wt;m,het)	0.0001274(36/0/282516); 0.0003008(6/19944)	VUS(PM2;BP1)	D(0.002)	P(1.0)	P(0.998)	D(1)	D(28.8)	B (0.33416511)	Consider further investigation
100254	CG	Danon disease	N,Y,2	100254_21:11 yr, M; 100254_22: 13 yr, M	Both non-nephrotic proteinuria	Both showed hyperechogenicity of bilateral kidney	N.D.	Dialated cardiomyopathy, no myopathy	100254_21:14 yr; 100254_22: 14 yr	***LAMP2***	XL	NM_013995.2:c.1171G>A:p.Val391Ile(hemi;s,wt)	0.003835(787/0/205198); 0.0003364(5/0/14862)	N;B(PM1;BS1;BS2;BP1;BP4)	D(0.001)	P(1.0)	P(1.0)	D(1)	D(32)	P(0.99892312)	Consider further investigation

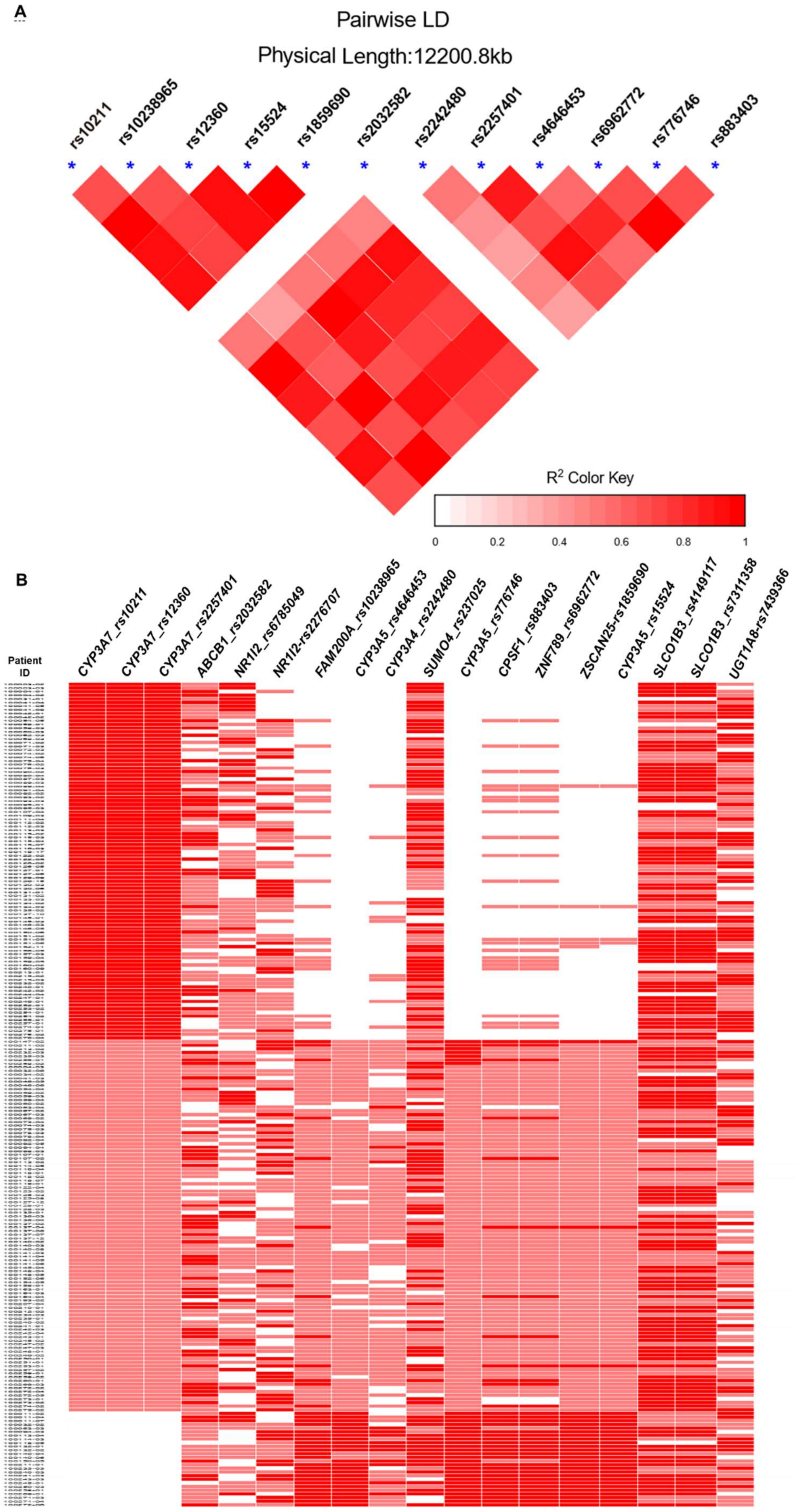

Supplementary Figure S9.

Supplementary Figure S9. Whole exome sequencing for genetic determinant of associated with tacrolimus or mycophenolate concentration in the waitlist cohort of kidney transplant.

(A) Haplotype plots of the 12 variants on chromosome 7 commonly associated with dose adjusted tacrolimus concentration. Linkage disequilibrium structures were designated by the R^2 values. Each diamond represents the correlation (R^2) between each pair of SNPs with darker shades representing stronger linkage disequilibrium. There was a high degree of LD between *CYP3A7* rs2257401 and *CYP3A5* rs776746 (R^2=0.93) LDheatmap software was used for the analysis.

(B) Heat map plots of 18 variants commonly associated with dose adjusted tacrolimus or mycophenolate concentration in 226 patients from the waitlist cohort of transplantation. The color was scaled based on genotype (red, homozygous; white, wild type)

Supplementary Section S9. The QA QC (quality control and quality assurance) report post WES

Sample	Datasize(G)	Q30	GC_content	IS_avg	IS_sd	Mapping_rate	Dup_rate	Avg	Cov1X	Cov10X	Cov20X	Cov50X	Cov100X	Uniformity	target_rate
100003-01	14.52	0.899	0.52	276.8	100.9	0.996	0.202	101	0.999	0.99	0.958	0.695	0.358	0.952	0.573
100003-02	12.93	0.89	0.51	285.1	109.6	0.995	0.232	77.6	0.998	0.987	0.943	0.604	0.239	0.967	0.7
100003-03	14.22	0.906	0.52	252.4	88.7	0.997	0.211	101.5	0.997	0.989	0.968	0.735	0.366	0.964	0.59
100003-04	14.78	0.909	0.52	244.9	87	0.997	0.241	99.9	0.997	0.988	0.961	0.711	0.354	0.961	0.588
100004-01	10.37	0.909	0.52	239.9	88.5	0.997	0.316	59.6	0.998	0.957	0.82	0.416	0.154	0.937	0.572
100004-02	8.47	0.901	0.52	251.7	87.2	0.996	0.311	53.9	0.997	0.959	0.831	0.394	0.112	0.95	0.62
100004-03	6.82	0.903	0.5	264.4	91.5	0.993	0.373	36.4	0.993	0.905	0.683	0.207	0.034	0.936	0.601
100004-04	12.01	0.899	0.52	269.4	101.5	0.996	0.244	80.7	0.998	0.985	0.939	0.619	0.258	0.958	0.588
100011-01	7.77	0.89	0.56	276.9	76.2	0.999	0.488	36.3	0.941	0.747	0.528	0.252	0.076	0.797	0.636
100011-02	6.83	0.893	0.56	270.2	71.9	0.999	0.472	33	0.936	0.706	0.484	0.225	0.064	0.793	0.637
100011-03	9.64	0.888	0.56	277.4	76.5	0.999	0.51	41.5	0.946	0.769	0.559	0.285	0.106	0.793	0.615
100011-04	12.67	0.889	0.55	280.3	78.3	0.999	0.488	55	0.953	0.844	0.676	0.369	0.172	0.813	0.593
100011-05	8.25	0.887	0.56	283.6	75.2	0.999	0.482	40.4	0.941	0.762	0.552	0.279	0.101	0.786	0.658
100011-07	6.86	0.874	0.55	249.7	69.5	0.998	0.175	51.3	0.949	0.831	0.655	0.345	0.149	0.814	0.627
100011-09	6.39	0.86	0.56	254.1	70.4	0.998	0.203	47.4	0.944	0.811	0.622	0.322	0.131	0.811	0.645
100031-01	14.72	0.903	0.51	262.5	91.6	0.998	0.168	97.2	0.998	0.983	0.948	0.719	0.356	0.948	0.554
100031-04	14.18	0.9	0.53	262.5	82.9	0.998	0.225	98	0.995	0.959	0.912	0.677	0.35	0.912	0.631
100031-05	15.2	0.901	0.51	261.6	88.2	0.998	0.172	103.4	0.996	0.983	0.956	0.763	0.391	0.952	0.572
100031-06	21.31	0.899	0.49	277.6	49.1	0.998	0.066	163.9	0.965	0.885	0.87	0.817	0.673	0.85	0.719
100032-01	17.23	0.906	0.52	272	96.2	0.998	0.194	126.5	0.997	0.99	0.98	0.86	0.511	0.968	0.594
100032-02	21.71	0.907	0.51	269	100.2	0.997	0.18	156.3	0.999	0.995	0.988	0.921	0.639	0.972	0.566
100032-04	16.65	0.896	0.53	263.1	85.3	0.998	0.198	117.7	0.996	0.987	0.966	0.772	0.427	0.952	0.59
100032-06	13.23	0.891	0.53	258	77.5	0.998	0.205	94.8	0.998	0.982	0.941	0.679	0.338	0.947	0.607
100032-11	27.29	0.911	0.5	248.7	91	0.997	0.17	190.5	0.997	0.994	0.989	0.959	0.787	0.974	0.529
100034-02	5.56	0.953	0.53	235.8	65.2	0.999	0.195	54.1	0.994	0.928	0.791	0.405	0.122	0.917	0.758
100041-01	18.73	0.91	0.52	245.3	92.4	0.998	0.193	129	0.999	0.993	0.97	0.776	0.466	0.944	0.541
100041-02	20.75	0.911	0.51	257.9	88.4	0.998	0.196	152.8	0.997	0.992	0.985	0.917	0.625	0.971	0.593
100041-03	19.16	0.909	0.52	247.9	84.7	0.998	0.186	143.2	0.999	0.994	0.985	0.895	0.583	0.97	0.596
100041-04	16.94	0.901	0.53	272	82.8	0.998	0.207	120.8	0.999	0.991	0.974	0.807	0.461	0.958	0.599
100041-05	17.73	0.913	0.52	255.4	94.7	0.998	0.185	134.7	0.997	0.992	0.983	0.857	0.509	0.969	0.588
100041-06	15.89	0.907	0.53	257.7	96.6	0.998	0.2	119.6	0.996	0.989	0.959	0.72	0.425	0.936	0.596
100041-07	17.2	0.916	0.51	249.5	92	0.998	0.192	129.4	0.996	0.992	0.983	0.871	0.517	0.973	0.585
100041-08	14.66	0.913	0.51	256.5	85.4	0.998	0.201	108	0.999	0.993	0.977	0.812	0.411	0.972	0.584
100042-01	14.46	0.895	0.52	269.5	91.2	0.998	0.236	89.7	0.996	0.982	0.937	0.636	0.308	0.949	0.543
100042-02	14.57	0.902	0.52	255.5	83.3	0.998	0.246	91.7	0.996	0.982	0.938	0.655	0.323	0.944	0.558
100042-03	13.1	0.903	0.53	276	99.5	0.998	0.227	89.6	0.998	0.985	0.942	0.654	0.314	0.954	0.584
100042-04	10.87	0.87	0.56	266.2	76.3	0.999	0.24	74.8	0.953	0.858	0.731	0.452	0.254	0.796	0.633
100042-06	27.98	0.916	0.51	257.7	89.4	0.998	0.227	190.6	0.997	0.993	0.988	0.949	0.736	0.97	0.57
100046-04	11.47	0.893	0.55	250.6	68.2	0.999	0.253	80.4	0.996	0.938	0.816	0.499	0.273	0.855	0.628
100046-05	13.6	0.891	0.55	255.2	71	0.999	0.285	90.1	0.996	0.947	0.839	0.538	0.309	0.851	0.621
100046-06	14.64	0.894	0.55	253.1	69.6	0.999	0.27	96.9	0.997	0.96	0.873	0.58	0.334	0.873	0.607
100046-12	12.75	0.89	0.56	248.6	67.1	0.999	0.262	91.3	0.995	0.945	0.839	0.539	0.311	0.851	0.649
100054-01	6.73	0.881	0.55	253.8	65.9	0.999	0.2	51.3	0.943	0.832	0.675	0.361	0.147	0.817	0.659
100054-02	5.76	0.875	0.55	251.2	63.8	0.999	0.187	44.1	0.94	0.808	0.624	0.308	0.11	0.825	0.655
100054-03	8.27	0.868	0.56	255.1	68.3	0.998	0.236	58.9	0.948	0.844	0.695	0.392	0.186	0.815	0.647
100054-04	6.17	0.873	0.55	265.9	73	0.999	0.206	45.1	0.944	0.828	0.653	0.321	0.109	0.828	0.637
100054-05	8.3	0.874	0.55	254.6	67.2	0.999	0.208	60.5	0.95	0.871	0.747	0.422	0.19	0.839	0.638
100058-01	15.84	0.91	0.52	285.3	122.5	0.997	0.272	96.2	0.999	0.991	0.962	0.695	0.335	0.962	0.535
100058-02	18.44	0.915	0.51	276.1	110	0.997	0.267	109.1	0.997	0.99	0.975	0.779	0.401	0.97	0.516
100058-03	17.59	0.919	0.52	246.7	91	0.998	0.28	105.2	0.997	0.989	0.967	0.741	0.377	0.96	0.529
100059-01	15.78	0.93	0.52	238.1	91.2	0.998	0.29	91	0.995	0.959	0.9	0.685	0.343	0.906	0.589
100059-02	15.04	0.927	0.52	251.9	96.4	0.998	0.278	88.3	0.995	0.959	0.898	0.667	0.325	0.911	0.587
100059-03	19.11	0.934	0.5	232.3	88.3	0.999	0.304	96.3	0.996	0.97	0.919	0.717	0.375	0.919	0.529
100059-04	16.31	0.929	0.51	242.5	95.5	0.998	0.291	88.3	0.995	0.955	0.894	0.684	0.334	0.907	0.564
100060-01	16.47	0.916	0.51	272.3	106.4	0.997	0.231	108.1	0.997	0.99	0.972	0.773	0.399	0.966	0.541
100060-02	16.63	0.925	0.52	251.6	93.1	0.998	0.231	112.4	0.999	0.992	0.974	0.787	0.417	0.965	0.557
100060-03	15.33	0.92	0.53	229.1	80.5	0.999	0.234	95	0.998	0.987	0.939	0.633	0.322	0.939	0.519
100062-01	18.61	0.933	0.52	241.8	87.5	0.998	0.348	97.7	0.996	0.969	0.916	0.715	0.377	0.916	0.553
100062-02	18.39	0.929	0.52	252.7	95.3	0.998	0.321	99.3	0.998	0.974	0.926	0.73	0.389	0.926	0.548
100062-03	18.35	0.932	0.51	252.1	95.8	0.998	0.326	92.1	0.996	0.97	0.918	0.706	0.353	0.924	0.52
100063-01	9.68	0.907	0.56	235.2	91.4	0.998	0.505	37.8	0.985	0.746	0.52	0.245	0.082	0.806	0.521
100063-02	9.25	0.914	0.54	241.4	77.2	0.999	0.55	33.3	0.987	0.76	0.514	0.208	0.054	0.852	0.525
100063-03	7.97	0.907	0.54	239.1	86.6	0.998	0.504	30.7	0.987	0.729	0.482	0.186	0.044	0.828	0.508
100063-04	5.64	0.917	0.54	237.5	73.3	0.999	0.532	21.8	0.978	0.639	0.374	0.098	0.015	0.844	0.539
100067-01	16.8	0.922	0.51	260.2	87.7	0.998	0.302	102.7	0.999	0.992	0.975	0.798	0.387	0.972	0.52
100067-02	12.85	0.916	0.51	266.9	97.1	0.998	0.226	92.6	0.997	0.988	0.969	0.752	0.329	0.971	0.562
100067-03	21.21	0.926	0.54	271.2	96.1	0.997	0.552	90.1	0.999	0.989	0.958	0.674	0.303	0.963	0.499
100068-01	15.18	0.921	0.51	256.1	97.1	0.998	0.285	91.3	0.999	0.99	0.968	0.737	0.318	0.971	0.503
100068-02	15.3	0.915	0.51	274.2	107	0.998	0.27	93.2	0.997	0.988	0.967	0.745	0.333	0.97	0.505
100068-03	19.34	0.927	0.54	254.8	87.8	0.998	0.527	86.7	0.999	0.988	0.95	0.641	0.285	0.961	0.509
100069-01	12.87	0.944	0.49	256.3	107.9	0.999	0.223	119.2	0.996	0.989	0.981	0.902	0.545	0.976	0.756
100069-02	15.88	0.942	0.48	284.3	120.3	0.999	0.238	138.7	0.996	0.991	0.984	0.933	0.652	0.976	0.735
100071-01	21.6	0.927	0.5	253.6	98.5	0.998	0.227	116.4	0.999	0.994	0.982	0.836	0.449	0.974	0.434
100071-02	17.95	0.929	0.51	267.4	100.9	0.998	0.255	107.8	0.997	0.99	0.977	0.803	0.406	0.972	0.489
100071-03	18.53	0.92	0.51	264.7	102.1	0.998	0.242	107.6	0.997	0.99	0.973	0.793	0.406	0.968	0.471
100072-01	16.24	0.92	0.51	279	108.1	0.997	0.244	94.9	0.999	0.991	0.97	0.733	0.336	0.973	0.473
100072-02	17.47	0.922	0.52	270.6	105.3	0.997	0.239	106.7	0.997	0.99	0.976	0.795	0.398	0.971	0.49
100072-03	21.61	0.927	0.51	259.4	101	0.998	0.246	120.3	0.999	0.994	0.982	0.838	0.462	0.971	0.453
100073-01	17.13	0.929	0.52	261.8	97.1	0.998	0.245	104.3	0.999	0.992	0.976	0.777	0.382	0.973	0.49
100073-02	19.11	0.932	0.53	270	93.9	0.998	0.273	112.7	0.999	0.992	0.974	0.785	0.416	0.964	0.486
100073-03	15.75	0.927	0.52	270.4	106.5	0.997	0.264	90.7	0.997	0.988	0.964	0.7	0.311	0.968	0.475
100073-04	21.44	0.928	0.51	263	101.8	0.997	0.243	127.7	0.999	0.993	0.981	0.852	0.506	0.967	0.485
100074-01	16.3	0.925	0.52	271.5	91.1	0.998	0.234	106.4	0.999	0.992	0.974	0.79	0.4	0.968	0.524
100074-02	19.4	0.928	0.5	235.3	85.9	0.998	0.227	135.9	0.999	0.994	0.986	0.897	0.564	0.973	0.567
100074-03	19.23	0.93	0.52	251.7	92.4	0.998	0.25	125.4	0.999	0.994	0.984	0.862	0.493	0.972	0.53
100074-04	21.2	0.931	0.51	247.8	91.2	0.998	0.265	127.5	0.997	0.992	0.982	0.867	0.502	0.972	0.494
100074-05	18.08	0.929	0.52	271	90.3	0.998	0.287	109.8	0.999	0.993	0.978	0.792	0.405	0.972	0.507
100074-06	17.68	0.932	0.53	259.8	94.8	0.998	0.269	108.1	0.997	0.991	0.975	0.781	0.396	0.97	0.501
100075-01	17.32	0.918	0.53	268.7	97.9	0.998	0.26	115.2	0.999	0.993	0.98	0.818	0.434	0.971	0.545
100075-02	25.34	0.922	0.49	257.9	102.8	0.998	0.221	131.1	0.997	0.991	0.981	0.88	0.545	0.969	0.419
100075-03	17.46	0.924	0.53	277.2	100.4	0.998	0.277	112.5	0.999	0.993	0.977	0.803	0.426	0.969	0.534
100075-04	21.05	0.928	0.52	251.8	93	0.998	0.252	121.7	0.999	0.992	0.98	0.84	0.471	0.969	0.476
100076-01	17.78	0.923	0.53	280.4	98.5	0.997	0.281	114.4	0.999	0.993	0.979	0.807	0.428	0.972	0.54
100076-02	19.16	0.918	0.52	285.5	107.6	0.997	0.266	121.2	0.997	0.991	0.982	0.852	0.474	0.973	0.521
100076-03	19.56	0.924	0.52	267.4	97.5	0.998	0.268	118.8	0.999	0.994	0.981	0.831	0.458	0.972	0.503
100078-01	19.14	0.918	0.5	239.5	88.3	0.998	0.284	107.9	0.997	0.991	0.978	0.814	0.411	0.974	0.499
100078-02	17.82	0.905	0.5	271.4	107.2	0.997	0.273	96.9	0.997	0.99	0.974	0.764	0.35	0.974	0.475
100078-03	20.04	0.91	0.49	257.9	98.3	0.998	0.261	96	0.997	0.991	0.975	0.769	0.344	0.975	0.411
100078-04	21.76	0.91	0.49	257.5	99.5	0.998	0.288	105.1	0.999	0.994	0.98	0.805	0.396	0.976	0.432
100080-01	12.12	0.902	0.51	242.6	91.1	0.998	0.186	89.8	0.999	0.991	0.965	0.711	0.306	0.972	0.576
100080-02	11.16	0.889	0.51	240.9	91.5	0.998	0.133	84.9	0.996	0.987	0.96	0.679	0.28	0.971	0.556
100080-03	11.21	0.905	0.51	227.1	82.3	0.999	0.169	86.8	0.998	0.99	0.959	0.683	0.289	0.968	0.59
100080-04	12.08	0.899	0.51	245.2	92.8	0.998	0.159	91.1	0.999	0.991	0.965	0.707	0.314	0.969	0.569
100087-01	12.09	0.882	0.51	283.8	99	0.997	0.163	88.1	0.999	0.991	0.965	0.699	0.298	0.972	0.557
100087-02	11.87	0.891	0.51	272.9	104.9	0.998	0.178	87	0.996	0.988	0.966	0.705	0.291	0.972	0.563
100087-03	11.83	0.895	0.51	280.2	97.3	0.998	0.173	87.7	0.996	0.988	0.966	0.716	0.294	0.973	0.57
100089-01	11.93	0.901	0.51	252	90.2	0.999	0.208	87.3	0.998	0.989	0.955	0.679	0.296	0.965	0.582
100089-02	12.45	0.898	0.52	267.7	88.7	0.998	0.215	87.2	0.996	0.985	0.946	0.661	0.299	0.957	0.569
100089-03	12.33	0.898	0.51	285.4	98.1	0.998	0.213	86.5	0.998	0.99	0.963	0.686	0.29	0.971	0.565
100089-04	12.24	0.893	0.51	269.5	101.8	0.998	0.199	88.1	0.998	0.99	0.958	0.681	0.301	0.967	0.568
100091-01	17.08	0.919	0.5	267.5	92	0.998	0.236	97.4	0.999	0.991	0.965	0.745	0.352	0.965	0.472
100091-02	17.83	0.918	0.48	254.9	101.1	0.998	0.241	97	0.999	0.993	0.976	0.791	0.348	0.976	0.451
100091-03	17.33	0.926	0.48	247.9	95.6	0.999	0.247	95.3	0.997	0.99	0.973	0.778	0.337	0.973	0.458
100091-04	17.51	0.922	0.49	259.6	101.3	0.998	0.246	97.5	0.999	0.993	0.976	0.782	0.349	0.976	0.464
100091-05	17	0.924	0.49	246.8	96.7	0.999	0.242	93.6	0.997	0.99	0.97	0.753	0.327	0.973	0.456
100091-06	17.66	0.92	0.48	253.1	99.1	0.998	0.237	96.2	0.999	0.992	0.976	0.792	0.347	0.976	0.449
100091-07	17.03	0.906	0.49	310.4	116.5	0.997	0.243	93.4	0.997	0.99	0.973	0.757	0.328	0.976	0.459
100092-01	14.11	0.89	0.5	306.2	108.8	0.997	0.2	94.6	0.998	0.991	0.974	0.768	0.338	0.977	0.535
100092-02	14.01	0.896	0.51	271.8	101.3	0.998	0.194	97.5	0.997	0.99	0.971	0.751	0.352	0.971	0.546
100092-04	12.86	0.903	0.51	266.4	98	0.998	0.191	95.8	0.999	0.991	0.966	0.729	0.342	0.966	0.579
100092-05	12.97	0.894	0.51	262.4	95	0.998	0.193	93.6	0.999	0.991	0.97	0.74	0.33	0.973	0.563
100092-06	12.15	0.886	0.51	259.5	95.1	0.998	0.169	91.4	0.999	0.99	0.959	0.692	0.32	0.963	0.572
100093-01	11.53	0.892	0.51	272	101	0.998	0.199	83.5	0.998	0.99	0.964	0.69	0.271	0.974	0.571
100093-02	10.84	0.917	0.55	206.9	70.1	0.999	0.202	83.7	0.996	0.965	0.868	0.538	0.283	0.902	0.626
100093-03	10.6	0.891	0.51	270.6	102.1	0.997	0.195	75.1	0.998	0.988	0.952	0.621	0.224	0.971	0.557
100094-01	12.74	0.88	0.5	303.8	119.2	0.996	0.139	94.3	0.998	0.991	0.974	0.767	0.337	0.977	0.543
100094-02	12.55	0.899	0.51	256.2	94.1	0.998	0.171	96.2	0.996	0.989	0.969	0.743	0.344	0.969	0.581
100094-03	13.37	0.894	0.5	263.6	102.3	0.998	0.156	98	0.996	0.99	0.974	0.774	0.357	0.974	0.547
100094-04	13.12	0.891	0.5	274.9	106.1	0.997	0.152	99.9	0.996	0.989	0.975	0.792	0.368	0.975	0.565
100094-05	14.31	0.892	0.5	275.9	95.3	0.997	0.156	97.9	0.999	0.992	0.975	0.776	0.356	0.975	0.516
100095-01	10.96	0.897	0.51	245.9	92.8	0.998	0.153	83.8	0.998	0.988	0.953	0.666	0.276	0.967	0.567
100095-02	11.6	0.888	0.5	260.7	99	0.998	0.153	84.2	0.996	0.988	0.962	0.697	0.275	0.973	0.536
100095-03	11.03	0.898	0.5	249.4	94.5	0.998	0.154	84.1	0.996	0.988	0.962	0.694	0.274	0.973	0.566
100095-04	11.52	0.889	0.5	290.5	101.6	0.998	0.163	84.7	0.998	0.99	0.966	0.713	0.276	0.976	0.556
100098-01	15.24	0.894	0.5	268.3	93.5	0.997	0.363	89	0.999	0.992	0.972	0.735	0.302	0.978	0.482
100098-02	12.91	0.892	0.5	252.7	87.6	0.997	0.292	80.3	0.996	0.988	0.962	0.68	0.25	0.972	0.471
100098-03	13.87	0.896	0.5	245.9	83.9	0.997	0.336	87.1	0.999	0.991	0.967	0.713	0.291	0.974	0.499
100107-01	11.57	0.902	0.5	254.2	95.6	0.998	0.16	86.8	0.996	0.988	0.966	0.725	0.289	0.972	0.561
100107-02	10.93	0.901	0.5	256.1	95.6	0.998	0.143	85.8	0.999	0.99	0.966	0.716	0.283	0.973	0.577
100107-03	11.1	0.899	0.5	256.6	93.7	0.998	0.151	87.1	0.996	0.988	0.965	0.726	0.291	0.972	0.58
100107-04	11.18	0.898	0.5	260.1	95.7	0.998	0.154	86.1	0.998	0.99	0.966	0.719	0.285	0.972	0.572
100107-05	11.47	0.897	0.5	250.5	94.4	0.998	0.128	86.4	0.999	0.99	0.965	0.713	0.287	0.972	0.544
100109-01	14.12	0.892	0.5	257.1	88.4	0.997	0.327	85.7	0.999	0.991	0.968	0.717	0.282	0.975	0.477
100109-02	15.68	0.899	0.5	247	86.3	0.997	0.359	87.1	0.996	0.989	0.968	0.728	0.289	0.974	0.459

Supplementary Section S9. Continued

100109-03	13.21	0.89	0.5	247.9	85.2	0.996	0.311	81.1	0.997	0.988	0.962	0.679	0.255	0.973	0.472
100109-04	13.45	0.895	0.5	250.8	87.1	0.997	0.305	80.2	0.999	0.99	0.962	0.675	0.248	0.973	0.462
100111-01	12.69	0.894	0.5	252.4	86.8	0.997	0.337	80.1	0.999	0.99	0.961	0.673	0.248	0.973	0.726
100111-02	7.54	0.899	0.48	245	97.9	0.998	0.157	47.5	0.995	0.968	0.864	0.349	0.068	0.968	0.652
100111-03	14.21	0.893	0.5	260.9	88.6	0.996	0.362	84.7	0.996	0.988	0.965	0.704	0.277	0.975	0.713
100111-04	16.16	0.897	0.49	239.6	85	0.997	0.332	87.4	0.997	0.989	0.969	0.733	0.291	0.975	0.628
100111-05	12.93	0.891	0.5	263.3	89.7	0.996	0.32	82.4	0.996	0.988	0.963	0.691	0.263	0.973	0.719
100112-01	11.75	0.89	0.5	273.9	106.2	0.997	0.155	86.7	0.998	0.99	0.967	0.717	0.29	0.973	0.551
100112-02	11.67	0.896	0.5	243.4	92.3	0.998	0.167	86.3	0.996	0.988	0.965	0.716	0.286	0.971	0.56
100112-03	12.82	0.897	0.49	240.6	93.6	0.998	0.146	84.5	0.999	0.99	0.964	0.707	0.273	0.975	0.488
100113-01	10.8	0.898	0.5	250	89.7	0.999	0.153	83.5	0.998	0.988	0.958	0.693	0.271	0.97	0.573
100113-02	12.08	0.894	0.5	249.9	90.6	0.998	0.157	88.6	0.996	0.987	0.966	0.743	0.301	0.972	0.545
100113-03	12.47	0.892	0.49	244.6	91.3	0.998	0.157	84.4	0.998	0.989	0.963	0.712	0.275	0.973	0.505
100113-04	11.65	0.892	0.5	254	93	0.998	0.152	85.1	0.998	0.989	0.962	0.712	0.279	0.969	0.54
100114-01	17.98	0.905	0.47	250.5	100.2	0.998	0.152	92.7	0.999	0.992	0.973	0.772	0.329	0.976	0.384
100114-02	14.19	0.914	0.49	249.1	94.6	0.999	0.16	91.9	0.996	0.988	0.969	0.765	0.323	0.972	0.483
100114-03	13.77	0.915	0.49	239.7	88.9	0.999	0.156	90.9	0.996	0.988	0.968	0.762	0.315	0.971	0.49
100114-05	18.57	0.914	0.47	240.7	97	0.998	0.153	92.7	0.999	0.991	0.973	0.776	0.327	0.976	0.371
100115-01	16.94	0.897	0.47	264.2	104.6	0.998	0.169	89.1	0.999	0.991	0.972	0.752	0.303	0.978	0.4
100115-02	14.84	0.9	0.48	256.1	98.8	0.998	0.176	89.9	0.996	0.988	0.97	0.759	0.309	0.975	0.463
100115-03	15.45	0.891	0.48	265.1	103	0.998	0.197	89.7	0.999	0.991	0.972	0.755	0.308	0.977	0.458
100115-04	13.6	0.904	0.49	249.3	94	0.999	0.173	88.2	0.996	0.988	0.966	0.741	0.299	0.972	0.494
100115-05	15.15	0.903	0.49	252.8	96.5	0.998	0.186	91.7	0.999	0.991	0.971	0.759	0.321	0.974	0.47
100115-07	15.39	0.897	0.48	255.7	97	0.998	0.182	90.2	0.999	0.991	0.971	0.752	0.31	0.974	0.452
100115-08	14.84	0.903	0.48	247.8	94.7	0.998	0.175	89.8	0.996	0.989	0.97	0.756	0.307	0.975	0.462
100116-02	14.58	0.889	0.48	272	108.8	0.997	0.127	81.9	0.998	0.985	0.945	0.705	0.285	0.961	0.44
100116-03	15.96	0.893	0.48	252.5	99	0.997	0.132	87	0.996	0.984	0.947	0.729	0.326	0.956	0.431
100116-04	13.95	0.893	0.48	249.2	95.4	0.996	0.132	86.5	0.998	0.984	0.943	0.723	0.326	0.954	0.491
100118-01	10.94	0.902	0.49	247.7	92.2	0.999	0.118	84.9	0.998	0.989	0.963	0.724	0.277	0.973	0.553
100118-02	13.6	0.88	0.48	289.4	119.3	0.997	0.115	84.8	0.996	0.988	0.966	0.726	0.281	0.974	0.445
100118-03	12.37	0.893	0.49	255.8	98.6	0.998	0.106	84.7	0.996	0.987	0.965	0.73	0.277	0.974	0.482
100118-04	11.86	0.893	0.49	261.6	98.3	0.998	0.133	88.4	0.998	0.99	0.968	0.75	0.301	0.974	0.54
100118-05	11.58	0.873	0.49	301.2	119.1	0.996	0.104	80.9	0.996	0.987	0.964	0.7	0.255	0.973	0.492
100118-06	12.48	0.897	0.49	253	95.5	0.998	0.126	88.4	0.999	0.99	0.968	0.745	0.3	0.974	0.509
100118-07	11.24	0.898	0.49	254.8	96.8	0.998	0.104	85	0.996	0.987	0.964	0.728	0.279	0.97	0.529
100119-01	18.22	0.891	0.5	340.9	127.2	0.994	0.319	100.8	0.999	0.993	0.978	0.783	0.378	0.975	0.437
100119-09	18.85	0.917	0.49	257	100.8	0.998	0.254	103.2	0.999	0.993	0.979	0.811	0.386	0.976	0.461
100119-15	19.98	0.891	0.49	337.6	126.3	0.995	0.329	102.1	0.999	0.993	0.981	0.812	0.39	0.979	0.41
100119-16	18.82	0.909	0.5	284.7	102.9	0.997	0.318	110.8	0.997	0.991	0.98	0.844	0.44	0.975	0.462
100119-17	17.12	0.896	0.5	280.9	103.6	0.996	0.269	107.5	0.999	0.993	0.977	0.814	0.421	0.972	0.471
100122-01	17.25	0.906	0.5	280.3	105.5	0.997	0.246	113.2	0.999	0.993	0.98	0.841	0.455	0.974	0.479
100122-02	18.89	0.896	0.49	311.2	120.7	0.996	0.256	112.2	0.999	0.994	0.983	0.848	0.452	0.978	0.437
100122-03	18.96	0.89	0.5	315.4	120	0.995	0.263	113.4	0.997	0.992	0.982	0.846	0.453	0.977	0.443
100122-04	17.82	0.899	0.49	307.1	114.1	0.996	0.274	108.1	0.999	0.993	0.98	0.83	0.428	0.976	0.453
100122-05	17.86	0.908	0.5	281.6	104.5	0.997	0.269	115.6	0.997	0.991	0.979	0.849	0.469	0.971	0.479
100122-06	15.77	0.923	0.48	251.4	100	0.999	0.236	84.9	0.999	0.99	0.967	0.732	0.271	0.976	0.443
100122-07	18.23	0.921	0.48	257.6	102.3	0.999	0.245	98.6	0.999	0.992	0.977	0.809	0.364	0.977	0.45
100123-02	16.09	0.944	0.48	265.1	107.7	0.999	0.24	142.7	0.996	0.991	0.984	0.935	0.671	0.975	0.744
100125-01	18.54	0.911	0.5	289.7	102	0.997	0.348	111.8	0.997	0.991	0.98	0.852	0.45	0.975	0.491
100125-02	20.09	0.91	0.5	289.5	104.1	0.997	0.357	109.6	0.999	0.993	0.98	0.83	0.434	0.975	0.46
100125-03	18.1	0.912	0.51	308.7	108.1	0.997	0.373	103.1	0.999	0.992	0.974	0.794	0.394	0.972	0.482
100125-04	17.77	0.908	0.5	307.5	108.8	0.997	0.354	103.5	0.999	0.992	0.977	0.809	0.399	0.975	0.481
100125-08	19.74	0.907	0.49	313.4	116.1	0.997	0.329	102.5	0.997	0.991	0.978	0.82	0.393	0.977	0.425
100126-02	16.97	0.921	0.51	241.7	85.7	0.999	0.255	118.8	0.996	0.99	0.977	0.854	0.484	0.969	0.59
100126-03	14.59	0.912	0.5	266.8	99.6	0.999	0.222	99.7	0.999	0.991	0.975	0.8	0.373	0.975	0.551
100126-04	16.59	0.922	0.51	230.8	79.4	0.999	0.245	119.4	0.996	0.99	0.976	0.846	0.481	0.967	0.599
100126-05	17.15	0.921	0.51	240	86.5	0.999	0.236	119.5	0.999	0.993	0.979	0.852	0.485	0.971	0.574
100127-01	20.56	0.913	0.5	288	103.2	0.997	0.369	109.2	0.997	0.991	0.978	0.837	0.433	0.975	0.453
100127-02	17.86	0.908	0.5	292.5	103	0.997	0.352	105.5	0.997	0.99	0.977	0.818	0.406	0.973	0.486
100127-06	19.23	0.908	0.5	305.3	109.4	0.997	0.362	106.3	0.999	0.993	0.979	0.818	0.414	0.974	0.464
100127-07	19.06	0.91	0.51	302.7	106.8	0.997	0.376	105.8	0.997	0.99	0.976	0.813	0.411	0.971	0.473
100127-12	26.35	0.905	0.49	287.3	108.6	0.996	0.213	162.6	0.997	0.994	0.989	0.949	0.687	0.979	0.458
100127-16	19.33	0.9	0.5	337.4	122.7	0.995	0.364	96.1	0.999	0.992	0.974	0.77	0.355	0.974	0.42
100128-01	14.2	0.904	0.49	293.7	103.7	0.996	0.209	87.2	0.997	0.99	0.974	0.734	0.294	0.978	0.423
100128-02	17.98	0.911	0.49	303.3	101.2	0.996	0.186	105.6	0.999	0.995	0.984	0.829	0.407	0.981	0.395
100128-03	13.06	0.904	0.49	287.2	100.9	0.996	0.206	82.8	0.999	0.992	0.97	0.699	0.265	0.979	0.439
100129-07	16.04	0.919	0.5	236	82.7	0.999	0.223	110.5	0.996	0.989	0.974	0.831	0.438	0.967	0.556
100129-09	14.3	0.914	0.5	240.8	85.5	0.998	0.202	99.5	0.999	0.991	0.974	0.798	0.371	0.974	0.547
100129-12	13.79	0.92	0.5	239.1	85.9	0.999	0.203	97.5	0.996	0.988	0.967	0.778	0.361	0.967	0.556
100129-15	13.54	0.916	0.5	243.5	85.7	0.999	0.213	100	0.998	0.99	0.97	0.789	0.374	0.967	0.589
100129-16	17.34	0.92	0.49	232.6	88.1	0.999	0.204	99.9	0.996	0.987	0.965	0.779	0.378	0.965	0.456
100130-01	17.29	0.897	0.49	314.5	116	0.996	0.337	81.9	0.999	0.991	0.967	0.69	0.262	0.977	0.389
100130-02	13.67	0.903	0.5	305.5	108.3	0.997	0.319	77.5	0.996	0.986	0.954	0.648	0.238	0.971	0.446
100130-03	14.1	0.896	0.49	292.4	106.2	0.997	0.314	78.1	0.996	0.987	0.959	0.66	0.239	0.974	0.428
100130-04	12.48	0.881	0.5	300	107.2	0.997	0.289	72.9	0.999	0.985	0.94	0.605	0.214	0.968	0.441
100130-05	14.08	0.893	0.5	312.5	111	0.996	0.331	78.5	0.999	0.988	0.951	0.648	0.247	0.97	0.446
100130-06	16.21	0.909	0.5	281	99.5	0.997	0.356	85.4	0.999	0.99	0.963	0.7	0.286	0.971	0.43
100130-07	14.58	0.905	0.5	271.7	97.9	0.997	0.319	86.2	0.996	0.988	0.964	0.708	0.29	0.971	0.465
100131-01	16.43	0.918	0.51	239.1	85.3	0.999	0.246	116.8	0.996	0.99	0.975	0.843	0.472	0.966	0.594
100131-02	20.41	0.917	0.5	243.3	90.3	0.999	0.238	123.5	0.999	0.992	0.978	0.85	0.503	0.966	0.501
100131-03	15.87	0.918	0.5	249.7	88.6	0.999	0.237	113.4	0.999	0.993	0.979	0.846	0.454	0.973	0.587
100131-04	15.9	0.913	0.51	262.4	95.9	0.998	0.236	111.5	0.996	0.989	0.975	0.832	0.445	0.968	0.577
100132-01	15.88	0.917	0.5	241.2	86.9	0.999	0.221	103.5	0.999	0.991	0.97	0.776	0.385	0.966	0.528
100132-02	16.16	0.918	0.5	241.6	85.5	0.999	0.231	106.6	0.999	0.992	0.976	0.806	0.402	0.971	0.54
100132-03	12.77	0.91	0.5	232.5	81.3	0.998	0.21	86.3	0.996	0.985	0.954	0.693	0.289	0.963	0.538
100133-01	17.07	0.916	0.5	256.9	90.7	0.998	0.241	109.3	0.999	0.993	0.98	0.807	0.409	0.975	0.532
100133-02	15.73	0.914	0.5	263.8	92.3	0.998	0.247	104.3	0.996	0.99	0.977	0.801	0.388	0.975	0.554
100133-03	13	0.92	0.51	256.3	90	0.998	0.219	91.8	0.998	0.991	0.97	0.732	0.318	0.973	0.569
100133-07	16.01	0.921	0.52	250.7	88.1	0.998	0.245	112.4	0.999	0.993	0.979	0.796	0.415	0.971	0.584
100134-01	15.6	0.921	0.51	234.2	83.8	0.999	0.224	106.3	0.999	0.989	0.964	0.776	0.403	0.957	0.554
100134-02	14.6	0.917	0.5	235	83.7	0.999	0.216	99.1	0.996	0.987	0.966	0.768	0.364	0.966	0.545
100134-03	16.02	0.911	0.5	244.5	88	0.998	0.225	104.2	0.997	0.988	0.97	0.793	0.395	0.967	0.529
100134-05	15.19	0.919	0.5	237.8	86	0.999	0.219	98	0.999	0.991	0.971	0.771	0.356	0.971	0.52
100135-01	19.36	0.916	0.49	243.7	88.9	0.998	0.238	117.4	0.996	0.991	0.98	0.866	0.479	0.973	0.499
100135-02	14.35	0.904	0.51	292.2	113.5	0.997	0.228	99.5	0.996	0.987	0.963	0.767	0.376	0.963	0.568
100136-02	15.54	0.916	0.5	247.4	89.6	0.999	0.219	104.5	0.999	0.991	0.973	0.791	0.395	0.97	0.543
100136-03	15.22	0.919	0.5	248.9	89.1	0.998	0.219	102.6	0.996	0.988	0.969	0.785	0.384	0.966	0.542
100136-04	15.38	0.909	0.5	260	94.5	0.998	0.22	100.6	0.998	0.99	0.969	0.77	0.373	0.966	0.529
100137-01	8.5	0.889	0.5	242.3	84.6	0.998	0.131	69.3	0.995	0.979	0.931	0.593	0.185	0.964	0.587
100137-02	8.73	0.897	0.5	229.8	79.5	0.999	0.134	71.9	0.998	0.981	0.933	0.612	0.201	0.962	0.597
100137-03	8.87	0.887	0.51	240	83.5	0.998	0.127	73.6	0.996	0.977	0.928	0.616	0.217	0.957	0.598
100137-04	9.18	0.879	0.5	258.4	94.7	0.997	0.128	69.4	0.998	0.981	0.932	0.595	0.187	0.966	0.545
100137-05	8.98	0.893	0.5	245.4	86.1	0.998	0.143	71.5	0.995	0.981	0.942	0.62	0.196	0.966	0.573
100137-06	8.58	0.887	0.51	239.5	83	0.998	0.128	70.8	0.998	0.98	0.927	0.596	0.198	0.959	0.591
100137-07	8.35	0.891	0.51	232.7	83	0.998	0.119	64.6	0.998	0.969	0.894	0.535	0.167	0.952	0.55
100137-10	9.48	0.888	0.5	241	86.6	0.998	0.134	72.2	0.996	0.98	0.935	0.617	0.203	0.961	0.552
100137-12	8.03	0.881	0.5	240.9	84.6	0.998	0.121	66.2	0.995	0.976	0.92	0.564	0.168	0.959	0.589
100140-01	6.64	0.884	0.51	258.3	91.3	0.997	0.123	54.9	0.997	0.967	0.873	0.438	0.111	0.961	0.594
100140-02	8.71	0.892	0.49	239.2	89.6	0.998	0.11	56	0.995	0.965	0.874	0.453	0.117	0.952	0.455
100140-03	7.54	0.886	0.51	248.9	87.4	0.997	0.129	62.2	0.995	0.972	0.902	0.513	0.151	0.957	0.596
100140-04	8.78	0.89	0.5	239.5	87.6	0.997	0.122	61.2	0.996	0.972	0.901	0.508	0.143	0.957	0.5
100140-06	6.52	0.892	0.51	235.3	79.2	0.998	0.113	55.9	0.998	0.97	0.88	0.444	0.117	0.958	0.606
100140-07	18.35	0.889	0.53	247.5	78.5	0.998	0.614	69.9	0.998	0.969	0.858	0.501	0.219	0.933	0.633
100140-08	7.55	0.887	0.5	244.3	85	0.997	0.124	58.3	0.998	0.977	0.908	0.479	0.123	0.968	0.553
100141-01	9.26	0.897	0.5	236.6	82.2	0.999	0.119	77.9	0.998	0.985	0.948	0.654	0.239	0.967	0.6
100141-02	7.39	0.876	0.5	254.1	89	0.998	0.103	60.4	0.995	0.976	0.914	0.506	0.134	0.963	0.573
100141-03	9.14	0.896	0.51	244.6	86.6	0.999	0.113	78.4	0.996	0.98	0.937	0.652	0.247	0.958	0.609
100141-04	8.38	0.887	0.51	243.4	86.3	0.998	0.107	71.7	0.996	0.979	0.933	0.611	0.202	0.961	0.602
100141-05	8.63	0.883	0.5	244.4	84.8	0.998	0.116	69.8	0.996	0.98	0.936	0.601	0.188	0.967	0.574
100141-06	9.05	0.889	0.5	251.1	91.2	0.998	0.107	71.3	0.998	0.982	0.933	0.605	0.2	0.962	0.555
100145-01	9.05	0.891	0.51	246.7	87.2	0.999	0.111	77.6	0.996	0.981	0.94	0.648	0.241	0.96	0.606
100145-02	9.16	0.89	0.49	252.8	93.3	0.998	0.098	68.3	0.998	0.983	0.936	0.581	0.179	0.97	0.518
100145-03	10.29	0.894	0.5	244.1	88.2	0.999	0.118	79.4	0.996	0.982	0.944	0.665	0.252	0.963	0.55
100145-04	8.17	0.882	0.51	259.4	93.1	0.998	0.105	69.3	0.995	0.979	0.928	0.585	0.188	0.964	0.595
100146-01	16.18	0.912	0.49	253.5	95.1	0.998	0.207	104.4	0.999	0.992	0.976	0.816	0.403	0.973	0.512
100146-02	12.63	0.904	0.51	261.3	96.8	0.998	0.199	89.9	0.996	0.986	0.96	0.73	0.316	0.967	0.56
100146-03	16.9	0.916	0.51	238.4	87.8	0.998	0.214	115.6	0.997	0.988	0.97	0.823	0.465	0.958	0.549
100146-04	15.84	0.915	0.51	234.6	84.3	0.998	0.219	115.3	0.998	0.99	0.968	0.813	0.461	0.955	0.59
100146-05	13.5	0.914	0.51	251.4	91.5	0.998	0.2	100.3	0.998	0.99	0.969	0.782	0.378	0.965	0.585
100147-01	15.89	0.942	0.48	259.2	105.3	0.999	0.225	146	0.996	0.991	0.985	0.94	0.688	0.976	0.753
100147-02	14.93	0.946	0.48	251.1	103.8	0.999	0.23	138.2	0.998	0.993	0.986	0.928	0.647	0.977	0.76
100148-01	12.07	0.909	0.5	276	94.9	0.998	0.179	84.8	0.999	0.99	0.965	0.711	0.28	0.975	0.546
100148-02	12.35	0.897	0.5	257.1	95.7	0.997	0.167	87.6	0.996	0.987	0.966	0.731	0.298	0.972	0.537
100148-03	12.37	0.917	0.5	237.1	87.6	0.999	0.175	93.4	0.996	0.988	0.968	0.758	0.332	0.971	0.578
100148-04	16.61	0.941	0.48	310.2	135.3	0.999	0.241	105.4	0.931	0.884	0.858	0.733	0.457	0.851	0.644
100148-05	11.71	0.894	0.5	289.4	116.1	0.997	0.162	83.2	0.996	0.987	0.962	0.696	0.273	0.972	0.538
100150-01	16.57	0.91	0.49	295.6	120.5	0.997	0.197	100.9	0.997	0.99	0.976	0.806	0.383	0.974	0.479
100150-02	17.45	0.913	0.49	278.3	111.8	0.998	0.195	104.1	0.999	0.993	0.98	0.819	0.4	0.978	0.469
100150-03	14.81	0.91	0.5	298	116.7	0.997	0.193	103.7	0.996	0.989	0.976	0.812	0.399	0.974	0.547
100150-04	16.21	0.923	0.5	247.2	90.4	0.999	0.196	119.4	0.999	0.994	0.982	0.864	0.484	0.975	0.577
100150-05	20.32	0.922	0.49	259.1	104.5	0.998	0.197	125.1	0.999	0.994	0.985	0.883	0.515	0.976	0.484
100150-06	18.46	0.923	0.5	258.3	101.5	0.998	0.196	123.3	0.997	0.991	0.982	0.875	0.504	0.974	0.524
100151-01	16.61	0.909	0.5	288.5	112.4	0.997	0.199	111.2	0.999	0.993	0.979	0.829	0.441	0.972	0.528
100151-02	16.04	0.92	0.5	264.9	100	0.998	0.204	114.5	0.996	0.991	0.98	0.855	0.461	0.975	0.563
100151-03	15.93	0.927	0.51	245.4	89.4	0.999	0.201	118.7	0.999	0.993	0.981	0.855	0.476	0.974	0.588
100151-04	16.29	0.919	0.51	266.6	99.3	0.998	0.193	119.9	0.996	0.991	0.98	0.858	0.485	0.973	0.574
100151-05	21.01	0.925	0.5	244.5	91.7	0.998	0.2	138.8	0.999	0.995	0.987	0.905	0.574	0.976	0.521
100151-06	16.12	0.917	0.51	279.2	106.3	0.998	0.2	116.2	0.999	0.993	0.981	0.842	0.463	0.972	0.569

Supplementary Section S9. Continued

100152-04	12.63	0.914	0.5	242.8	91.7	0.999	0.169	95	0.998	0.991	0.97	0.764	0.343	0.97	0.574
100152-05	13.6	0.907	0.49	267.2	109.5	0.998	0.158	84	0.996	0.987	0.964	0.709	0.277	0.973	0.464
100152-06	15.16	0.913	0.49	248.5	97	0.998	0.18	98.2	0.999	0.992	0.974	0.79	0.364	0.974	0.5
100152-09	12.49	0.91	0.5	244.8	91.1	0.998	0.169	93.4	0.996	0.988	0.968	0.76	0.333	0.971	0.569
100152-10	13.93	0.906	0.49	269.2	106.8	0.998	0.169	94.7	0.999	0.991	0.973	0.771	0.342	0.976	0.518
100152-11	14.22	0.905	0.5	269.2	105.6	0.998	0.173	99	0.996	0.989	0.974	0.79	0.369	0.974	0.533
100154-01	15.36	0.923	0.5	245.5	84.9	0.998	0.231	103.7	0.999	0.993	0.978	0.795	0.384	0.975	0.552
100154-02	16.61	0.919	0.51	258.8	91.5	0.998	0.254	110.4	0.996	0.991	0.979	0.826	0.422	0.973	0.56
100154-03	14.21	0.916	0.5	249.6	87.5	0.998	0.214	95.2	0.996	0.99	0.972	0.754	0.337	0.972	0.536
100154-05	15.31	0.921	0.51	248.7	86.9	0.999	0.241	105.3	0.996	0.99	0.977	0.806	0.393	0.972	0.57
100156-04	13.35	0.945	0.48	277.2	120.2	0.999	0.217	111	0.998	0.992	0.981	0.879	0.483	0.977	0.679
100156-05	14.59	0.942	0.48	268.9	112.6	0.999	0.222	130.9	0.998	0.993	0.985	0.92	0.608	0.977	0.735
100157-01	13.39	0.949	0.48	251.4	101.8	0.999	0.212	123.8	0.996	0.99	0.981	0.908	0.569	0.975	0.743
100157-03	13.43	0.946	0.49	229.3	90.4	0.999	0.214	131	0.996	0.99	0.982	0.919	0.613	0.974	0.776
100158-01	16.46	0.919	0.49	258.8	99.2	0.998	0.177	107.5	0.999	0.993	0.98	0.834	0.422	0.975	0.499
100158-02	17.08	0.92	0.49	255.6	100.9	0.998	0.178	104.8	0.999	0.993	0.979	0.828	0.408	0.977	0.471
100158-03	14.49	0.925	0.5	247.7	91.3	0.999	0.179	104.6	0.996	0.99	0.977	0.83	0.405	0.975	0.552
100159-02	14.09	0.921	0.5	236.7	88	0.999	0.173	99	0.999	0.992	0.974	0.793	0.368	0.974	0.535
100159-03	12.91	0.907	0.49	280.4	109.1	0.998	0.165	89.2	0.996	0.988	0.97	0.753	0.308	0.975	0.521
100159-04	13.67	0.916	0.5	267.1	100.8	0.998	0.174	98.6	0.999	0.992	0.975	0.799	0.367	0.975	0.549
100159-05	18.1	0.917	0.49	266	103.2	0.998	0.186	119.1	0.997	0.991	0.981	0.875	0.493	0.975	0.509
100160-02	16.24	0.912	0.5	278.4	107.8	0.998	0.19	110.3	0.999	0.993	0.981	0.842	0.44	0.975	0.529
100160-03	12.84	0.906	0.5	304.2	122.7	0.997	0.164	88.4	0.996	0.988	0.969	0.745	0.307	0.974	0.521
100160-07	15.46	0.923	0.5	253.7	93.6	0.998	0.19	111.9	0.997	0.99	0.979	0.851	0.451	0.973	0.562
100160-09	15.6	0.914	0.5	281.1	111.4	0.998	0.175	105.5	0.999	0.993	0.979	0.826	0.412	0.975	0.518
100163-01	26.77	0.948	0.48	274.1	114.6	0.999	0.227	235.7	0.999	0.995	0.992	0.976	0.898	0.978	0.729
100163-04	14.6	0.946	0.49	251.2	104.9	0.999	0.215	136.5	0.996	0.99	0.983	0.926	0.642	0.974	0.753
100164-01	17	0.92	0.5	257.5	97.3	0.998	0.223	107.5	0.999	0.993	0.98	0.815	0.407	0.976	0.515
100164-02	21.42	0.923	0.49	240.1	89.1	0.999	0.244	122	0.997	0.992	0.983	0.873	0.489	0.975	0.475
100164-03	18.63	0.917	0.5	255.5	95.1	0.998	0.218	115.9	0.999	0.994	0.983	0.848	0.454	0.976	0.503
100164-04	18.88	0.925	0.49	231.8	82.4	0.999	0.22	113.2	0.999	0.994	0.981	0.838	0.438	0.975	0.484
100193-01	13.74	0.947	0.49	231	91.6	0.999	0.223	131.7	0.996	0.99	0.982	0.919	0.617	0.974	0.772
100193-02	13.52	0.948	0.48	247	100.2	0.999	0.213	126	0.998	0.992	0.983	0.909	0.581	0.976	0.748
100207-01	13.86	0.918	0.49	263.3	97.2	0.997	0.238	117.3	0.999	0.993	0.984	0.898	0.526	0.979	0.729
100207-02	14.98	0.921	0.48	255.3	95.3	0.997	0.24	124.4	0.992	0.987	0.983	0.923	0.581	0.979	0.686
100207-03	13.79	0.91	0.48	291.6	109.8	0.996	0.241	118.2	0.992	0.987	0.982	0.908	0.532	0.978	0.711
100207-04	13.02	0.925	0.49	247.2	88.2	0.998	0.245	116.7	0.992	0.987	0.981	0.906	0.53	0.978	0.743
100210-01	12.63	0.942	0.48	276.9	120.1	0.999	0.216	111.1	0.996	0.989	0.979	0.88	0.483	0.975	0.716
100211-01	14.63	0.945	0.48	274.9	115.3	0.999	0.231	118.7	0.996	0.99	0.981	0.901	0.537	0.976	0.675
100211-02	15.44	0.941	0.49	291.1	121.3	0.999	0.242	125.5	0.999	0.993	0.986	0.912	0.573	0.979	0.69
100212-01	14.45	0.948	0.48	253.2	98.5	0.999	0.24	127	0.996	0.99	0.982	0.916	0.592	0.975	0.734
100212-02	15.69	0.942	0.47	278.9	122.1	0.999	0.239	113.4	0.999	0.992	0.982	0.886	0.498	0.978	0.608
100213-01	16.22	0.943	0.46	259.7	105.6	0.998	0.307	100	0.994	0.987	0.979	0.854	0.409	0.978	0.511
100213-02	16.4	0.946	0.47	251.1	100.6	0.999	0.306	105.3	0.994	0.988	0.98	0.871	0.45	0.978	0.53
100213-04	16.71	0.942	0.46	263.3	107.6	0.998	0.311	99.8	0.994	0.987	0.979	0.852	0.406	0.979	0.498
100215-01	16.16	0.944	0.48	243.8	90.3	0.998	0.242	125.2	0.994	0.989	0.983	0.92	0.587	0.978	0.584
100215-02	13.87	0.945	0.49	243.8	89.1	0.998	0.239	120.2	0.992	0.987	0.981	0.914	0.556	0.977	0.65
100215-03	14.44	0.948	0.48	228.8	82.6	0.998	0.244	117.6	0.993	0.988	0.982	0.904	0.54	0.978	0.616
100232-01	12.49	0.915	0.47	228.1	87.7	1	0.171	103.6	0.998	0.991	0.978	0.851	0.428	0.976	0.634
100232-02	11.99	0.913	0.47	234.5	89	1	0.17	101.4	0.998	0.991	0.978	0.846	0.412	0.976	0.65
100232-03	12.3	0.915	0.47	225.3	84	1	0.179	106.1	0.996	0.988	0.977	0.865	0.45	0.974	0.667
100232-04	14.03	0.917	0.46	222.9	85.4	1	0.176	105.8	0.999	0.991	0.979	0.86	0.445	0.976	0.578
100233-01	10.54	0.906	0.47	235.6	92	0.998	0.164	89.7	0.993	0.986	0.973	0.799	0.324	0.976	0.587
100233-02	11.36	0.908	0.47	227.9	88.8	0.998	0.169	85.9	0.992	0.984	0.97	0.784	0.292	0.974	0.523
100233-03	10.71	0.908	0.48	239.2	91.3	0.998	0.168	100.8	0.993	0.987	0.977	0.851	0.415	0.976	0.652
100233-04	11.1	0.897	0.48	263.1	101.4	0.998	0.171	103.9	0.993	0.987	0.978	0.862	0.435	0.977	0.65
100234-01	11.69	0.896	0.47	260.5	102.9	0.997	0.163	98.9	0.993	0.987	0.977	0.84	0.395	0.977	0.581
100234-02	11.32	0.896	0.48	260.3	102.9	0.997	0.167	97.7	0.992	0.984	0.975	0.84	0.385	0.975	0.593
100234-03	11.09	0.902	0.48	244.5	95	0.998	0.159	94.7	0.993	0.986	0.975	0.826	0.365	0.977	0.583
100235-01	9.76	0.894	0.48	272.7	105.7	0.997	0.162	92.8	0.993	0.986	0.974	0.815	0.349	0.976	0.652
100235-02	10.55	0.897	0.48	264.4	102.8	0.998	0.165	99.6	0.992	0.985	0.976	0.849	0.404	0.976	0.649
100235-03	11.76	0.907	0.48	235.9	90.1	0.998	0.179	103.2	0.992	0.985	0.976	0.861	0.435	0.975	0.615
100240-01	16.41	0.947	0.47	235.4	93.5	0.999	0.232	124.4	0.996	0.99	0.982	0.912	0.576	0.976	0.621
100240-02	13.53	0.949	0.49	246.2	95.5	0.999	0.236	121.2	0.996	0.99	0.981	0.906	0.557	0.976	0.742
100240-03	15.46	0.944	0.48	271	112.9	0.999	0.248	128.3	0.998	0.993	0.985	0.918	0.597	0.978	0.706
100241-01	14.45	0.943	0.48	269.6	117.4	0.999	0.219	117	0.999	0.993	0.984	0.896	0.525	0.979	0.661
100241-02	15.86	0.946	0.47	235.3	100.6	0.999	0.217	119.3	0.997	0.99	0.981	0.901	0.542	0.976	0.602
100241-03	12.7	0.945	0.48	273.1	124.1	0.999	0.204	103.9	0.998	0.991	0.98	0.857	0.432	0.978	0.654
100242-01	12.58	0.934	0.48	221.1	85.5	0.998	0.322	96.5	0.998	0.99	0.976	0.83	0.385	0.976	0.68
100242-02	12.77	0.935	0.48	220.5	86	0.998	0.327	98.8	0.996	0.988	0.975	0.842	0.401	0.975	0.691
100242-03	16.34	0.938	0.46	203.7	83.7	0.998	0.322	91.6	0.998	0.99	0.972	0.802	0.343	0.975	0.499
100242-04	13.48	0.931	0.47	221.5	92.6	0.998	0.317	86.4	0.996	0.987	0.97	0.785	0.301	0.974	0.566
100243-01	12.4	0.927	0.48	246.9	90.3	0.998	0.235	111.2	0.993	0.988	0.981	0.885	0.487	0.978	0.671
100243-02	12.54	0.924	0.48	247.6	90.1	0.997	0.247	111.6	0.991	0.986	0.98	0.891	0.492	0.977	0.675
100243-03	11.3	0.929	0.49	237.7	87.1	0.998	0.229	103.4	0.991	0.986	0.978	0.867	0.431	0.977	0.68
100244-01	11.58	0.915	0.47	235.3	92.9	1	0.166	93.4	0.998	0.99	0.974	0.812	0.353	0.976	0.616
100244-02	15.03	0.917	0.45	228.9	87.6	1	0.166	93.2	0.996	0.987	0.973	0.815	0.346	0.975	0.471
100244-03	10.82	0.914	0.48	229.2	85.6	1	0.174	101.5	0.996	0.988	0.976	0.848	0.415	0.974	0.724
100244-04	14.22	0.918	0.45	221.3	86.2	1	0.168	93.1	0.998	0.99	0.974	0.809	0.348	0.976	0.495
100245-01	13.53	0.946	0.48	263.3	114.4	0.999	0.218	111.3	0.996	0.99	0.98	0.885	0.488	0.976	0.668
100245-02	13.39	0.942	0.48	268.6	116.3	0.999	0.227	114.9	0.998	0.992	0.983	0.89	0.51	0.979	0.706
100247-01	13.54	0.942	0.48	268.6	119.1	0.999	0.214	108.9	0.998	0.992	0.981	0.872	0.467	0.978	0.651
100247-02	12.84	0.945	0.48	256.7	110	0.999	0.21	108	0.998	0.991	0.981	0.871	0.463	0.978	0.674
100247-03	14.39	0.947	0.48	256.3	109.5	0.999	0.239	120.6	0.996	0.99	0.981	0.903	0.546	0.975	0.697
100248-01	12.47	0.94	0.48	291.6	129.1	0.999	0.219	107	0.996	0.989	0.978	0.87	0.454	0.976	0.705
100249-01	11.87	0.949	0.48	225	91.5	0.999	0.193	101.8	0.998	0.991	0.978	0.847	0.418	0.977	0.661
100249-02	12.81	0.933	0.48	256.6	108.6	0.999	0.205	106.8	0.999	0.992	0.981	0.864	0.453	0.977	0.664
100250-01	15.89	0.946	0.48	249.3	104.9	0.999	0.24	129.9	0.998	0.993	0.985	0.917	0.602	0.978	0.68
100250-02	17.86	0.946	0.47	239.1	102.9	0.999	0.225	126.8	0.999	0.993	0.985	0.913	0.585	0.978	0.576
100250-03	14.42	0.946	0.48	263.4	111.3	0.999	0.228	121.6	0.998	0.992	0.983	0.902	0.553	0.976	0.695
100251-01	14.66	0.945	0.49	247.8	104.9	0.999	0.233	127.5	0.999	0.993	0.985	0.915	0.591	0.978	0.716
100252-01	15.7	0.945	0.48	271.2	118.5	0.999	0.238	127.9	0.999	0.993	0.985	0.916	0.59	0.978	0.682
100253-01	14.28	0.945	0.48	261.3	110.1	0.999	0.222	122.2	0.999	0.993	0.984	0.906	0.558	0.978	0.7
100253-02	18.58	0.944	0.48	250.5	107.7	0.999	0.226	139.6	0.999	0.994	0.987	0.932	0.653	0.979	0.615
100254-01	16.66	0.944	0.48	264.6	118.1	0.999	0.231	129.7	0.999	0.993	0.985	0.918	0.601	0.978	0.644
100254-02	15.84	0.944	0.48	268.4	114.3	0.999	0.235	126.9	0.999	0.993	0.985	0.915	0.585	0.978	0.669
100255-01	15.44	0.946	0.48	255.1	110	0.999	0.22	120	0.999	0.993	0.984	0.902	0.544	0.978	0.631
100256-01	18.84	0.941	0.48	279.1	126	0.999	0.265	130.8	0.999	0.993	0.986	0.921	0.603	0.978	0.609
100257-01	17.12	0.946	0.48	238.8	96.8	0.999	0.262	131.8	0.999	0.993	0.986	0.921	0.609	0.978	0.662
100257-02	16.14	0.945	0.48	256.9	108.7	0.999	0.255	126.4	0.999	0.993	0.985	0.912	0.577	0.978	0.672
100258-01	20.5	0.947	0.46	226.1	100.9	0.999	0.249	122.3	0.999	0.993	0.985	0.905	0.552	0.978	0.499
100258-02	16.68	0.946	0.49	247.5	103.4	0.999	0.251	137.3	0.999	0.993	0.986	0.929	0.64	0.978	0.699
100259-01	12.12	0.943	0.46	254	110.1	0.999	0.257	81.3	0.998	0.987	0.965	0.741	0.257	0.973	0.581
100259-02	12	0.94	0.48	272.3	114.9	0.999	0.257	94.6	0.998	0.99	0.974	0.818	0.361	0.976	0.686
100260-01	11.29	0.943	0.47	262.4	114	0.999	0.267	81.7	0.998	0.987	0.966	0.745	0.261	0.974	0.638
100269-01	14.59	0.933	0.46	256.6	104.1	0.999	0.238	92.9	0.996	0.987	0.972	0.81	0.344	0.974	0.54
100269-02	11.96	0.931	0.47	267.3	104.5	0.999	0.239	94	0.996	0.987	0.972	0.816	0.354	0.974	0.672
100269-03	11.33	0.935	0.47	248.3	95.1	0.999	0.235	87.3	0.998	0.988	0.968	0.774	0.303	0.974	0.65
100269-04	10.61	0.936	0.47	252.7	99.4	0.999	0.234	83.8	0.998	0.987	0.965	0.748	0.274	0.973	0.666
100271-01	11.76	0.937	0.47	246.9	98.7	0.999	0.23	89.2	0.998	0.989	0.97	0.784	0.317	0.975	0.634
100271-02	11.01	0.932	0.46	269.2	106.9	0.999	0.23	81.3	0.996	0.985	0.964	0.741	0.254	0.972	0.624
100271-03	12.62	0.931	0.47	273.9	108.3	0.999	0.238	96.5	0.996	0.988	0.974	0.828	0.372	0.974	0.653
100271-04	9.6	0.924	0.47	257.6	98.2	0.999	0.233	74	0.998	0.984	0.955	0.68	0.202	0.973	0.653
100272-01	13.2	0.939	0.44	216.8	96.1	0.999	0.155	77.8	0.999	0.999	0.997	0.874	0.162	0.998	0.335
100272-02	13.74	0.938	0.44	224.2	96	0.999	0.154	85.5	0.997	0.997	0.996	0.922	0.25	0.996	0.357
100272-03	13.92	0.941	0.44	214.3	91.2	0.999	0.167	82.5	0.999	0.998	0.997	0.904	0.215	0.998	0.343
100272-04	13.99	0.94	0.44	224.3	98.1	0.999	0.163	84	0.997	0.997	0.995	0.914	0.234	0.996	0.347
100272-05	12.62	0.943	0.44	212	87	0.999	0.15	80.9	0.999	0.998	0.997	0.899	0.197	0.998	0.365
100272-06	13.27	0.939	0.44	217.8	92.3	0.999	0.16	76.7	0.999	0.998	0.997	0.866	0.151	0.998	0.332
100273-01	13.56	0.94	0.45	221.7	94.2	0.999	0.164	85.6	0.997	0.997	0.996	0.92	0.257	0.996	0.366
100273-02	12.91	0.939	0.44	223.1	93.4	0.999	0.157	79.8	0.999	0.999	0.997	0.888	0.186	0.998	0.357
100274-01	13.39	0.935	0.46	239	91.4	0.999	0.212	89.7	0.996	0.987	0.971	0.795	0.321	0.975	0.544
100274-02	12.49	0.935	0.46	235.8	89.3	0.999	0.227	86.7	0.998	0.989	0.971	0.776	0.298	0.976	0.574
100275-01	9.94	0.933	0.47	247	89.2	0.999	0.213	85.7	0.998	0.988	0.969	0.769	0.293	0.974	0.707
100275-02	11.91	0.937	0.46	232.8	85.7	0.999	0.219	91	0.996	0.987	0.971	0.8	0.333	0.973	0.625
100276-01	15.31	0.94	0.46	235.6	100.7	0.999	0.175	110.4	0.999	0.992	0.982	0.882	0.494	0.977	0.556
100276-02	11.48	0.934	0.47	267.5	109.5	0.999	0.177	101.9	0.998	0.991	0.979	0.856	0.43	0.977	0.698
100276-03	15.09	0.943	0.46	226.8	95.2	0.999	0.175	113.9	0.996	0.99	0.98	0.893	0.521	0.976	0.578
100276-04	15.19	0.941	0.45	234.7	102.3	0.999	0.174	103.3	0.999	0.992	0.98	0.861	0.441	0.978	0.522
