# Supplementary material for: An accessible insight into genetic findings for transplantation recipients with suspected genetic kidney disease
Source: NPJ Genom Med. 2021 Jul 2;6:57. doi: 10.1038/s41525-021-00219-3 (PMC8253729; doi:10.1038/s41525-021-00219-3)
Supplement: Supplementary file 2 — Reporting Summary [file 41525_2021_219_MOESM2_ESM.pdf]

nature research

Corresponding author(s): Jiancheng GUO;Wenjun SHANG;Jia RAO

Last updated by author(s): Apr 12, 2021

Reporting Summary

Nature Research wishes to improve the reproducibility of the work that we publish. This form provides structure for consistency and transparency in reporting. For further information on Nature Research policies, see our Editorial Policies and the Editorial Policy Checklist.

Statistics

For all statistical analyses, confirm that the following items are present in the figure legend, table legend, main text, or Methods section.

n/a	Confirmed	
☒	☐	The exact sample size (*n*) for each experimental group/condition, given as a discrete number and unit of measurement
☒	☐	A statement on whether measurements were taken from distinct samples or whether the same sample was measured repeatedly
☒	☐	The statistical test(s) used AND whether they are one- or two-sided<br>*Only common tests should be described solely by name; describe more complex techniques in the Methods section.*
☒	☐	A description of all covariates tested
☒	☐	A description of any assumptions or corrections, such as tests of normality and adjustment for multiple comparisons
☒	☐	A full description of the statistical parameters including central tendency (e.g. means) or other basic estimates (e.g. regression coefficient) AND variation (e.g. standard deviation) or associated estimates of uncertainty (e.g. confidence intervals)
☒	☐	For null hypothesis testing, the test statistic (e.g. *F*, *t*, *r*) with confidence intervals, effect sizes, degrees of freedom and *P* value noted<br>*Give P values as exact values whenever suitable.*
☒	☐	For Bayesian analysis, information on the choice of priors and Markov chain Monte Carlo settings
☒	☐	For hierarchical and complex designs, identification of the appropriate level for tests and full reporting of outcomes
☒	☐	Estimates of effect sizes (e.g. Cohen's *d*, Pearson's *r*), indicating how they were calculated

Our web collection on statistics for biologists contains articles on many of the points above.

Software and code

Policy information about availability of computer code

Data collection	no software was used
Data analysis	Data analysis of exome sequencing was performed following the bioinformatics data analysis established in our laboratory which is described in detail in the Supplementary section S1.

For manuscripts utilizing custom algorithms or software that are central to the research but not yet described in published literature, software must be made available to editors and reviewers. We strongly encourage code deposition in a community repository (e.g. GitHub). See the Nature Research guidelines for submitting code & software for further information.

Data

Policy information about availability of data

All manuscripts must include a data availability statement. This statement should provide the following information, where applicable:

- Accession codes, unique identifiers, or web links for publicly available datasets
- A list of figures that have associated raw data
- A description of any restrictions on data availability

The pathogenic variants have been submitted to ClinVar (https://www.ncbi.nlm.nih.gov/clinvar/), and the submission number is SUB9604514. This study is compliant with the 'Guidance of the Ministry of Science and Technology (MOST) for the Review and Approval of Human Genetic Resources', which requires formal approval for the export of human genetic material or data from China.

Field-specific reporting

Please select the one below that is the best fit for your research. If you are not sure, read the appropriate sections before making your selection.

☒ Life sciences ☐ Behavioural & social sciences ☐ Ecological, evolutionary & environmental sciences

For a reference copy of the document with all sections, see nature.com/documents/nr-reporting-summary-flat.pdf

Life sciences study design

All studies must disclose on these points even when the disclosure is negative.

Sample size	A total of 115 families with 226 affected individuals were recruited from 576 family cohorts with records in the kidney transplantation registry since 2017 to 2019.
Data exclusions	Excluding cases with very characteristic features who were diagnosed based on clinical features and secondary causes which is described in detail in the Method section.
Replication	no replication available
Randomization	Cases were enrolled based on the enrollment criteria were as follows: (i) age at registry in the waiting list of kidney transplantation more than 18 years old; (ii) a family history of kidney disease which was defined as any family member with urinary abnormalities or impaired kidney function or undiagnosed kidney disease; or clinical suspicion of a genetic kidney disease owing to age onset less than 25 years old or extrarenal features
Blinding	no group in this study

Reporting for specific materials, systems and methods

We require information from authors about some types of materials, experimental systems and methods used in many studies. Here, indicate whether each material, system or method listed is relevant to your study. If you are not sure if a list item applies to your research, read the appropriate section before selecting a response.

Materials & experimental systems

n/a	Involved in the study	
☒	☐	Antibodies
☒	☐	Eukaryotic cell lines
☒	☐	Palaeontology and archaeology
☒	☐	Animals and other organisms
☐	☒	Human research participants
☐	☒	Clinical data
☒	☐	Dual use research of concern

Methods

n/a	Involved in the study	
☒	☐	ChIP-seq
☒	☐	Flow cytometry
☒	☐	MRI-based neuroimaging

Human research participants

Policy information about studies involving human research participants

Population characteristics	A total of 115 families with 226 affected individuals (male: female 1.4:1) were recruited from 576 family cohorts with records in the kidney transplantation registry. Consanguinity was observed in one family. Among the 115 families in this cohort, 104 families had multiple affected individuals checked for the records of urinalysis, renal function and imaging studies of the kidneys. There were 3 families with extrarenal features and a negative family history of renal disease. There were 8 patients who had early onset kidney disease without any extrarenal features or family history of renal disease. Among the 226 affected individuals, 75.7% (171/226) had proteinuria including 11.5% (26/226) with nephrotic proteinuria at disease onset. Abnormal image findings such as renal size or echogenicity were reported by renal ultrasound in 42.9% (97/226) of the cases. Renal biopsy was performed in 29.7% (68/226) of the affected individuals. Hearing loss was recorded in 16.8% (38/226) and vision deficiency was recorded in 4.4% (10/226). The median age at diagnosis of renal disease was 22.0 years old (interquartile range [IQR] 12.8-32.0 years old). The median age at genetic test was 31.0 years old (IQR 22.5-48.0 years old). For these families, at least one of the family members was on the transplant waitlist. At the time of transplant registration 125 (55.3%) probands developed into ESRD, four affected family members developed into CKD stage 4, six individuals developed into CKD stage 3, and six individuals developed into CKD stage 2. The median age at first renal replacement therapy was 28.2 years (range, 7.0–79.4 years). Overall, 11. 9% (27/226) of the patients in this cohort underwent renal transplantation.
Recruitment	The patients were referred for the evaluation and management of kidney disease and consented to a general genetic research program. Written informed consent offered the possibility to opt yes or no for disclosure of secondary findings,

	unrelated to the referral condition. Patients could choose if they wished to have their samples and/or data for future research, both anonymously or not. The samples of the affected individuals were subjected to exome sequencing (ES) of parent-child trios after the informed consent was obtained. Some of their unaffected family members as potential living donors were selected for ES post informed consent. ES procedure and variants annotation have been described in detail as shown in Section S1. Supplementary Methods.All the recruited families (n=115) with ESRD were divided into five subgroups according the prior clinical diagnosis of renal disease. ES was performed in 421 individuals from the 115 families (226 patients with CKD). Please see the details in Figure 1(A).
Ethics oversight	The criteria for genetic testing were approved by the ethics committees of the First Affiliated Hospital of Zhengzhou University (No. 2017-KY-106).

Note that full information on the approval of the study protocol must also be provided in the manuscript.

Clinical data

Policy information about clinical studies

All manuscripts should comply with the ICMJE guidelines for publication of clinical research and a completed CONSORT checklist must be included with all submissions.

Clinical trial registration	The criteria for genetic testing were approved by the ethics committees of the First Affiliated Hospital of Zhengzhou University (No. 2017-KY-106).
Study protocol	Please see the details shown as Figure 1 a.
Data collection	Data collect from Jan 2017 and Dec 2019.
Outcomes	clinical management and outcomes were devided by "Dialysis" and "Transplantation".
